# Structure-guided identification of denitrification inhibitors to mitigate agricultural N_2_O emissions

**DOI:** 10.1038/s41467-026-76975-6

**Published:** 2026-08-20

**Authors:** Yue Deng, Haixia Zeng, Lin Zhang, Bin Liu, Linfa Fang, Hailin Zhang, Yongliang Yang, Ran Xiao, Lijun Hou, Jinbo Zhang, Fusuo Zhang, Prakash Lakshmanan, Yingmu Wang, Yong-guan Zhu, Xiaoxuan Su, Xinping Chen

**Affiliations:** 1 Interdisciplinary Research Center for Agriculture Green Development in Yangtze River Basin, College of Resources and Environment, Southwest University, Chongqing, China; 2 Key Laboratory of Low-carbon Green Agriculture in Southwestern China, Ministry of Agriculture and Rural Affairs, Chongqing, China; 3 Institut für Biochemie, Albert-Ludwigs-Universität Freiburg, Freiburg, Germany; 4 State Key Laboratory of Nutrient Use and Management, China Agricultural University, Beijing, China; 5 Center for Molecular Medicine, School of Life Science and Biotechnology, Dalian University of Technology, Dalian, China; 6 State Key Laboratory of Estuarine and Coastal Research, East China Normal University, Shanghai, China; 7 School of Breeding and Multiplication, Hainan University, Sanya, China; 8 Liebig Centre for Agroecology and Climate Impact Research, Justus Liebig University, Giessen, Germany; 9 Queensland Alliance for Agriculture and Food Innovation, University of Queensland, St Lucia, QLD Australia; 10 College of Civil Engineering, Fuzhou University, Fuzhou, China; 11 Research Center for Eco-Environmental Sciences, Chinese Academy of Sciences, Beijing, China; 12 University of Chinese Academy of Sciences, Beijing, China

**Keywords:** Element cycles, Climate-change mitigation, Agroecology

## Abstract

Reducing anthropogenic nitrous oxide emissions from agricultural lands is critical to combating climate change. Nitrification inhibitors and synthetic microbial consortia have already demonstrated potential in decreasing soil nitrification-derived nitrous oxide emissions. However, targeted interventions addressing denitrification sources while balancing agricultural sustainability remain challenging. Here, we identify triazole compounds as cytochrome P450 inhibitors capable of reducing soil denitrification-derived nitrous oxide emissions by 85-100% in laboratory settings. Extending our findings to in-situ paddy fields, we observe that triazoles (at 0.05% of fertilizer nitrogen) reduce nitrous oxide emissions by 33-54%. These potential inhibitors specifically target the NAD(P)H and nitric oxide binding sites of P450Nor, strongly inhibiting fungal nitrous oxide emissions. Occupancy of the heme-iron(III) site within the P450Nor active pocket enhances this selectivity, which is critical for effective inhibition. Given the projected increase in agricultural nitrous oxide emissions, our findings offer insights into the development of denitrification inhibitors and highlight triazole as a promising solution for nitrous oxide mitigation in agricultural ecosystems.

## Abstract (web summary)

A key gap in agricultural climate mitigation is the lack of interventions that specifically target denitrification. This study identifies that triazole compounds act as cytochrome P450 inhibitors, effectively reducing N₂O emissions from denitrification.

## Introduction

The industrial synthesis of ammonia via the Haber–Bosch process has transformed modern agriculture by enabling the large-scale production of reactive nitrogen-based chemical fertilizers, thereby increasing crop yields and contributing to global food security^1^. However, the excessive and often unregulated use of these fertilizers, driven by minimal economic consequences for nitrogen release into ecosystems, has resulted in severe environmental challenges^2,3^. Numerous agricultural ecosystems worldwide have become heavily enriched with reactive nitrogen, leading to significant emissions of nitrous oxide (N_2_O), a potent greenhouse gas and ozone-depleting substance^4–7^. Fertilizer-driven N_2_O emissions now account for more than half of the total anthropogenic N_2_O emissions^8,9^, highlighting agricultural soils as a major source of N_2_O emissions over recent decades^10–12^.

While reducing nitrogen inputs via optimized fertilization is a viable mitigation strategy, a more direct route lies in regulating the specific N_2_O‑generating reactions of nitrification and denitrification. Process‑targeted inhibitors represent a promising first step in this regard. Nitrification inhibitors, including dicyandiamide (DCD), Nitrapyrin, and 3,4-dimethylpyrazole phosphate (DMPP), have been used to suppress nitrification-derived N_2_O emissions, particularly in intensively fertilized agricultural systems in China^13,14^. However, no inhibitors have been reported or utilized to target denitrification-derived N_2_O emissions to date.

Biotic denitrification, mediated primarily by bacteria and fungi, is a major source of N_2_O emissions in soils^8,15–17^. Although some denitrifying bacteria possess the complete denitrification pathway, in natural settings, denitrification commonly occurs modularly, particularly through incomplete denitrifiers^18^. Bacterial denitrifiers lacking N_2_O reductase (NOS, the product of the *nosZ* gene) can therefore act as direct sources of N_2_O production^19^. Furthermore, all currently known denitrifying fungi lack NOS, making N_2_O the final product of fungal denitrification and further exacerbating soil N_2_O emissions^20^. Hence, identifying regulators that concurrently inhibit fungal and bacterial N_2_O production is crucial for the reduction.

In fungal denitrifying machinery, P450Nor catalyzes the key step of N_2_O production, making it a promising target for mitigating fungal N_2_O emissions. P450Nor is an ancient member of the cytochrome P450 (CYP) families^21^, which features a cys-coordinate heme (HEM) at its active site^22^. The HEM pocket of P450Nor comprises amino acid residues A239, N246, T234, A284, S286, D393, and V394^23^. Mechanistically, P450Nor reduces NO to N_2_O using NAD(P)H as a proton donor, delivered by the hydrogen-bonding network involving H_2_O (W74)-S286-H_2_O (W33)-D393 proton pathway. The reaction proceeds through the formation of Fe^3+^-NO, followed by the creation of intermediate ***I*** (Fe^3+^-NHO) through NAD(P)H oxidation. The intermediate ***I*** subsequently recruits a second NO molecule, enabling N-N bond formation and N_2_O production^24,25^. Although the catalytic mechanism of P450Nor has been elucidated, strategies for selectively inhibiting this enzyme remain limited. Targeting the HEM-Fe^3+^ center of P450Nor provides a direct route to suppressing its catalytic activity, as suggested by the studies of CYP-inhibitor clinical research^26–28^. Interactions between CYP enzymes and nitrogenous drugs, particularly triazoles, have led to the development of various therapeutic agents^29^, including anastrozole and letrozole targeting aromatase (CYP19) for the treatment of postmenopausal breast cancer^30^, and uniconazole and paclobutrazol inhibiting lanosterol demethylase (CYP51A1) as antifungal agents^31^. These precedents suggest that triazole-based inhibitors may also modulate P450Nor activity by occupying the HEM-Fe^3+^ center, thereby preventing the formation of its intermediate ***I***. However, whether triazole compounds can directly inhibit fungal denitrification and reduce N_2_O emissions from agricultural soils remains unknown.

With these considerations, here we select a common triazole compound 5-[(4-chlorophenyl)methyl]−2,2-dimethyl-1-(1,2,4-triazol-1-ylmethyl)cyclopentan-1-ol (C_17_H_22_ClN_3_O, Mez), aiming to evaluate its potential in reducing agricultural N_2_O emissions at microbial protein, cell, community, and field levels, as well as exploring the mitigation mechanisms. To achieve this, we first assess Mez’s impacts on fungal and bacterial N_2_O emissions through the substrate-induced respiration inhibition (SIRIN) method. Secondly, we integrate metagenomics coupled with metagenome-assembled genomes and metaproteomics to decode adaptation strategies of *nosZ*-harbored bacterial denitrifiers to triazoles. Thirdly, through molecular docking and dynamics simulation paired with surface plasmon resonance (SPR) of Mez and P450Nor protein, we explore the inhibition mechanisms by which Mez reduces fungal N_2_O emissions. Fourthly, based on the binding model of Mez with the P450Nor active pocket, another triazole compound, 1-[[2-[2-chloro-4-(4-chlorophenoxy)phenyl]−4-methyl-1,3-dioxolan-2-yl]methyl]−1,2,4-triazole (C_19_H_17_Cl_2_N_3_O_3_, Dif), is further selected to compare its selectivity and inhibitory effects on P450Nor against Mez, aiming to screen for a better fungal denitrification inhibitor. Finally, we conduct pure-culture, microcosm, and in-situ (paddy fields) experiments to validate the inhibition efficiency of these two triazole compounds (Mez and Dif) on N_2_O emissions. This study pioneers a methodology for developing denitrification inhibitors, offering a strategic intervention to manage elevated N_2_O emissions within agricultural ecosystems.

## Results

### Soil N_2_O emissions in response to Mez application

**Fig. 1:**
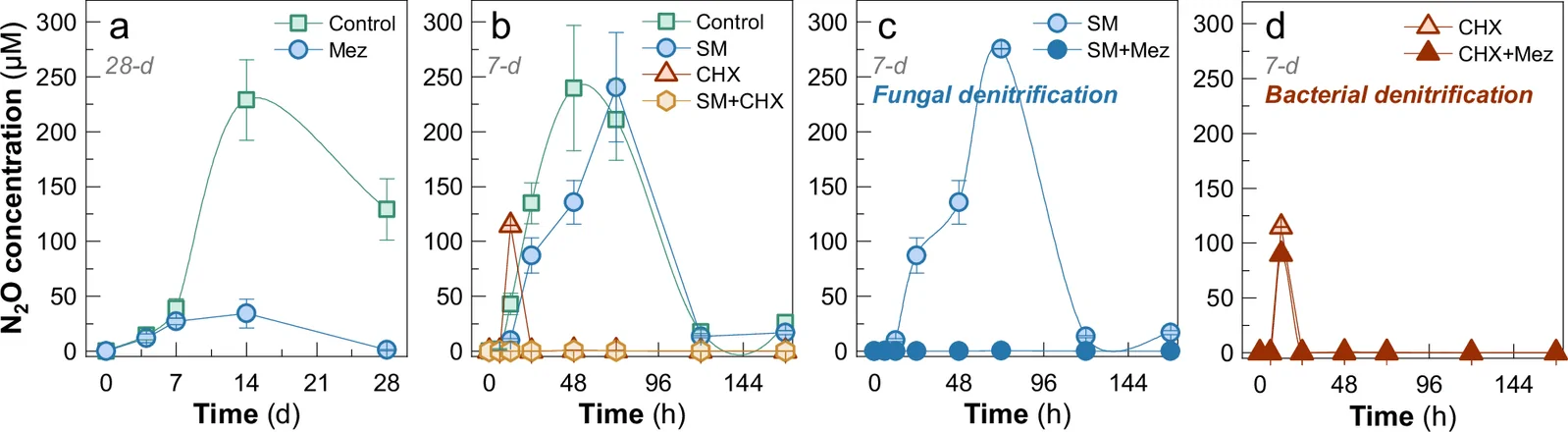
Variations of N_2_O concentrations in response to Mez in agricultural soils. **a** Changes in N_2_O concentrations over a 28-day incubation. **b** Changes in N_2_O concentrations over a 7-day incubation (without Mez) under bacterial (streptomycin, SM) and fungal (cycloheximide, CHX) inhibitors to differentiate bacterial and fungal N_2_O emissions. **c** Changes in fungal denitrification-derived N_2_O emissions in response to Mez during a 7-d incubation. **d** Changes of bacterial denitrification-derived N_2_O emissions in response to Mez during a 7-d incubation. Data are presented as mean ± SD (biological replicate, *n* = 4).

To study the effects of metconazole (Mez) on soil denitrification and N_2_O emissions, we performed a 28-d microcosm incubation. During the incubation, Mez residues and the concentrations of NO_3_^*-*^, NO_2_^-^, NH_4_^+^, and N_2_O were monitored. Mez, applied at 1 mg/kg, dissipated during the incubation (Supplementary Fig. 1), and its dissipation fitted a first-order kinetic model, with an estimated half-life of 9.45 d. The presence of Mez was associated with increased NO_3_^-^ and NO_2_^-^ reduction and NH_4_^+^ production, suggesting a potential stimulation of the soil dissimilatory NO_3_^-^ reduction to NH_4_^+^ process (DNRA; Supplementary Fig. 2a–d). More importantly, we found that N_2_O emissions were significantly reduced by Mez (*F* = 116.39, *P* < 0.001), with the inhibition ranging from 85 to 100% (Fig. 1a). These results suggest that Mez effectively mitigates soil N_2_O emissions at environmentally relevant concentrations.

To further distinguish the effects of Mez on fungal versus bacterial denitrification contributions to N_2_O emissions, we conducted an additional microcosm incubation with the additions of bacterial (streptomycin, SM) and fungal (cycloheximide, CHX) inhibitors (Fig. 1b–d). In the absence of Mez, SM did not significantly alter soil N_2_O emissions compared with the control (*F* = 4.36, *P* = 0.065, Fig. 1b). By contrast, CHX addition significantly reduced N_2_O emissions (*F* = 29.52, *P* = 0.019, Fig. 1b, and Supplementary Fig. 2f), highlighting the predominant role of soil fungal N_2_O emissions. Consistent with the 28-d incubation results, Mez significantly reduced the total N_2_O emissions (*F* = 298.71, *P* < 0.001, Supplementary Fig. 2e), and it notably inhibited N_2_O emissions by fungi (*F* = 187.29, *P* < 0.001, Fig. 1c, d). These findings indicate that Mez selectively targets fungal N_2_O production, leading to an overall N_2_O reduction.

### Targeted inhibition of Mez on fungal N_2_O emissions

**Fig. 2:**
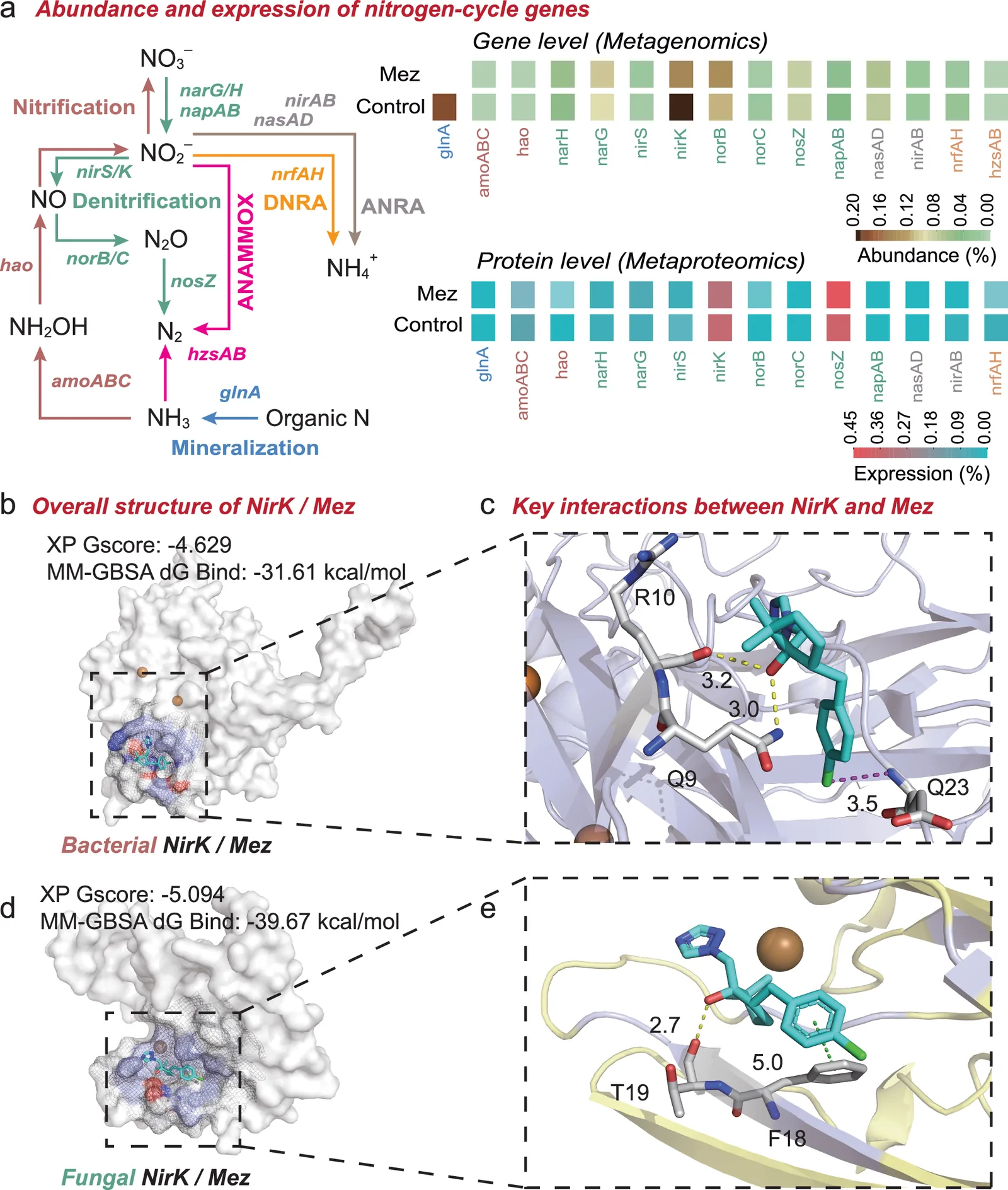
Abundances of N-cycling genes and the structure-based model of Mez binding with NirK. **a** Gene and protein abundances of each N transformation in the control and Mez treatments. **b**, **d** Overall structures of the bacterial NirK/ Mez complex (PDB code: 6ZAS) and fungal NirK/ Mez complex (PDB code: 6THE). NirK proteins are represented as surface mode. Mez is shown as sticks (cyan). **c**, **e** Chemical structures of Mez with predicted binding mode. Hydrogen bonds (yellow), Halogen bonds (violet), and π-π stacking interactions (green) between the amino group of Mez (cyan) and NirK (grey) in the binding pocket are shown.

To investigate the underlying mechanisms by which Mez reduced soil N_2_O emissions, we performed metagenomics and metaproteomics. We examined the genes and their expression levels across the major nitrogen transformation pathways, including mineralization, nitrification, denitrification, anammox,‌ dissimilatory nitrate reduction to ammonium (DNRA), and assimilatory nitrate/nitrite reduction to ammonium (ANRA) (Fig. 2a). Notably, we found that Mez reduced the relative abundance of the *nirK* gene from 21.93 to 17.82%, and its expression also decreased from 34.11 to 31.98% (Fig. 2a), which contrasts with the observed enhancement of NO_2_^-^ reduction by Mez (Supplementary Fig. 2b). At the community level, Mez had no significant effect on soil archaeal community composition and structure (Supplementary Fig. 3), suggesting that archaea were unlikely to be primary microbial targets responsible for the observed reduction in N_2_O emissions. Because both bacterial and fungal denitrifiers can possess copper-containing NO_2_^-^ reductase (NirK), we next sought to examine whether Mez interacts differently with bacterial and fungal NirK enzymes. However, fungal genome assembly was unsuccessful in this study, probably owing to the prevalence of non-coding sequences within fungal genomes^32^. We therefore used molecular docking to assess the potential interactions between Mez and representative bacterial and fungal NirK proteins.

Bacterial NirK is a typical homotrimer, with each monomer consisting of two distinct copper centers: a type 1 (T1Cu) center, buried approximately 4–6 Å beneath the protein surface within one of the two β-barrel domains, which functions as the electron entrance point, and a T2Cu center, positioned at the monomer interfaces and serving as the NO_2_^-^ reduction site^33^. Molecular docking of the bacterial NirK/Mez complex showed the Extra Precision (XP) Gscore and Molecular Mechanics/Poisson Boltzmann (Generalized Born) Surface Area (MM-GBSA) ΔG Bind were −4.629 and −31.61 kcal/mol, respectively (Fig. 2b, c). In the predicted bacterial NirK/Mez complex, Mez was situated on the protein surface, distant from both copper centers (Supplementary Fig. 4a). This binding position suggests that Mez is unlikely to directly interfere with electron transfer or NO_2_^-^ binding in bacterial NirK.

Fungal NirK contains a T1Cu coordinated by one cysteine, one methionine, and two histidine residues, and a T2Cu coordinated by two histidine residues from one monomer and another histidine from an adjacent monomer^34^. Molecular docking of the fungal NirK/Mez complex showed an XP Gscore and MM-GBSA ΔG Bind of −5.094 and −39.67 kcal/mol, respectively, indicating a more favorable predicted interaction than that observed for bacterial NirK (Fig. 2d, e). Nevertheless, the predicted pattern of Mez differs markedly from that of the native ligand (Supplementary Fig. 4b), suggesting that fungal NirK may not be the primary inhibitory target of Mez. We therefore further examined P450Nor, which facilitates the reduction of NO to N_2_O in fungal denitrifying systems.

**Fig. 3:**
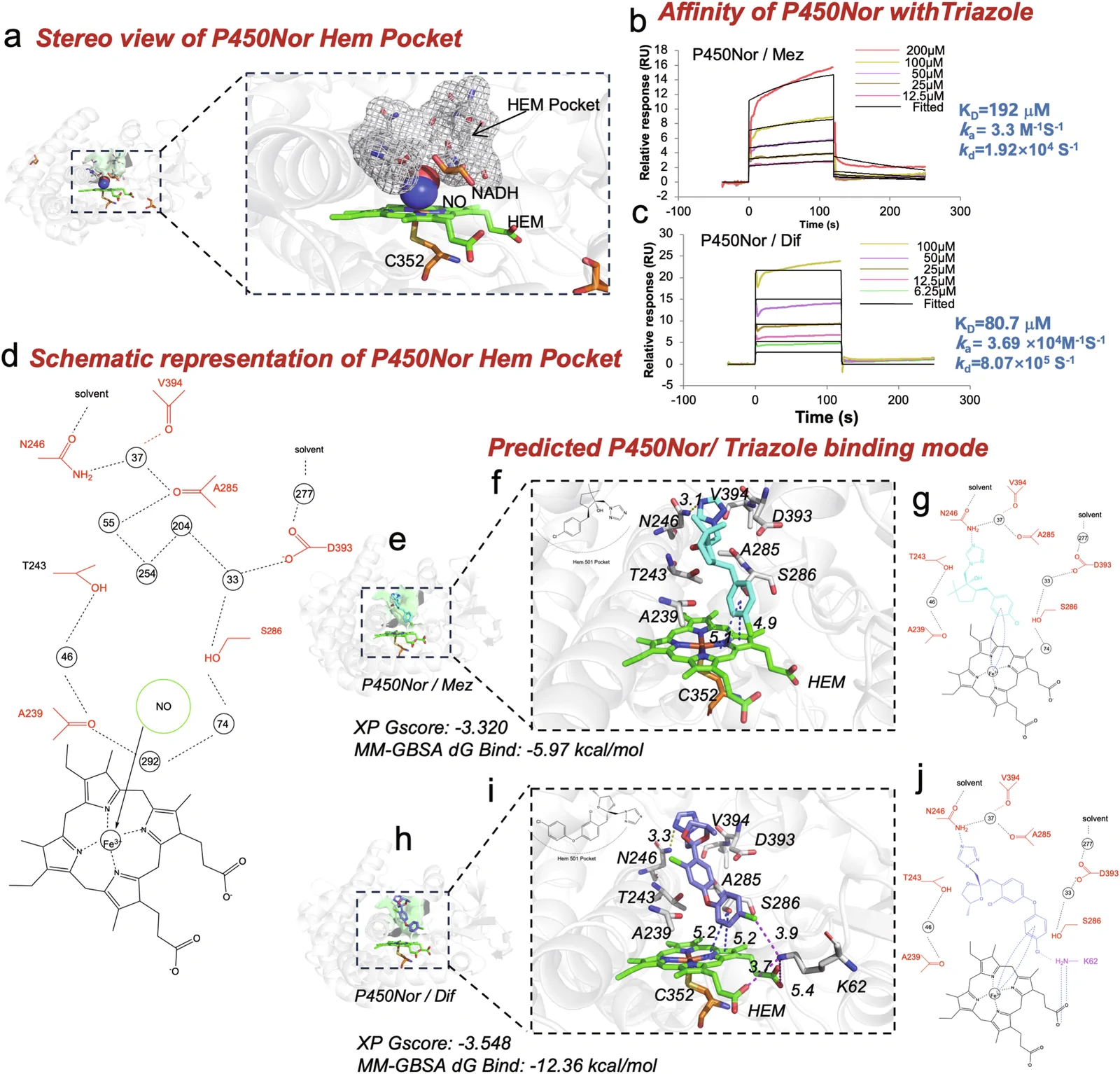
The structure-based model of triazoles binding with P450Nor. **a** Stereo view of the HEM iron-NO coordination structure of the P450Nor in the ferric NO state. **b** Confirmation of P450Nor binding with Mez by SPR. **c** Confirmation of P450Nor binding with Dif by SPR. **d** Schematic representation of the top view of HEM pocket of P450Nor, showing the hydrogen-bonding networks in HEM active site. **e**, **h** Overall structures of the P450Nor / Mez or Dif complex (PDB code: 7DVO). P450Nor is represented as surface mode. Mez (cyan) and Dif (violet) are shown as sticks. **f**, **i** Hydrogen bonds (yellow), π-π stacking interactions (blue), and Halogen bonds (violet) between the amino group of Mez (cyan) or Dif (violet) and P450Nor (grey) in the binding pocket. **g**, **j** Schematic representation of the top view of the HEM pocket of P450Nor binding with Mez (cyan) and Dif (violet).

P450Nor contains a cysteine-coordinated heme (HEM) at its active site (Fig. 3a). Docking of Mez with fungal P450Nor showed an XP Gscore and MM-GBSA of −3.320 and −5.97 kcal/mol, respectively (Fig. 3e). In the predicted P450Nor-Mez complex, Mez fits within the ligand (NO)-binding cavity and forms π-π stacking interactions with HEM and a hydrogen bond (H-bond) with N246 residue (Fig. 3f, g). These findings collectively indicate that P450Nor is a likely target of Mez in fungal denitrification. The predicted binding mode of Mez in P450Nor is also consistent with the HEM-centered binding patterns reported for several CYP inhibitors, hinting at the potential of triazoles to regulate fungal denitrification by targeting P450Nor.

### Mez-associated changes in bacterial N_2_O-reducing denitrifiers

**Fig. 4:**
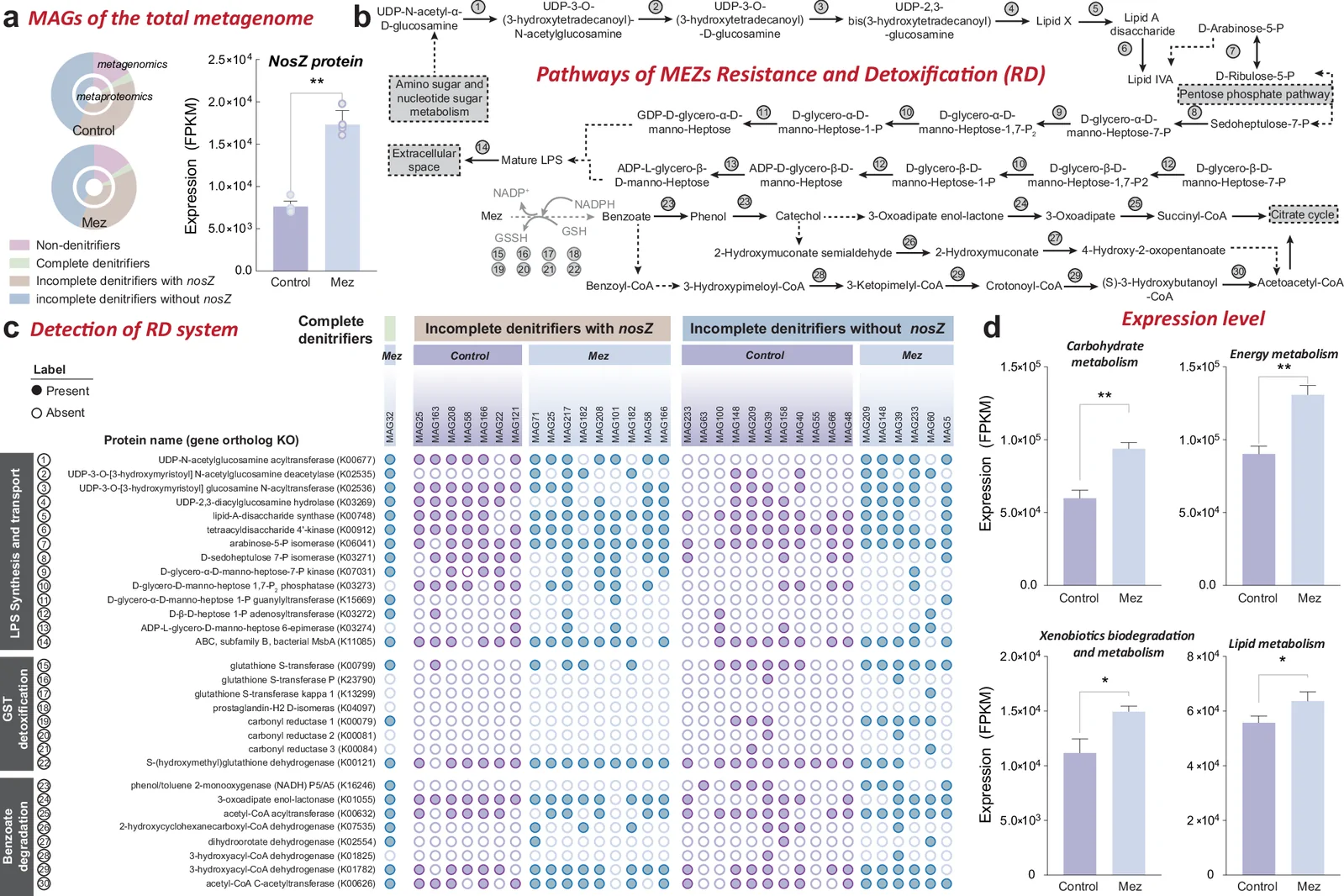
Proportions of denitrifying MAGs and postulated pathways of Mez resistance and detoxification (RD) from bacterial MAGs associated with the denitrification process. **a** Proportions of non-denitrifiers, complete denitrifiers, and incomplete denitrifiers with and without *nosZ* in the MAGs of the total metagenomes, as well as expression levels of NosZ protein. Data are presented as mean ± SD (biological replicate, *n* = 4). **b** Postulated pathways for bacterial Mez resistance and detoxification (RD) systems. **c** Overview of the completeness of Mez RD systems in complete and incomplete denitrifiers. **d** Protein-level expression of the Mez RD system. Asterisk (*) indicates statistical significance (one-way ANOVA, ^*^P < 0.05, ^**^P < 0.01). Data are presented as mean ± SD (sample numbers, *n* = 14~91; details see Supplementary Data 2).

We further conducted metagenome binning and mapped the metaproteome to individual metagenome-assembled genomes (MAGs) to explore Mez impact on bacterial denitrifiers. A total of 186 and 201 medium- and high-quality (completeness >50% and contamination <10%) MAGs were recovered from the control and Mez treatments, respectively. Abundances of each MAG at the gene and expression levels are depicted (Supplementary Fig. 5, and Supplementary Data 1). These MAGs spanned 20 bacterial phyla and were dominated by *Pseudomonadota*, *Gemmatimonadota*, *Patescibacteria*, and *Bacteroidota*. Most MAGs contained at least one denitrifying gene. Mez significantly altered the denitrifier compositions (Supplementary Fig. 6), increasing the relative abundances of *nirK*-harboring MAGs affiliated with *Pseudomonadota* and *Bdellovibrionota*, as well as *nosZ*-harboring MAGs affiliated with *Pseudomonadota*, suggesting their higher tolerance to Mez. Among the denitrifier MAGs, 12 were identified as complete denitrifiers, whereas 184 were incomplete denitrifiers, with over one third harboring the *nosZ* gene (Fig. 4a). The expression levels of *nirK* and *nosZ* genes in each MAG are shown in Supplementary Data 1. Particularly, Mez significantly increased NosZ expression in most MAGs (*F* = 83.75, *P* < 0.001, Fig. 4a, and Supplementary Data 1), explaining the observed decrease in bacterial N_2_O emissions through enhanced N_2_O reduction.

Comparative analysis of the central metabolic pathways between denitrifying MAGs from control and Mez treatments revealed metabolic adaptations and niche shifts in response to Mez. Denitrifiers exposed to Mez possessed more complete carbohydrate metabolism, energy metabolism and xenobiotic biodegradation pathways compared to control (Supplementary Fig. 7), indicating a metabolic advantage under Mez-induced stress. Particularly, incomplete denitrifiers with *nosZ* gene in Mez-treated samples exhibited more elaborate core metabolic pathways than their *nosZ*-negative counterparts.

Genes potentially associated with Mez resistance and detoxification (RD) were also enriched in abundant MAGs. The putative Mez RD system comprised four critical components: lipopolysaccharides (LPS) synthesis, ATP-binding cassette (ABC) transporters, glutathione S-transferase (GST) detoxification, and benzoate degradation pathways (Fig. 4b). This system may reduce intracellular Mez accumulation through LPS-mediated cell-envelope protection and ABC transporter-mediated efflux, followed by Mez transformation and metabolism via GST detoxification and benzoate degradation pathways. On this basis, we propose a hypothetical intracellular Mez degradation route in which Mez is first conjugated and detoxified by GST-related enzymes, generating aromatic intermediates that retain benzene-ring moieties (Supplementary Fig. 8); these intermediates may then be channelled into benzoate degradation pathways for further ring cleavage and catabolism. The presence of RD system in denitrifiers indicates their evolutionary adaptation to Mez, with complete denitrifiers exhibiting more comprehensive and energy-intensive routes for detoxification and degradation (Fig. 4c).

By contrast, abundant incomplete denitrifiers harboring *nosZ* gene appeared to adopt an energy-saving survival strategy (Fig. 4c). These denitrifiers exhibited greater Mez resistance by increasing LPS synthesis and ABC transporter gene enrichment, while having a more streamlined Mez detoxification system, characterized by fewer genes enriched in GST detoxification and benzoate degradation pathways, compared to their *nosZ*-negative counterparts (Fig. 4c). Mez application also enhanced lipid metabolism and xenobiotic biodegradation and metabolism capacities in these prevalent denitrifiers, significantly upregulating expressions of these two pathways (*F* = 15.23~121.43, *P* = 0.041 ~ <0.001, Fig. 4d). Moreover, Mez facilitated carbohydrate (*F* = 28.10, *P* = 0.0018) and energy (*F* = 281.93, *P* < 0.001, Fig. 4d) metabolism in denitrifiers, improving carbon source utilization and energy cycling efficiency, which may increase bacterial N_2_O reduction.

### Structure-guided identifying Dif as a better P450Nor inhibitor

As Mez was associated with increased bacterial N_2_O-reduction potential but reduced fungal-associated N_2_O emissions, it is worth exploring whether more effective denitrification inhibitors exist. The structural plasticity of the P450Nor HEM substrate binding cavity (Fig. 3a, b, and Supplementary Data 2) presents a challenge for the rational design of effective small-molecule inhibitors. Our molecular docking results indicate that Mez bound to the N246 residue, engaging in π-π stacking interactions with HEM, potentially disrupting the formation of the HEM Fe^3+^-NO coordination structure essential for P450Nor function. To examine the conformational dynamics and stability of the P450Nor-Mez binding mode predicted by molecular docking, we performed molecular dynamics (MD) simulations over nanosecond timescales. Mez demonstrated stable binding to P450Nor, maintaining an orientation consistent with the initial docking predictions throughout the simulations. The Root Mean Square Deviation (RMSD) value, which reflects ligand-protein complex stability^35^, remained within 2.5 Å (Supplementary Fig. 9a), signifying a stable interaction. Key residues R174, A239, T243, N246, A285, R392, and D393 engaged frequently with Mez (Supplementary Fig. 10a, b). Specifically, A285, T243, and A239 residues formed H-bonds with the hydroxyl of Mez; R174, N246, R392, and D393 residues formed water bridges with the hydroxyl and 1,2,4-triazole moiety of Mez, respectively; and R392 residue formed π-cation interaction with 1,2,4-triazole moiety of Mez (Supplementary Fig. 10). Surface plasmon resonance (SPR) determined a dissociation constant (*K*_*D*_) of 192 μM for Mez binding to P450Nor, providing experimental support for this interaction (Fig. 3b). The MD simulation further suggested that Mez may disrupt the proton delivery pathway of P450Nor by forming water bridge interactions with S286 and D393 residues and prevent NO binding to HEM-Fe^3+^ by forming H-bond with A239 and T243 residues. However, no direct interaction was predicted between HEM-Fe^3+^ and Mez, reducing the affinity of P450Nor with Mez. We therefore hypothesized that 1,2,4-triazole derivatives capable of occupying the HEM-Fe^3+^ center would exhibit stronger binding to P450Nor and greater potential to inhibit fungal denitrification.

To test this possibility, we conducted molecular docking with triazole compounds commonly used in agriculture. Among the tested compounds, difenoconazole (Dif) displayed the most favorable predicted interaction with P450Nor. The P450Nor-Dif docking model showed a halogen bond between the chlorine atom of Dif and residue L62, in a position that could restrict access to HEM-Fe^3+^ center (Fig. 3h–j). The docking score, including the XP Gscore (3.548) and MM-GBSA ΔG Bind (−12.36 kcal/mol), indicated that Dif exhibited a higher binding affinity for P450Nor than Mez (Fig. 3h). MD simulations further confirmed that the chlorine atom of Dif coordinated with HEM-Fe^3+^, forming stable interactions with C352 (Supplementary Fig. 11a). The RMSD of P450Nor-Dif complex remained within 2.5 Å, supporting the conformational stability of the predicted complex during the simulation (Supplementary Fig. 9b). Key residues, including R64, A79, Q231, F234, L235, A239, and C352, engaged more frequently with Dif (Supplementary Fig. 11). Notably, the 1,2,4-triazole moiety of Dif formed a H-bond with R64 and π-π stacking interactions with F234. The SPR analysis determined a *K*_*D*_ of 80.7 μM for the P450Nor-Dif interaction (Fig. 3c and Supplementary Data 2), indicating stronger binding of Dif to P450Nor than that observed for Mez under the same conditions. Together, these molecular docking, MD, and SPR results collectively identify Dif as a candidate inhibitor for fungal denitrification.

### Validation of Dif inhibition on fungal N_2_O emissions

**Fig. 5:**
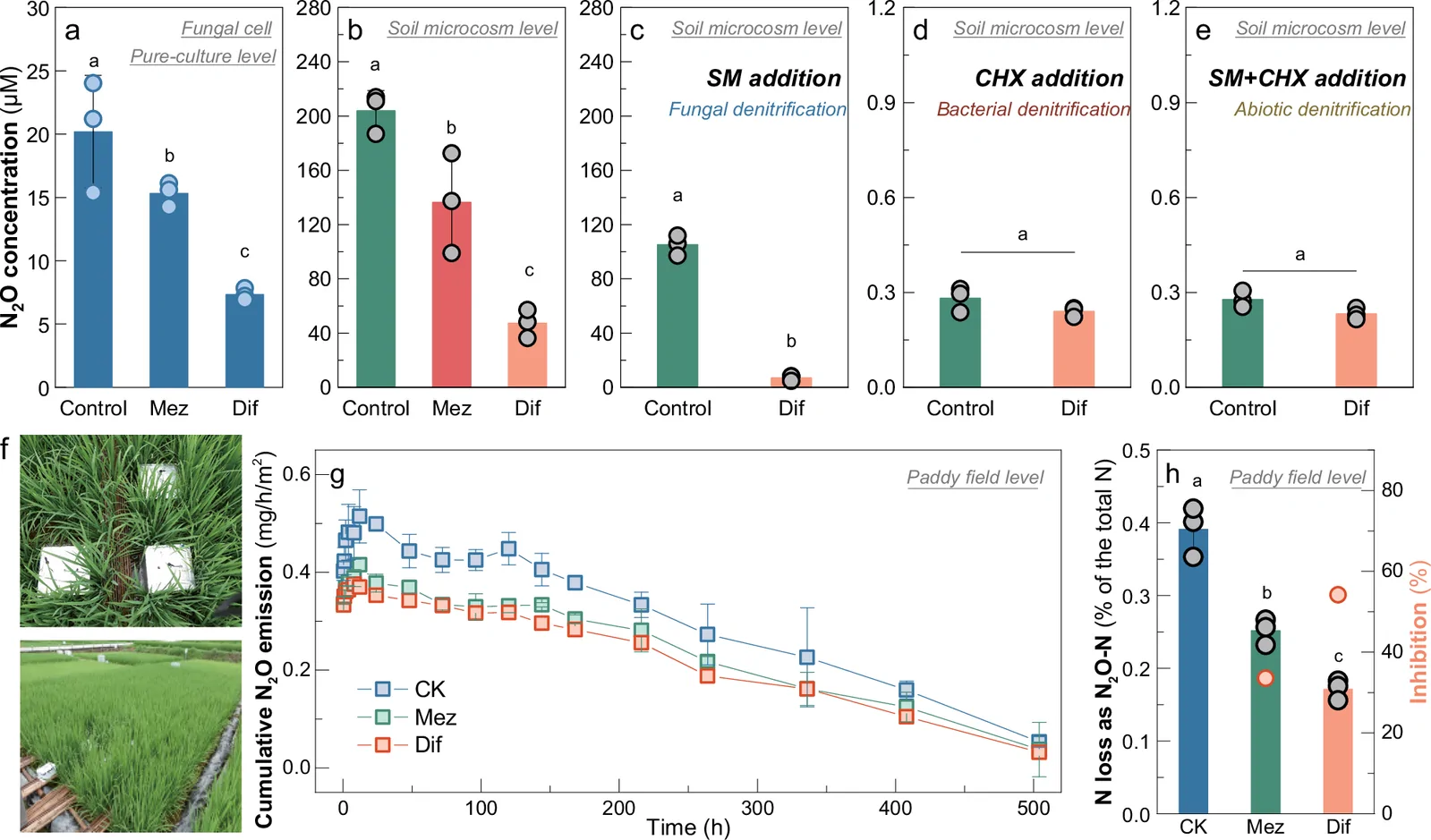
Validation of Dif inhibition on N_2_O emissions at pure-culture, soil microcosm, and in-situ paddy field levels. **a** Pure-culture level: Triazoles (Mez and Dif) inhibition on N_2_O production by the denitrifying fungus *P. janthinellum* SM-12f4. **b**–**e** Soil microcosm level: Changes in soil N_2_O emissions in response to Dif during a 7-day incubation with bacterial and/or fungal inhibitors. Paddy field level: Photos of static chambers in in-situ paddy fields (**f**); Changes in N_2_O fluxes in response to Mez and Dif over the 21-day experiments (**g**); The total inhibition efficiencies (%) of Mez and Dif on soil N_2_O emissions (**h**). The N loss (%) is assessed as the ratio of N_2_O-N to total N content (49.22 kg N ha^-1^) in the fertilizer. The inhibition efficiency is estimated by the ratio of (N loss in the control – N loss in the Mez/Dif) / (N loss in the control). Different letters indicate statistical significance (one-way ANOVA, *P* < 0.05). Data are presented as mean ± SD (biological replicate, *n* = 3).

To compare and validate the inhibitory performance of Dif and Mez on N_2_O emissions, we conducted experiments using pure cultures of an isolated fungal denitrifier *Penicillium janthinellum* SM-12f4X (7-d incubation), soil microcosms (7-d), and paddy field experiments (21-d), respectively (Fig. 5). In the pure-cultural assays, the denitrifying fungus *P. janthinellum* SM-12f4X was treated with Mez and Dif, each at 0.6 μM, and incubated for 7 d. Compared to the control, Dif significantly suppressed N_2_O emissions from *P. janthinellum* SM-12f4X, with an inhibition of 64% (*F* = 18.45, *P* < 0.001~0.0027, Fig. 5a). Mez showed a lower inhibition efficiency of 24%. Both Mez and Dif induced significant NO accumulation during the incubation (*F* = 56.38~119.67, *P* < 0.001~0.015, Supplementary Fig. 12). These are consistent with the stronger P450Nor binding of Dif observed in the SPR and molecular-modeling analyzes and suggest that Dif’s high affinity to P450Nor and its effectiveness in obstructing HEM-Fe^3+^, thereby inhibiting fungal N_2_O emissions.

In the soil microcosms, Mez and Dif also increased soil NO accumulation (Supplementary Fig. 13), indicating that triazoles blocked NO reduction to N_2_O. Similarly, Dif exhibited a strong inhibition (77%), significantly higher than that of Mez (24%) at the concentration of 1 μM (*F* = 33.11, *P* < 0.001, Fig. 5b). When the bacterial inhibitor SM was added, Dif treatment achieved a remarkable 95% reduction in fungal N_2_O emissions compared to the control (*F* = 494.29, *P* < 0.001, Fig. 5c), without affecting bacterial and abiotic N_2_O emissions (*F* = 2.91~6.33, *P* = 0.066~0.163, Fig. 5d, e).

In the in-situ paddy field experiments, we employed a static chamber (60 cm × 60 cm) technique to quantify the mitigation of N_2_O emissions by Dif and Mez (0.05% of fertilizer N) while simultaneously applying urea fertilizers (Fig. 5f). Cumulative N_2_O emissions were monitored over 21 d (Fig. 5g), consistent with the pure-cultural and soil microcosm experiments, both Mez and Dif remarkably reduced field-scale N_2_O emissions, by 33% and 54% (*F* = 65.83, *P* < 0.001, Fig. 5h), respectively, with the inhibition of Dif greater than Mez.

We further conducted metagenomics and metaproteomics to validate Dif effects on the expression of denitrifying genes and its underlying detoxification mechanism. A total of 97 medium- and high-quality MAGs were recovered from Dif treatment (Supplementary Fig. 14), spanning 11 bacterial phyla and dominated by *Pseudomonadota* and *Bacteroidota*, which contained the *nirK* and *nosZ* genes, respectively (Supplementary Figs. 14 and 15). Similar to Mez, Dif also increased NosZ expression levels in each MAG (*P* < 0.001, Supplementary Data 1). The most abundant MAG (MAG135) under Dif treatment contained the complete denitrification pathway and a comprehensive triazole RD system. Abundant *nosZ*-harboring denitrifiers were also enriched in Dif-treated soils (Supplementary Fig. 16a). These denitrifiers showed higher enrichment in benzoate degradation pathways compared to the control (Supplementary Fig. 16b). Dif treatment was also associated with higher protein expression of pathways related to lipid metabolism, xenobiotic biodegradation and metabolism, carbon source utilization and energy efficiency in bacterial denitrifiers (*P* < 0.001, Supplementary Fig. 16c), consistent with the metabolic responses observed under Mez exposure.

### Estimation of ecological risk of Dif

To assess the potential ecological risk of Dif at the applied dose (0.02 mg/kg) in our field experiments, we conducted a global meta-analysis, compiling data from 143 studies on the toxic effects of Dif on aquatic and terrestrial species, as well as crops (Supplementary Fig. 17, and Supplementary Data 1). Reported residual Dif concentrations across environmental matrices ranged from 0.06 to 1.56 mg/kg (Supplementary Fig. 18a), with the lowest concentrations observed in sediments and the highest in animals. We then estimated predicted no-effect concentration (PNEC) for Dif by fitting dose-response curves to toxicity endpoints. In aquatic ecosystems, the PNEC for zebrafish embryo hatching rate was 0.75 mg/kg (Supplementary Fig. 18b). For terrestrial organisms, the PNECs for bee and earthworm survival were 1.0 and 0.3 mg/kg, respectively (Supplementary Fig. 18c, d). All of these values exceeded the Dif application dose used in our field experiment. Similarly, the PNECs for root length inhibition in soybean (1.2 mg/kg), sesbania (3.7 mg/kg, Supplementary Fig. 18e), wheat (1.3 mg/kg), and garlic (4.4 mg/kg, Supplementary Fig. 18f) were also higher than our applied dose. In addition, at the tested doses in this study, neither Mez nor Dif increased the abundances of soil resistance genes (Supplementary Fig. 19), indicating that their application did not promote resistome enrichment. These findings suggest that the ecological risk associated with the applied Dif dose in our study is minimal, although further site-specific and long-term assessments are needed to fully characterize ecological risk.

## Discussion

Agricultural soils are a major source of global anthropogenic N_2_O emissions^36^, which are projected to continue increasing, making climate change mitigation more challenging^37,38^. Seeking mitigation strategies for soil N_2_O emissions is thus crucial for both global climate mitigation and sustainable agriculture. Nitrification inhibitors, such as DMPP and DCD, have been widely applied in agricultural soils^13,14^, and are reported as effective tools, lowering N_2_O emissions by 20–47% at their highest dose (0.8–2% of N fertilizer) in upland agricultural soils^14,39^. By contrast, inhibitors specifically targeting denitrification-derived N_2_O emissions remained limited, despite denitrification contributing an estimated 30–70% of soil total N_2_O emissions^8,40^, and being particularly important in flooded paddy soils^41^. Recent advances in structural biology have resolved the architecture of key nitrogen-cycle enzymes^42,43^, but this knowledge has not been leveraged to develop inhibitors that precisely regulate microbial nitrogen transformations. Here, we address this gap by identifying fungal P450Nor as a tractable enzymatic target and demonstrating the potential of triazole-based inhibitors to reduce denitrification-derived N_2_O emissions.

Our structure-guided analysis and assays suggest that triazole compounds, including Mez and Dif, likely serve as potential inhibitors of soil N_2_O emissions by blocking fungal N_2_O production and promoting bacterial N_2_O reduction (Fig. 1). At a field application rate of 0.05% of fertilizer N, which is 16–40-fold lower than the nitrification inhibitor dose, these triazole compounds, particularly Dif, reduced N_2_O emissions by 54%, with the possibility for greater inhibition at higher doses (Fig. 5h). This highlights a promising option of triazoles for mitigating agricultural denitrification-derived N_2_O emissions.

Fungal denitrification in agricultural soils, especially in acidic or flooded fields, can produce significant amounts of N_2_O emissions^44^. Over recent decades, fungal denitrification has come under increasing scrutiny, primarily due to the lack of N_2_O reductase in denitrifying fungi. This causes their denitrification process to culminate in N_2_O release^45^, marking them as key N_2_O emitters in agricultural ecosystems^41,46^. Our previous study has indicated that fungal denitrification contributes to 51-63% of the total N_2_O emissions across national paddy soils in China^41^. Fungal denitrification has also been reported to account for more than 70% of N_2_O emissions in acidic upland tea-plantation soils^47,48^. Our study further substantiates the predominance of soil fungal N_2_O emissions (Fig. 1). Together, these findings support the view that fungal denitrification is a relevant target for N_2_O mitigation in agricultural ecosystems, regardless of flooded or upland conditions. These denitrifying fungi, adapted to oxic and anoxic conditions, uniquely integrate denitrification into mitochondrial respiration, a process observed across diverse ecosystems, including terrestrial and coastal regions^49^. Thus, the suppression of fungal denitrification could be beneficial in controlling N_2_O emissions, highlighting the potential of our findings to contribute to more sustainable agricultural practices.

P450Nor, a fungal cytochrome P450 enzyme functioning as nitric oxide reductase, catalyzes the reduction of NO to N_2_O and is central to fungal denitrification^21^. Our molecular-modeling and binding analysis suggest triazoles can bind to the HEM pocket of P450Nor, disrupting key proton delivery pathways involving amino residues A239, N246, S286, D393, and V394 (Fig. 3d). Specifically, triazoles form an H-bond with N246, impeding NAD(P)H binding. Guided by this structural model, we identified Dif as a more potent inhibitor directly occupying the HEM-Fe^3+^ and confirmed its effectiveness across in vitro, cellular, and environmental contexts. These results support P450Nor as a tractable molecular target for the development of fungal-denitrification inhibitors. This structure-guided approach is conceptually aligned with inhibitor-design strategies used in cancer, antibiotic, and antiviral research^50^. The P450Nor features a cys-coordinated HEM at its active site^21^, and its catalytic mechanism has been extensively studied through methods such as infrared spectroscopy, X-ray free-electron laser crystallography, and quantum mechanics/molecular mechanics calculations^24,25^. These comprehensive insights into P450Nor’s function and structure bolster its viability as a strategic target for developing fungal denitrification inhibitors. Despite having a good understanding of P450Nor’s catalytic mechanism, the intricacies of its assembly machinery remain elusive. This poses a challenge for identifying novel, more selective, and efficient targets for hindering fungal denitrification.

In addition to inhibiting fungal denitrification, we also find that triazoles could slightly promote bacterial N_2_O reduction (Figs. 1 and 4). Biological N_2_O reduction is largely restricted to *nosZ*-harboring bacteria, rendering them the primary known sink of N_2_O on Earth^18^. Studies have shown a negative correlation between the abundance of *nosZ* gene and N_2_O emissions in paddy fields^51^, estuarine sediments^52^, and wetlands^53^. In the Mez-treated soils, we observe an increase in *nosZ*-harboring denitrifiers (Fig. 4a). Our MAG-resolved analyzes further suggested that bacterial denitrifiers under triazole exposure encoded a putative resistance-detoxification (RD) system, which encompasses pathways for LPS synthesis, ABC transporters, GST detoxification, and benzoate degradation (Fig. 4b). The LPS synthesis pathway and ABC transporters serve a dual function, limiting Mez penetration into cells and reducing intracellular triazole concentrations. Furthermore, intracellular triazoles can be metabolized to benzoate by GST detoxification enzymes and subsequently degraded to acetyl-CoA via the benzoate degradation pathway, which is coupled with the TCA cycle, oxidative phosphorylation, and denitrification processes. These bacteria appear to have evolved an energy-efficient strategy to overcome the high activation energy barrier of N_2_O reduction^54^. Simultaneously, they demonstrate resilience to triazole toxicity, which aligns with the observed stable, low-level bacterial N_2_O emissions (Fig. 1d). Our results underscore the induced shifts by triazoles in the ecological niches of bacterial denitrifiers, especially those with or without the *nosZ* gene, thereby having differential effects on N_2_O emissions.

Triazoles are widely used as agricultural fungicides and represent a major class of crop-protection chemicals. However, their increasing use has raised concerns about the potential environmental risks^55^. In agricultural practices, Dif is often applied at 5 mg/kg^56^, with environmental residues reaching up to 0.06–1.56 mg/kg (Supplementary Fig. 18a). In this study, we used a 250-fold lower concentration of 0.02 mg/kg in the field experiments, thus resulting in lower environmental residues. Our meta-analysis showed that the PNEC values for Dif on aquatic and terrestrial species, as well as crops, are far higher than our applied dose (Supplementary Fig. 18), suggesting a lower risk. In addition, Dif degrades rapidly, with half-lives (DT_50_) ranging from 13.7 to 33.8 h in water and from 10.3 to 21.2 d in soil^57^. Its metabolites are also substantially less toxic than the parent compound^58^. These observations suggest that the low Dif dose used here is unlikely to exceed the estimated no-effect thresholds for the evaluated endpoints. However, ecological risk depends on exposure route, environmental persistence, repeated application, soil properties and non-target organisms. Therefore, long-term and site-specific risk assessments are needed before field-scale use of Dif or related triazole for N_2_O mitigation can be recommended.

**Fig. 6:**
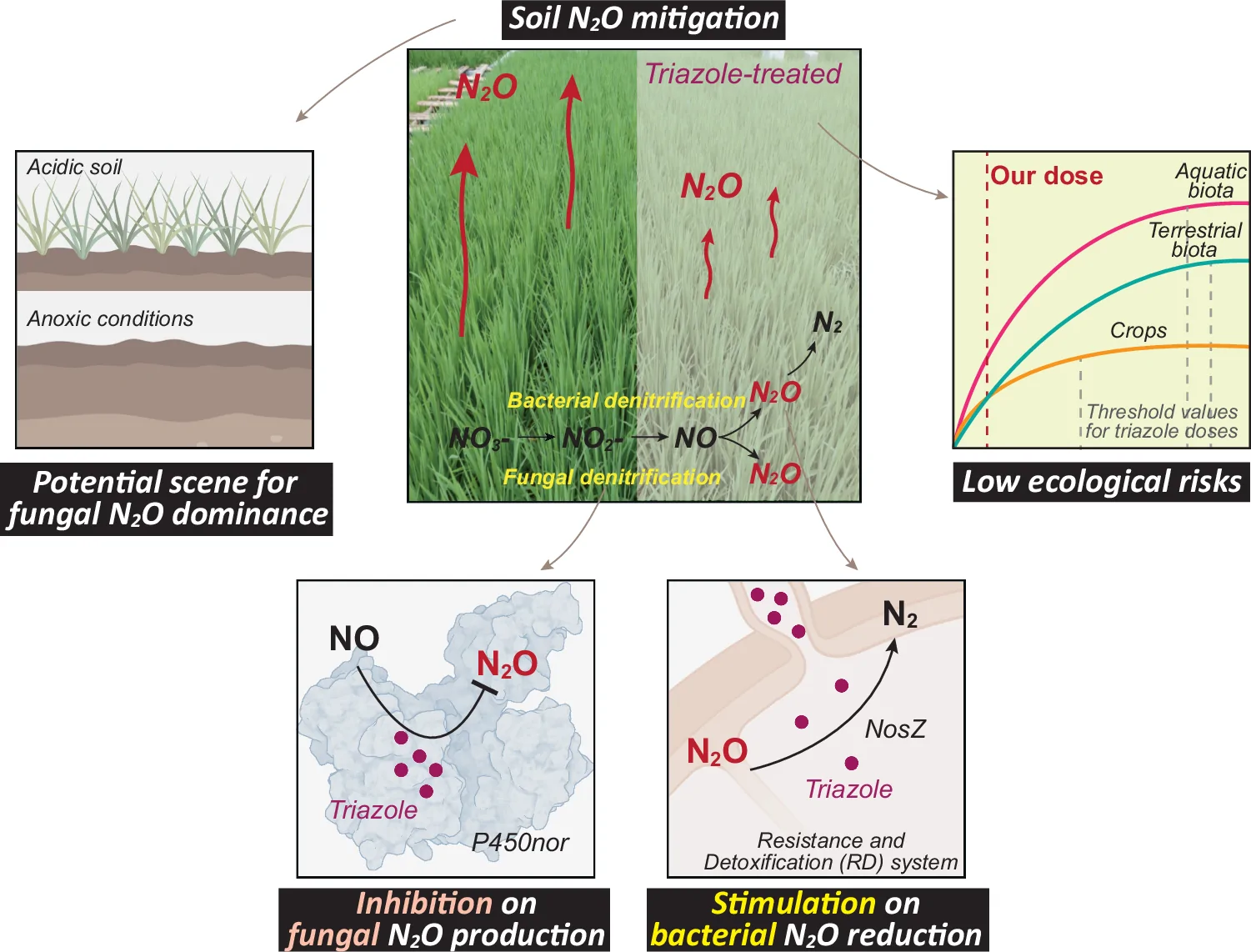
The potential of triazoles as inhibitors of soil denitrification-derived N_2_O emissions. Triazoles can simultaneously block fungal N_2_O production and stimulate bacterial N_2_O reduction at a low concentration (0.02 mg/kg), posing minimal ecosystem risk. This makes them particularly suitable for environments with fungal N_2_O dominance, such as tea plantation soils etc.

In summary, the mitigation of N_2_O emissions by triazoles demonstrated here provides compelling evidence that inhibiting P450Nor catalytic activity effectively suppresses fungal N_2_O emissions (Fig. 6). Crucially, the enhanced inhibition observed with Dif treatment suggests that targeting P450Nor HEM-Fe^3+^ offers a promising avenue for developing highly selective fungal denitrification inhibitors. Our results offer a proof-of-concept for utilizing triazoles, commonly employed as fungicides in agriculture, not only to safeguard crops by inhibiting fungal pathogens but also as potential P450Nor inhibitors to regulate soil N_2_O emissions. Notably, this approach requires substantially lower application doses than nitrification inhibitors, particularly in environments where fungal denitrification is predominant, addressing a current research gap.

Considering global N_2_O emissions from denitrification ranging from 1.3 to 3.1 Tg N_2_O-N yr^-1^ between 2007 and 2016^8^, it is a prime prospect that the application of triazoles might potentially reduce emissions significantly on a global scale. However, translating the present findings to regional or global scales will require multi-site field trials across soil types, crop systems, water regimes and fertilizer practices. Our study bridges nitrogen biogeochemistry with molecular and structural biology and reveals mechanisms for reducing denitrification-derived N_2_O emissions, providing insight into the development of denitrification inhibitors. Stemming from these findings, we anticipate that future development and applications of triazoles will optimize nitrogen use and reduce N_2_O emissions in agricultural systems. This, in turn, supports the long-term health of ecosystems and contributes to the global goals of achieving a more sustainable and resilient food production system.

## Methods

### Soil sampling and chemical preparation

Soil samples (surface soil 0–20 cm) were collected from a paddy field in the National Purple Soil Fertility and Fertilizer Benefit Monitoring Base of Southwest University, Beibei District, Chongqing, China (29°48’36” N, 106°24’33” E) in June 2022. The field had no history of triazole pesticide application. Four soil cores were collected. Before experiments, the soils were air-dried, sieved with a 2-mm mesh, and stored at 4 °C until the commencement of the experiments. The organic matter (SOM), total nitrogen (TN), and total phosphorus (TP) of soil samples were 17.47 ± 2.90, 1.17 ± 0.02, and 0.72 ± 0.02 g/kg, respectively. The pH of soil samples was 6.83 ± 0.10. Metconazole (Mez) and difenoconazole (Dif) with 99% purity were purchased from Sigma-Aldrich^®^.

### The 28-d experiments

Mez, a typical triazole compound, was selected to study its impacts on soil denitrification and N_2_O emission. A total of two treatments were set up, including a control (without Mez application) and Mez treatment (1 mg/kg Mez, environmental concentration). The experiments were set up in quadruplicate. Briefly, 50 g of dry soil was transferred into a 150 mL serum bottle, followed by the addition of glucose (1 mM) and KNO_3_ (1 mM). Mez was dissolved in acetone to produce a concentration of 1000 mg/L stock solution, after which it was diluted with ultrapure water and then added to the soil to attain 1 mg Mez per 1 kg of soil. For the control, an equivalent amount of pure water was added. All the serum bottles were purged with He (99.8%) for 5 min to achieve anoxic conditions. Subsequently, these bottles were anaerobically incubated at 25 °C in the dark for 28 d with 70% water-filled pore space (WFPS), a moisture level favorable for denitrification in moist agricultural soils. During incubation, soil samples were collected at 0, 4, 7, 14, and 28 d for determination of NO_3_^-^, NO_2_^-^, NH_4_^+^, and N_2_O concentrations. At the end of the experiment, soil DNA and proteins were extracted for subsequent sequencing.

### The 7-d experiments

To further explore the impacts of Mez on soil bacterial and fungal denitrification, five inhibitor treatments were established^59^, including (i) a control; (ii) Mez treatment; (iii) bacterial inhibitor treatments (streptomycin (SM), Mez+SM); (iv) fungal inhibitor treatments (cycloheximide (CHX), Mez+CHX); (v) combined bacterial and fungal inhibitor treatments (SM + CHX, Mez+SM + CHX). Each treatment was set up in quadruplicate. Briefly, 1 g of dry soil was put into a 12 ml Labco tube, followed by the addition of glucose (1 mM) and KNO_3_ (1 mM). Mez was dissolved in acetone to produce a concentration of 1000 mg/L stock solution, after which it was added to the soil samples to attain 1 mg Mez per 1 kg of soil. SM and CHX were dissolved in pure water and then added to the soil to attain a concentration of 6 and 10 mg/kg, respectively. An equivalent amount of pure water was added to the control. All the tubes were purged with He gas for 5 min and subjected to anaerobic incubation at 25°C in the dark for 7 d with 70% WFPS. During the incubation, NO_3_^-^, NO_2_^-^, NH_4_^+^, NO, and N_2_O concentrations were measured.

### Soil Mez extraction and analysis

The soil residual level of Mez was determined by analyzing soil samples at 2 h, 1, 3, 5, 7, 14, and 28 d after the addition of Mez in soils. The soil samples were extracted using the QuECHERS method^60^ and analyzed by a Nexera UC SFC system (Shimadzu, Kyoto, Japan) with LCMS/MS-8050 (Shimadzu, Kyoto, Japan). The extraction procedures and analysis are detailed in Supplementary methods.

The degradation kinetics were estimated using the first-order kinetics Eq. (1). The degradation half-life (*t*_1/2_) was calculated using Eq. (2):

1 $${{\mbox{C}}}_{({\mbox{t}})}={{\mbox{C}}}_{({\mbox{t}}=0)}\times \exp (-{{\mbox{k}}}_{{\mbox{h}}}\times {\mbox{t}})$$

2 $${{\mbox{t}}}_{1/2}={\mathrm{ln}}\left(2\right)/{{\mbox{k}}}_{{\mbox{h}}}$$

Where *C*_(*t*)_ and *k*_*h*_ represent the test compound concentrations (mg/kg) at time *t* and the degradation rate constant (d^-1^), respectively.

### Soil nitrogen measurements

Soil NO_3_^-^, NO_2_^-^, and NH_4_^+^ were extracted and analyzed by Ion Chromatograph (ICS-3000, Dionex, USA)^59^. We employed a destructive sampling method and extracted 10 mL of headspace gas using a syringe before opening the bottles. Gas samples were collected in the vacuum gas-tight vials containing NaOH to remove CO_2_^59^. The NO concentration was measured by a gas chromatograph with a chemiluminescent analyzer (42i NO-NO_2_-NO_X_, Thermo, USA). The N_2_O concentration was measured by a gas chromatograph equipped with an electron capture detector (GC-ECD, Agilent 7890, USA).

### DNA extraction and metagenomic sequencing

Soil microbial DNA was extracted from a 0.5 g fresh sample using the E.Z.N.A. soil DNA kit (Omega Bio-tek, Inc., USA) following the manufacturer’s instructions. The concentration and quality of DNA were analyzed by a NanoDrop Spectrophotometer (2000c Thermo Scientific, USA). Metagenomic shotgun sequencing of the soil microbial DNA was done on a HiSeq X-ten Platform (Illumina Inc.), generating 2×150 bp paired-end reads, which were processed using Fastp (version 0.20.0)^61^ to eliminate low-quality sequences and reads containing ambiguous N bases. Other details are provided in Supplementary methods.

### Metagenomic assembly, binning, and annotation

The paired sequence reads were assembled using MEGAHIT^62^, followed by the removal of short contigs (<1000 bp) from the assemblies^63^. ORF prediction and amino acid translation were done using Prodigal based on Kmer overlap and De Bruijn graphs from contigs >800 bp. The potential functions of ORFs were identified by searching the Kyoto Encyclopedia of Genes and Genomes (KEGG) orthologs (KOs) using DIAMOND. Metagenome binning was performed using vamb. The quality of the MAGs was evaluated using CheckM v1.2.0^64^, and the MAGs were then classified taxonomically using GTDB-tK^65^. All the MAGs were then combined and dereplicated at 95% average nucleotide identity (ANI) using dRep. Other details are provided in Supplementary methods.

### Total protein extraction and digestion

Soil proteins were extracted and processed according to previous studies^66^. Frozen soil samples were homogenized in BPP buffer using a high-flux tissue grinder and centrifuged at 4 °C. The supernatant was mixed with Tris-saturated phenol, followed by phase separation and protein precipitation with ammonium acetate methanol. After washing with acetone, proteins were solubilized in protein lysate containing 8 M urea and 1% SDS, then quantified by the BCA Protein Assay Kit (Thermo, USA) following the manufacturer’s instructions. For digestion, 100 μg of protein was reduced with TCEP and alkylated with IAM, followed by trypsin digestion overnight at a 1:50 ratio. The resulting peptides were desalted, dried, and quantified by UV absorption on NANODROP ONE (Thermo, USA).

### Mass spectrometry and data analysis

Peptides were analyzed on a VanquishNeo system coupled with an Orbitrap Astral mass spectrometer (Thermo, USA) at Majorbio Bio-Pharm Technology Co. Ltd (Shanghai, China). Data were acquired over 70–1050 *m/z* (MS1) and 150-2000 *m/z* (MS2). Protein identification was performed using Spectronaut software^67^ with strict parameters (FDR ≤ 0.01, peptide confidence ≥99%). Differentially expressed proteins (DEPs) were identified by t-test with thresholds of fold change >1.2 or <0.83 and *P* < 0.05. Functional annotation and enrichment analysis were conducted using GO (http://geneontology.org/) and KEGG databases. Proteins affiliated with each MAG were analyzed for metabolic pathways. The details are provided in Supplementary methods.

### Molecular docking and molecular dynamics simulation

The computational simulation was carried out in the Maestro graphical user interface of Schrödinger (www.schrodinger.com) on a desktop workstation with the Ubuntu platform. The details of the ligands and protein preparation are provided in the Supplementary methods. The bacterial NirK and fungal P450Nor static X-ray structures^25^ were gained from the Protein Data Bank (PDB code: 6ZAS and 7DVO). The fungal NirK structure was obtained by using SWISS-MODEL homology modeling based on the structure of the protein with PDB code 6THE. The files of 2D SDF structures of Mez and Dif were gained from the PubChem database, following processing by the Ligprep module in Schrödinger and generating their respective 3D chiral conformations^68^. The Glide module was used for molecular docking, while the binding sites were identified using the Receptor Grid Generation module^69^.

The molecular dynamics (MD) were performed by the Desmond module of Schrödinger. A water-soaked solvent system was chosen for MD simulation. The best conformations of Mez and Dif obtained from the docking results were loaded into the Desmond module and subsequently optimized and minimized using the OPLS_3e force field^70^. The details for the simulation are provided in the Supplementary methods. A 100 ns simulation was run, during which 1000 frames were saved to the trajectory. MD simulation trajectory was analyzed using the Simulation Interaction Diagram.

### Surface plasmon resonance (SPR) experiments

We used the pET28a-6His-MBP-ppase-P450Nor expression plasmid to express P450Nor from *Fusarium oxysporum* in *E. coli* BL21 Gold (DE3) (Supplementary Fig. 20) and purified. The details are provided in Supplementary methods^21^.

The SPR experiments were conducted on Biacore-8K (GE-Healthcare, Chicago, IL, USA). A total of 20 μg/mL of recombinant protein P450Nor was immobilized onto a Series S Sensor Chip CM5 (GE Healthcare) with Amine Coupling Kit (GE Healthcare), maintaining a pH of 4.5, with a flow rate of 10 μL/min and an immobilization level of 4000 Resonance Units (RU).

The interaction between P450Nor and the ligands Mez/Dif was evaluated under a constant flow of PBS (pH 7.4), supplemented with 0.05% Tween-20 and 5% DMSO, at a temperature of 25 °C. The flow rate was set to 30 μL/min, with an association phase of 120 s and a dissociation phase of equal duration. We prepared twofold serial dilutions of Mez, with concentrations ranging from 400 μM to 78.125 nM. Similarly, Dif dilutions ranged from 200 μM to 390.625 nM. The accuracy of binding data was ensured by applying double referencing, which included subtraction of both blank cycle and reference flow cell data. The processed data were then fitted to a 1:1 Langmuir binding model using the Biacore Insight Evaluation Software version 4.0 (GE Healthcare).

### Validation of Dif inhibition on N_2_O emission

To validate the Dif-mediated inhibition of N_2_O emission, especially the fungal source, pure-culture assays, soil microcosm incubations with the addition of bacterial and/or fungal inhibitors, and in-situ paddy field experiments were performed. For pure-culture assays, the previously isolated denitrifying fungal strain *Penicillium janthinellum* SM-12f4^59^ was used. Three experimental setups were established, including a control (without Mez or Dif application), a Mez (0.6 μM), and a Dif (0.6 μM) treatment. In each treatment, a conidial suspension of *P. janthinellum* was inoculated into 50 mL of Potato Dextrose Broth (PDB). After 2 days of initial growth, sterile PDB supplemented with NaNO_2_ (to achieve a final concentration of 5 mM), Mez, or Dif was added to the respective cultures, followed by incubation at 29 °C. After a 7-d incubation, the headspace gas was collected for NO and N_2_O analyzes.

For soil microcosm experiments, three groups were set up, including a control (without Mez or Dif application), a Mez (1 μM) group, and a Dif (1 μM) group. This experimental setup mirrored the approach used in our 7-d experiment. Additionally, five supplementary bacterial and fungal inhibitor treatments were introduced. This methodology aligns with the protocols outlined in our 7-d experiment.

For paddy field experiments, three treatments, including a control (without Mez or Dif application), a Mez (0.05% of urea fertilizer, 5 μM, 0.016 mg/kg), and a Dif (0.05% of urea fertilizer, 5 μM, 0.02 mg/kg) treatment, were established. The in-situ measurements of N_2_O flux (mg/(h m^2^)) were conducted using the static chamber technique^71^. The chambers had a volume of ~27 L and an area of 0.09  m^2^, and were fitted with airtight lids, two sampling ports, and vent tubes to equalize pressure. Gas samples were collected from 0.5 to 504 h. To ensure air homogeneity within the chamber, a 200-mL syringe was used to flush and mix the chamber’s headspace air before sampling. A 10 mL gas sample was extracted and immediately sealed with Parafilm. Chamber temperature was also recorded.

### Data collection and meta-analysis for ecological risk assessment of Dif

We collected global data on residual concentrations of Dif and its toxicity to organisms to assess its potential ecological risks. An environmental concentration and toxicological database was compiled from published literature. This section presents a meta‑analysis of Dif concentrations in various environmental matrices based on studies published up to March 12, 2025. Relevant studies were retrieved from online databases including Web of Knowledge, Scopus, Google Scholar, and CNKI, using search keywords such as “difenoconazole” or “triazole” (Supplementary Fig. 17).

Residual levels of Dif and corresponding ecotoxicological data were extracted from relevant studies. These ecotoxicity data, derived from experimental studies, were used to evaluate environmental risks to ecosystems. Relevant articles were selected based on title and abstract screening, with irrelevant and duplicate publications excluded. After thorough screening, a total of 143 original research articles, encompassing 1686 observations, were included in this analysis. Data were extracted from tables and texts, or digitized from figures using GetData Graph Digitizer 2.22 (http://getdata-graph-digitizer.com/). Concentrations reported as “not detected” or “below detection limit” were recorded as zero.

### Statistical analyses

Data analyzes were carried out using SPSS version 20.0 (IBM Corp., Armonk, NY, USA), with data expressed as mean values ± standard deviation (SD). Differences between treatments were assessed through one-way ANOVA, followed by Tukey’s HSD post-hoc test for pairwise comparisons, with a significance level of *P* < 0.05. The normality of the data was evaluated using the Shapiro–Wilk test, and variance homogeneity was assessed using Levene’s test. When the data failed to meet parametric assumptions, the Kruskal–Wallis test was employed. Graphical visualizations were created using GraphPad Prism version 7.0 (GraphPad Software, San Diego, CA, USA) and Adobe Illustrator (Adobe Inc., USA).

### Reporting summary

Further information on research design is available in the Nature Portfolio Reporting Summary linked to this article.

## Supplementary information

Supplementary Information

Peer Review file

Description of Additional Supplementary Files

Supplementary Data 1

Supplementary Data 2

Reporting Summary

## Data Availability

Metagenome sequencing data have been deposited in the GSA, China National Center for Bioinformation with the accession number CRA045584 (https://ngdc.cncb.ac.cn/gsa/browse/CRA045584). Metaproteome data have been deposited in the OMIX, China National Center for Bioinformation with the accession number OMIX018172 (https://ngdc.cncb.ac.cn/omix/release/OMIX018172). The phylogenetic tree was visualized using iTOL (https://itol.embl.de). The docking results were visualized using Pymol (version 2.5.4). MAG information, MAG-centric NirK and NosZ expression, literature used for meta analyses, and the raw data used for plotting figures are provided as Supplementary Data 1 and 2.
