# Supplementary material for: Structure-guided identification of denitrification inhibitors to mitigate agricultural N2O emissions
Source: Nat Commun. 2026 Aug 20;17:9944. doi: 10.1038/s41467-026-76975-6 (PMC13586399; doi:10.1038/s41467-026-76975-6)
Supplement: Supplementary file 1 [file 41467_2026_76975_MOESM1_ESM.pdf]

Supplementary Information

Structure-guided identification of denitrification inhibitors to mitigate agricultural N_2O emissions

Yue Deng[1,2], Haixia Zeng[1,2], Lin Zhang[3], Bin Liu[2,4], Linfa Fang[1,2], Hailin Zhang[1], Yongliang Yang[5], Ran Xiao[1,2], Lijun Hou[6], Jinbo Zhang[7,8], Fusuo Zhang[1,4], Prakash Lakshmanan[1,9], Yingmu Wang[10], Yong-guan Zhu[11,12], Xiaoxuan Su[1,2,*], Xinping Chen [1,2,4,*]

Supplementary methods

Soil Mez extraction and analysis

The soil samples were weighed 5.00 ± 0.01 g into a 50 mL polypropylene centrifuge tube. After adding 5 mL acetonitrile, the tube was vortexed for 2 min to guarantee the complete sample-to-solvent interaction, then centrifuged at 10000 rpm for 5 min to get 1 mL of the supernatant. Before SFC-MS/MS analysis, the supernatant was filtered by a 0.22 μm polytetrafluoroethylene filter.

The concentration of Mez analysis was performed on the Nexera UC SFC system (Shimadzu, Kyoto, Japan) with an LCMS-8050 (Shimadzu, Kyoto, Japan). The column was CHIRALPARK AD-3. The column temperature, flow rate, and BRP pressure were set at 40℃, 2.5 mL/min, and 10 MPa, respectively. The injection volume was 1 μL. Mass spectrometry conditions were as follows: flow rate of atomizing gas (N_2) was 3 L/min, flow rates of heating gas (air) and drying gas (N_2) were 10 L/min, capillary temperature, desolvation line temperature, and heat block temperature were 300℃, 250℃ and 400℃, respectively. m/z 320.0 > 70.0 (−40 eV) and m/ z 320.0 > 125.05 (−37 eV) were used as confirmation and quantification ion pair, respectively.

High throughput sequencing

DNA was shared to 300 bp with the Covaris ultrasonic crusher. And to prepare sequencing library, the fragments were treated by end repair, a tailing, and ligation of Illumina compatible adapters. DNA sequencing libraries were deep sequenced on Illumina Novaseq PE150 platform at Allwegene Company (Beijing, China). After the run, image analysis, base calling and error estimation were performed using Illumina Analysis Pipeline Version 2.6.

Sequence quality control and genome assembly

The quality control of the raw data was carried out by using fastp[1], including the removal of adapter sequences and low-quality reads. Filter the reads if they were with the adapter sequence, N (uncertain base) ratio greater than 10 %, and the content of low-quality base ($Q \leq 10$) greater than 50 %.

These high-quality reads were then assembled to contigs using MEGAHIT[2], which makes use of succinct de Bruijin graphs. Contigs with the length being or over 800 bp were selected as the final assembling result.

Gene prediction, taxonomy, and function annotation

Openb reading frames (ORFs) in contigs were identified using Prodigal[3] (https://githu b.com/hyattpd/prodigal/wiki). The predicted ORFs with length being or over 100 bp were retrieved and translated into amino acid sequences using the NCBI translation table.

A non-redundant gene catalog was constructed using CD-HIT[4] (http://www.bioinformatics.o rg/cd-hit/, version 4.6.1) with 90% sequence identity and 90% coverage. Reads after quality control were mapped to the non-redundant gene catalog with 95% identity using bowtie 2[5] (http://bowtie-bio.sourceforge.net/bowtie2/index.shtml), and gene abundance in each sample was evaluated[6].

Representative sequences of non-redundant gene catalog were annotated based on the NCBI NR database (Version: 2021.11) using blastp as implemented in Diamond[7] (http://w ww.diamondsearch.org/index.php, version 0.8.35) with e-value cutoff of $1e^{-5}$ using Diamond for taxonomic annotations. Cluster of orthologous groups of proteins annotation for the representative sequences were performed using Diamond against egg NOG database (version 4.5.1) with an e-value cutoff of $1e^{-5}$. The KEGG annotation was conducted using Diamond against the Kyoto

Encyclopedia of Genes and Genomes database (http://www.genome.jp/keeg/, version 94.2) with an e-value cutoff of 1e^{-5}.

Binning analysis

Binning was performed with vamb[8] (https://github.com/RasmussenLab/vamb), and bins results were evaluated using checkm[9] to extract information on ID, Taxonomy, Completeness, and Contamination. The bins results were screened with the criteria of Completeness>50% and Contamination<10%. The species classification annotation and phylogenetic tree construction of bins were performed using GTDB[10] (https://gtdb.ecogenomic.org/). Salmon[11] was used to quantify the abundance of bins and estimate the abundance of each bin in the sample.

Quantitative metaproteomic

The acquire MS/MS spectra were analyzed by searching against the proteome of the 238 MAGs recovered from the metagenomics data, using MaxQuant version 1.6.3.3. Proteins were quantified was performed by the MaxLFQ algorithm in MaxQuant[12]. Variable modification included N-terminal acetylation and methionine oxidation, while carbamidomethylation o was used as a fixed modification. Peptide identification tolerance were set to 4.5 ppm and 20 ppm for MS and MS/MS, respectively, with allowance for two missed trypsin cleavages. Additional quality filtering and downstream interpretation were performed in the software platform Perseus version 1.6.0.7[13], including removal of contaminants, reversed sequences, and single-modified peptides. Protein identification were filtered to ensure a 1% false discovery rate (FDR) using the target-decoy approach.

Scanning the detracted proteins affiliated to each MAGs recovered from metagenomic data for enzymes involved in specific metabolic pathways to construct metaproteome-base metabolic maps[14]. The proteins size of individual MAGs was calculated in tow ways: either by the number of proteins detected per

MAGs or by summing the LFQ-values of all proteins belonging to each MAG, normalized to the total LFQ for the sample

Ligand and protein preparation method

Metconazole (Mez) and difenoconazole (Dif) structures were downloaded from Pubchem (https://pubchem.ncbi.nlm.nih.gov), then optimized using LigPre. The protein structure of Nirk and P450Nor with code 6ZAS and 7DVO, respectively, were downloaded from Protein Data Bank (https://www.rcsb.org). Fungal Nirk protein structure was gained using SWISS-MODEL as the template protein (PDB code: 6THE). The protein structure was optimized using Protein Prep Wizard (PPW) tool in Schrödinger software. The missing hydrogens, side chains, and residues were added, and waters were deleted. The correct ionization state was generated at pH 7.4, and the hydrogen bond network was regenerated, then the protein structure was minimized by PROPKA tool.

Molecular docking method

The binding site was identified by Sitemap module, then the enclosing box was set to including the binding site by Receptor Grid Generation module in Schrödinger for identify the active site of protein. Four complexes that would be modeled were obtained using extra precision of the docking mode. Meanwhile, multiple scoring functions such as Glid energy, Glide score, and Glide emodel were used to calculate and analyze the binding affinities.

Molecular dynamics (MD) simulation method

MD simulation was performed to produce the trajectory of the particles' coordinates, velocities, and energies to access the structural variation and to study the stability of various trajectory graphs by the Desmond module of Schrödinger. The OPLS4 force field was employed to parameterize the protein and small molecules, while the SPCE model was used for the water solvent. The protein-small

molecule complex was placed in a cubic water box and solvated. The system's charge was neutralized by adding 0.150 M chloride and sodium ions. The energy of the system was initially minimized using the steepest descent minimization method for 50,000 steps. Subsequently, the positions of heavy atoms were restrained for NVT and NPT equilibration for an additional 50,000 steps. The system temperature was maintained at 300 K, and the system pressure was maintained at 1 bar. After completing the two equilibration stages, an unrestricted simulation was performed for 100 ns. The interactions were analyzed and dynamic trajectory animations were generated using Maestro 2023.

Expression and purification of P450Nor

Competent BL21 Gold (DE3) *Escherichia coli* cells were transformed with P450Nor expression constructs, cultured at 37 ℃ to an optical density at 600 nm of between 0.6 and 0.8, and protein expression was induced with 0.1 mM IPTG 16 h at 18 ℃. Cells were collected by centrifugation at 4,000 rpm and 15 minutes at 4 ℃ (Beckman Coulter, J6-M1) and lysed in 50 mM Tris pH 8.0, 300mM NaCl, 10% glycerol, 1% CHAPS, 1mM TCEP, 10 μg/mL DNase I, 2 pills of protease inhibitor complete EDTA free (Roche), followed by using sonication (Ningbo Scientz Biotechnology, P.R. China) twice (worked 10 s, closed 10s, 10 minutes). Cell debris was clarified by centrifugation at 40,000g for 90 min at 4°C (Beckman Coulter, Avanti J-25). Supernatants containing the N-terminal His-tagged P450Nor proteins were filtered through a 0.45-μm membrane (295-3345, Nalgene), applied to a 5-ml prepacked HisTrap excel Ni^{2+} metal affinity chromatography column (17371206, GE Healthcare Life Sciences). Bound P450Nor proteins were eluted with a linear gradient of 18 column volumes of imidazole(0-0.25M). Fractions containing P450Nor proteins as determined by SDS-PAGE and were digested with HRV 3C Protease (Suzhou ReadCrystal Biotechnology, P.R. China) and dialyzed to 10mM

IMID overnight at 4 ° C. The samples were centrifuged at 12000rpm for 20min and the supernatant was loaded onto a 5-mL HiTrap Q HP anion exchange chromatography column (17115401, GE Healthcare Life Sciences) equilibrated in 20 mM Tris-HCl buffer, pH 8.0, 0,1mM TCEP. P450Nor proteins were eluted using a linear gradient of 13 column volumes of NaCl (0-1 M) in the same buffer. Fractions containing P450Nor proteins were pooled, concentrated to about 100 mg/mL and performed SEC in 20 mM Tris-HCl, pH 8.0, 150mM NaCl, 1mM TECP, 5% glycerol. All column chromatography purifications were performed on an AKTA Pure 25 system (GE Healthcare) at 4℃. Purified P450Nor proteins were flash frozen in small aliquots in liquid nitrogen and stored at -80°C until use. Protein concentrations were determined using a NanoDrop ND-1000 spectrophotometer (Nanodrop Technologies) at 280 nm.

Amnio acid sequence of pET28a-6His-MBP-ppase-P450Nor

MGSSHHHHHHSSGLVPRGSHMMKIEEGKLVIWINGDKGYNGLAEVGKKFEKDTGIK
VTVEHPDKLEEKFPQVAATGDGPDIIFWAHDRFGGYAQSGLLAEITPDKAFQDKLYPFTWD
AVRYNGKLIAYPIAVEALSLIYNKDLLPNPPKTWEEIPALDKELKAKGKSALMFNLQEPYFTW
PLIAADGGYAFKYENGKYDIKDVGVDNAGAKAGLTFLVDLIKNKHMNADTDYSIAEAAFNK
GETAMTINGPWAWSNIDTSKVNYGVTVLPTFKGQPSKPFVGVLSAGINAASPNKELAKEFL
ENYLLTDEGLEAVNKDKPLGAVALKSYEEELAKDPRIAATMENAQKGEIMPNIPQMSAFWY
AVRTAVINAASGRQTVDEALKDAETLEVLFQGPMASGAPSFPFSRASGPEPPAEFAKLRATN
PVSQVKLFDGSLAWLVTKHKDVCFVATSEKLSKVRTRQGFPELSASGKQAAKAKPTFVDM
DPPEHMHQRSMVEPTFTPEAVKNLQPYIQRTVDDLLEQMKQKGCANGPVDLVKEFALPVP
SYIIYTLLGVPFNDLEYLTQQNAIRTNGSSTAREASAANQELLDYLAILVEQRLVEPKDDIISKL
CTEQVKPGNIDKSDAVQIAFLLLVAGNATMVNMIALGVATLAQHPDQLAQLKANPSLAPQ
FVEELCRYHTASALAIKRTAKEDVMIGDKLVRANEGIIASNQSANRDEEVFENPDEFNMNR
KWPPQDPLGFGFGDHRCIAEHLAKAELTTVFSTLYQKFPDLKVVPLGKINYTPLNRDVGIVD

LPVIF

Base sequence of pET28a-6His-MBP-ppase-P450Nor

CCAAGGCTTCCCTGAGCTTAGCGCCAGTGGAAAGCAAGCAGCCAAGGCAAAGC
CGACATTTGTCGACATGGATCCCCCAGAGCACATGCATCAGAGGAGCATGGTGGAGC
CGACCTTTACCCCCGAAGCTGTCAAGAATTTGCAGCCTTACATCCAGAGGACTGTCGAT
GATCTACTGGAGCAGATGAAGCAGAAGGGGTGTGCCAATGGTCCAGTTGACCTTGTCA
AAGAGTTTGCTCTCCCTGTCCCCTCCTATATCATCTACACACTTCTCGGAGTTCCCTTCA
ATGATCTCGAATATCTTACGCAGCAGAACGCCATTCGGACAAATGGTAGCTCCACTGC
CCGAGAGGCCTCTGCCGCTAACCAGGAACTCCTTGATTACCTTGCAATTCTTGTCGAGC
AGCGTCTCGTAGAGCCAAAGGATGATATCATCAGCAAGCTCTGCACTGAGCAAGTCAA
GCCTGGAAATATCGACAAGTCCGATGCTGTCCAGATTGCTTTCTTGCTCCTTGTCGCTG
GCAACGCAACCATGGTAAACATGATTGCTCTGGGCGTCGCCACTCTGGCTCAGCACCC
TGATCAGCTGGCGCAACTCAAGGCCAACCCATCCCTTGCGCCCCAGTTTGTCGAGGAA
CTCTGTCGCTACCATACTGCTTCTGCACTAGCTATCAAGCGTACTGCCAAGGAGGATGT
CATGATCGGTGACAAGCTGGTCCGGGCGAATGAAGGCATTATCGCATCCAACCAGTCA
GCCAACAGAGATGAAGAAGTCTTCGAGAATCCCGATGAGTTCAACATGAACCGCAAG
TGGCCTCCTCAAGATCCTCTTGGCTTTGGCTTTGGTGATCACAGATGTATCGCTGAGCA
TCTTGCAAAGGCTGAACTCACAACTGTGTTCTCAACACTGTATCAGAAGTTCCCAGATC
TCAAGGTTGCGGTTCCATTGGGAAAGATCAACTACACGCCTCTGAACCGAGATGTCGG
AATCGTGGATCTTCCTGTCATTTTT

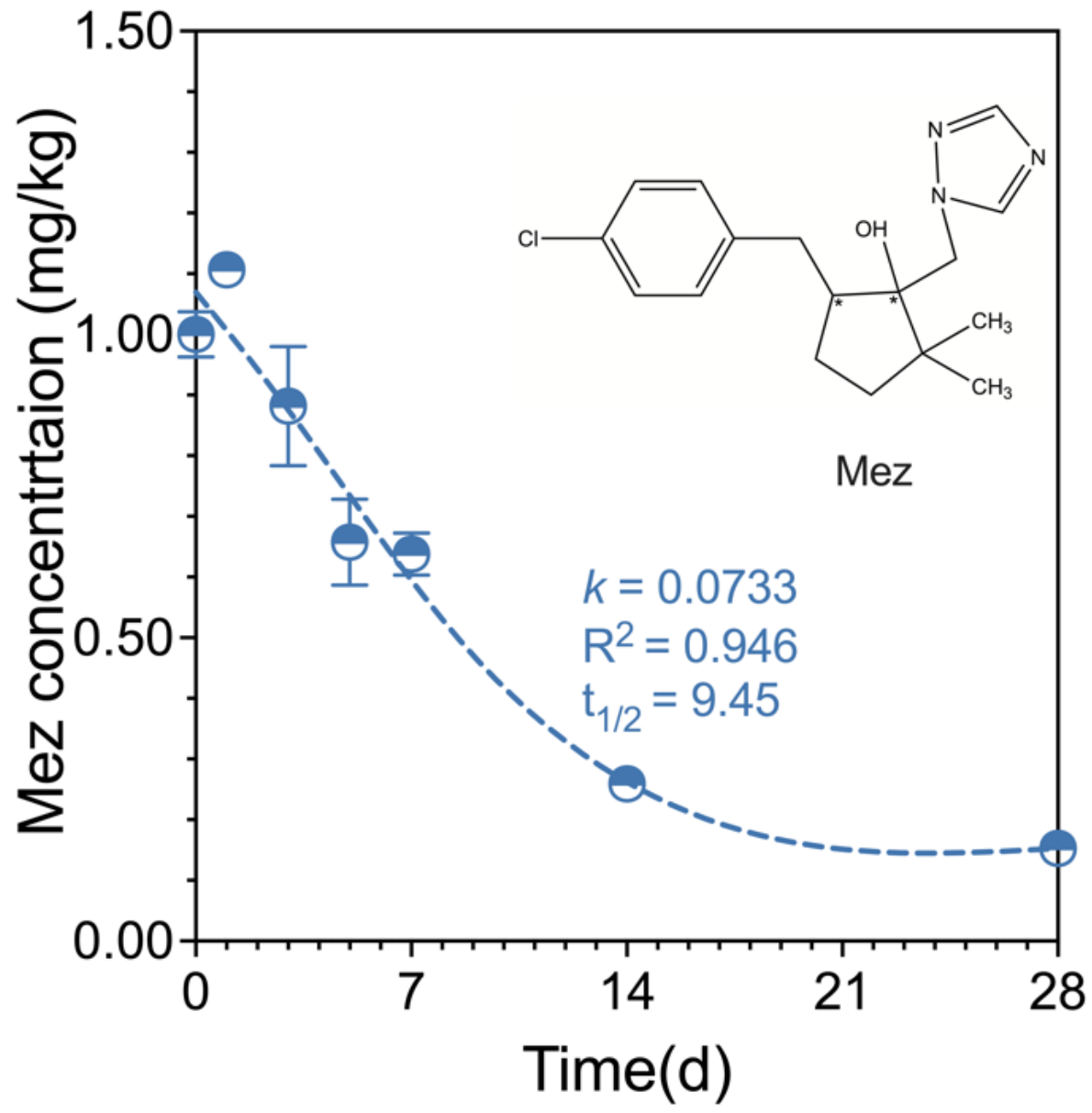

Supplementary Fig. 1: Mez residual concentrations and dissipation kinetics in soil samples during the incubation. The concentrations of Mez were measured at 2 h, 1, 3, 5, 7, 14, and 28 d, respectively. The structure of Mez and dissipation kinetic parameters are shown in the figure. Data are presented as mean ± SD (biological replicate, n=4).

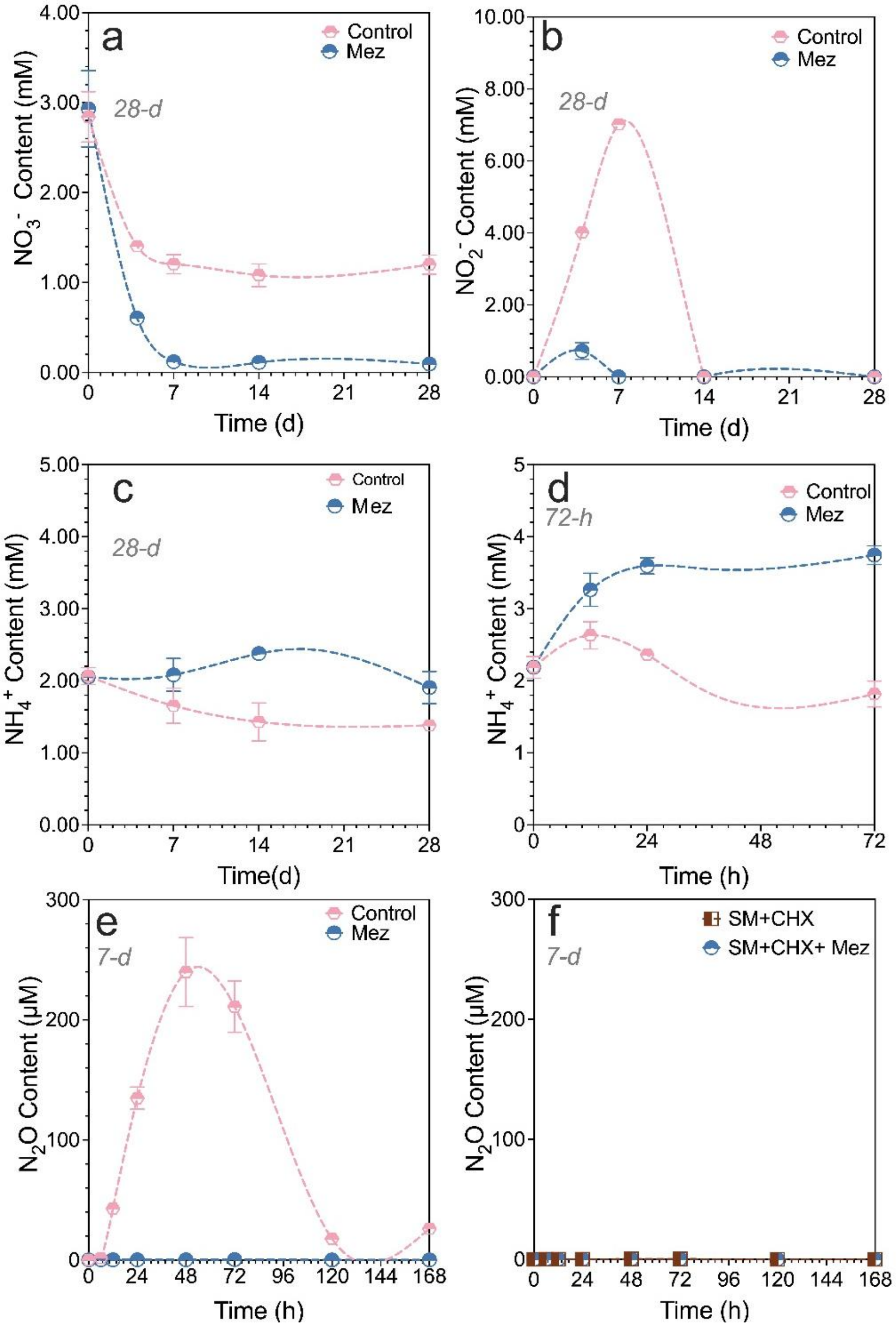

Supplementary Fig. 2: Variations of soil nitrogen speciation (NO_3^-, NO_2^-, NH_4^+, and N_2O) in response to Mez. (a), **(b)**, and **(c)** Changes in the concentrations of NO_3^-, NO_2^-, and NH_4^+ along with time during a 28-d incubation. **(d)** Changes in the concentrations of NH_4^+ along with time during a 72-h incubation. **(e)** and **(f)** Changes in the concentrations of N_2O along with time during a 7-d incubation. Data are presented as mean ± SD (biological replicate, n=4).

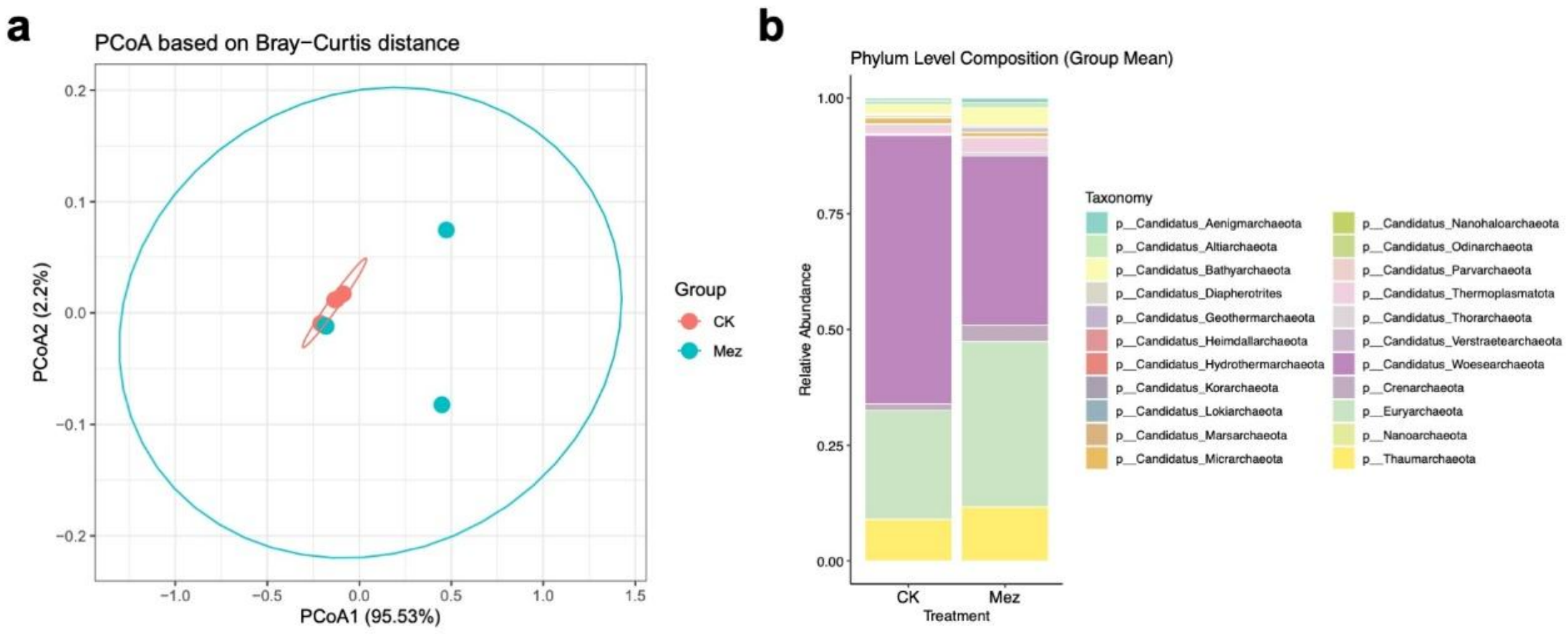

Supplementary Fig. 3: Soil archaeal community in response to Mez. (a) Principal coordinate analysis (PCoA) based on Bray–Curtis distance showing the archaeal community structure in the control (CK) and metconazole (Mez) treatments. Each point represents an individual biological replicate. **(b)** Relative abundances of archaeal taxa at the phylum level in the CK and Mez treatments. Data are presented as the mean of four independent biological replicate.

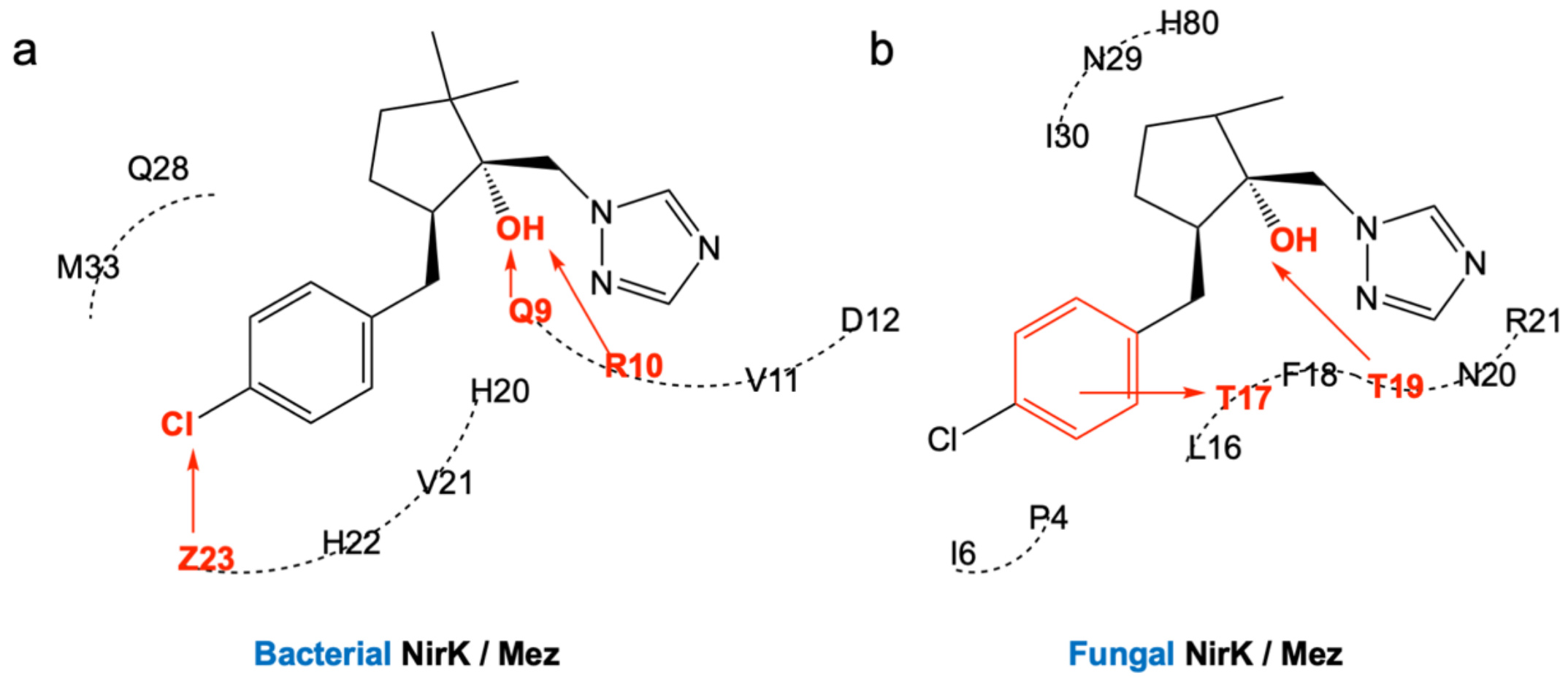

Supplementary Fig. 4: Chemical structure of Mez with predicted binding mode. **(a)** Mez forms H bound with Q9 and R10 resides and forms Halogen bound with Z23 residue within bacterial NirK. **(b)** Mez forms H bound with T19 and forms π-π stacking interaction with T17 within fungal NirK.

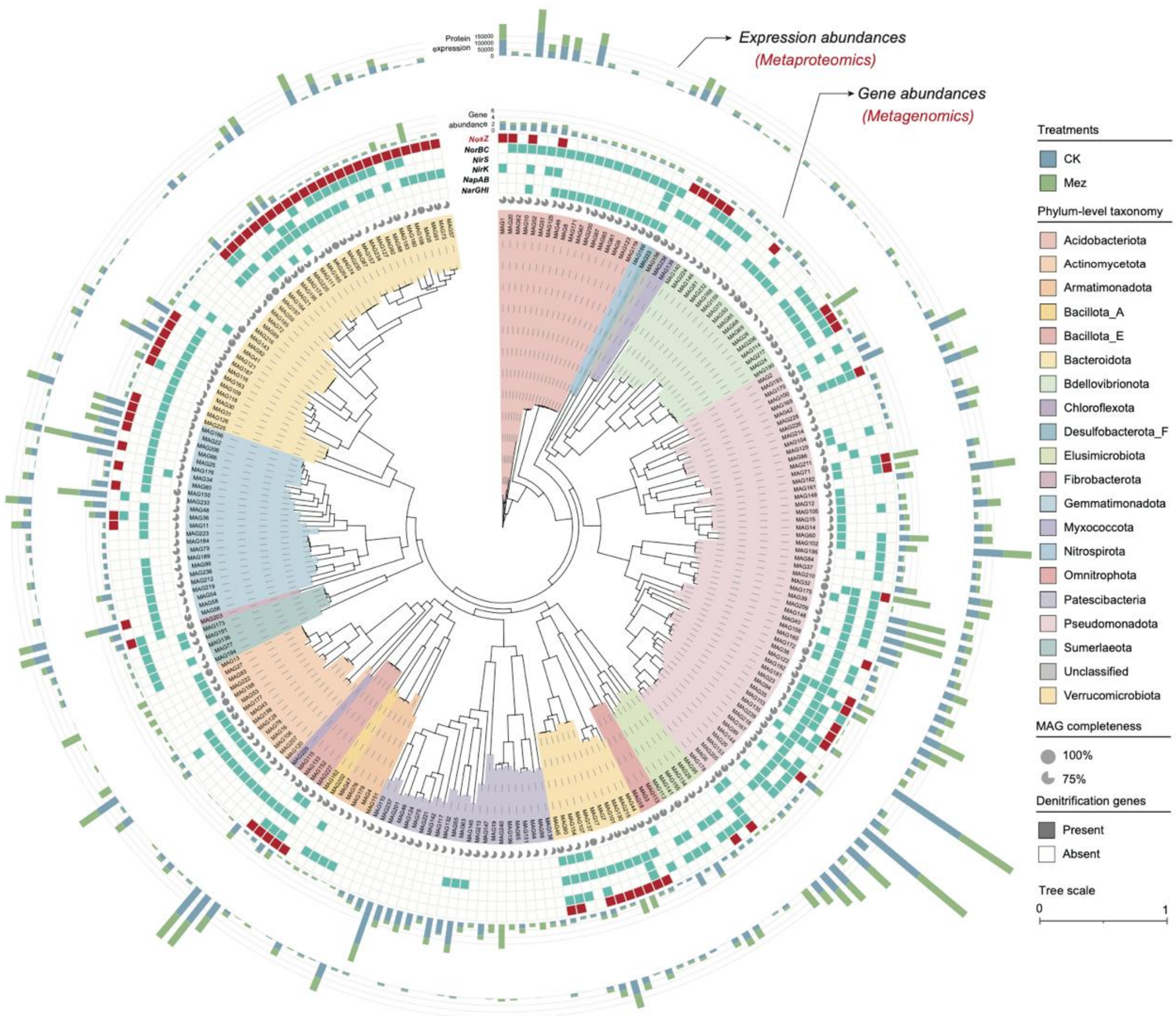

Supplementary Fig. 5: Maximum likelihood-based phylogenetic analysis of dereplicated 238 medium- and high-quality MAGs in response to Mez. Taxonomic classification of the MAGs was inferred using the Genome Taxonomy Database (GTDB) and is displayed at the phylum level by label and branch coloring. The bar chart shows the abundances (RPKM) of MAGs in control and Mez treatments at the gene (metagenomics) and expression (metaproteomics) levels. The heatmap indicates the MAGs genomes whether contain the *nosZ* gene (maroon) or other denitrification genes (cyan). The completeness and contamination of each MAG is represented by the pies at the branch tips.

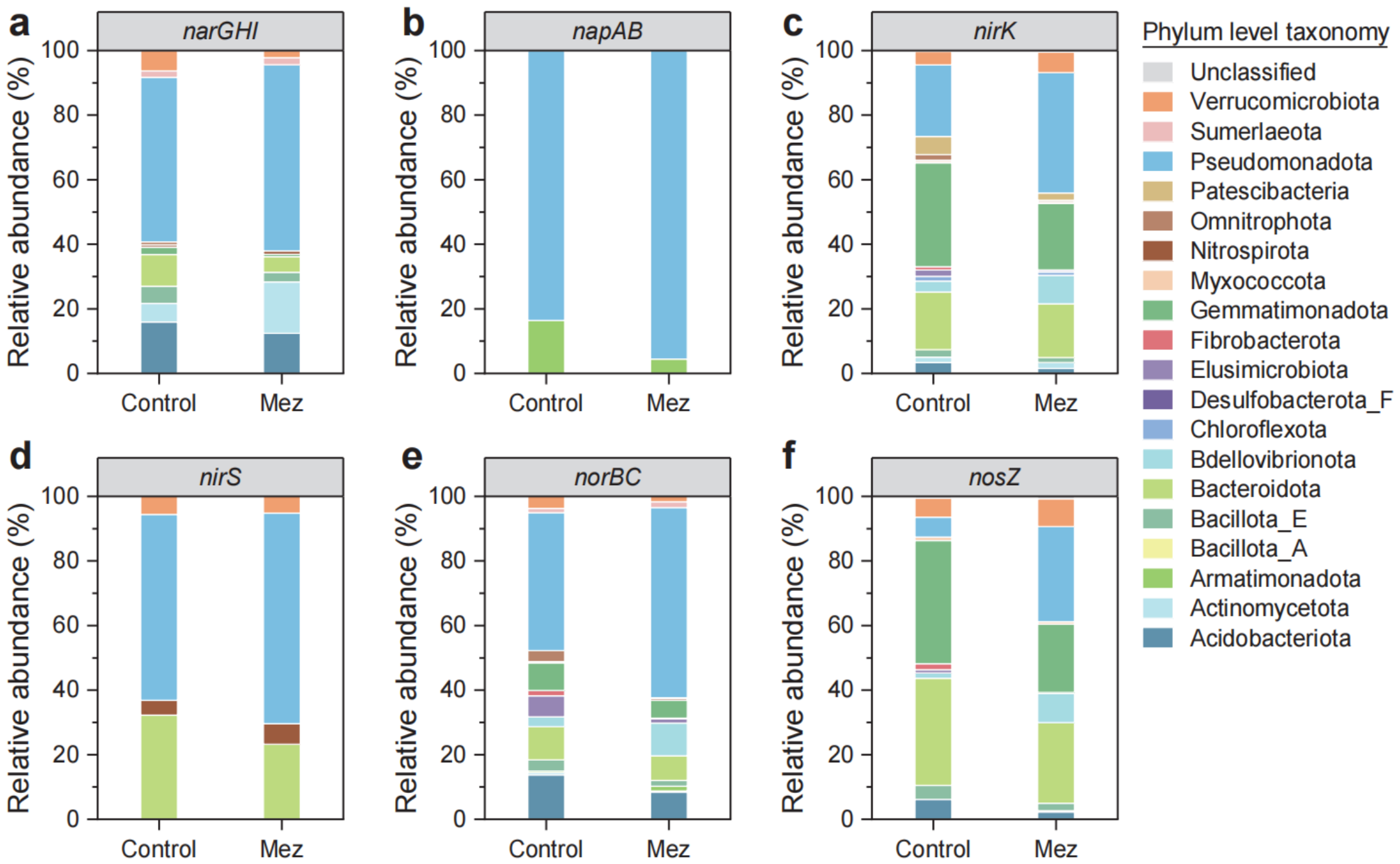

Supplementary Fig. 6: Taxonomic annotation of MAGs with denitrifying genes in response to Mez treatment. MAGs belonging to the **(a)** NO_3^- reductase (NarGHI), (**b**) NO_3^- reductase (NapAB), **(c)** and **(d)** NO_2^- reductase (NirK and NirS), **(e)** NO reductase (NorBC), **(f)** N_2O reductase (NosZ). MAGs were annotated at the phylum level. The unclassified MAGs reflect community members that are not well-represented in the reference databases. Data are presented as the mean of four independent biological replicate.

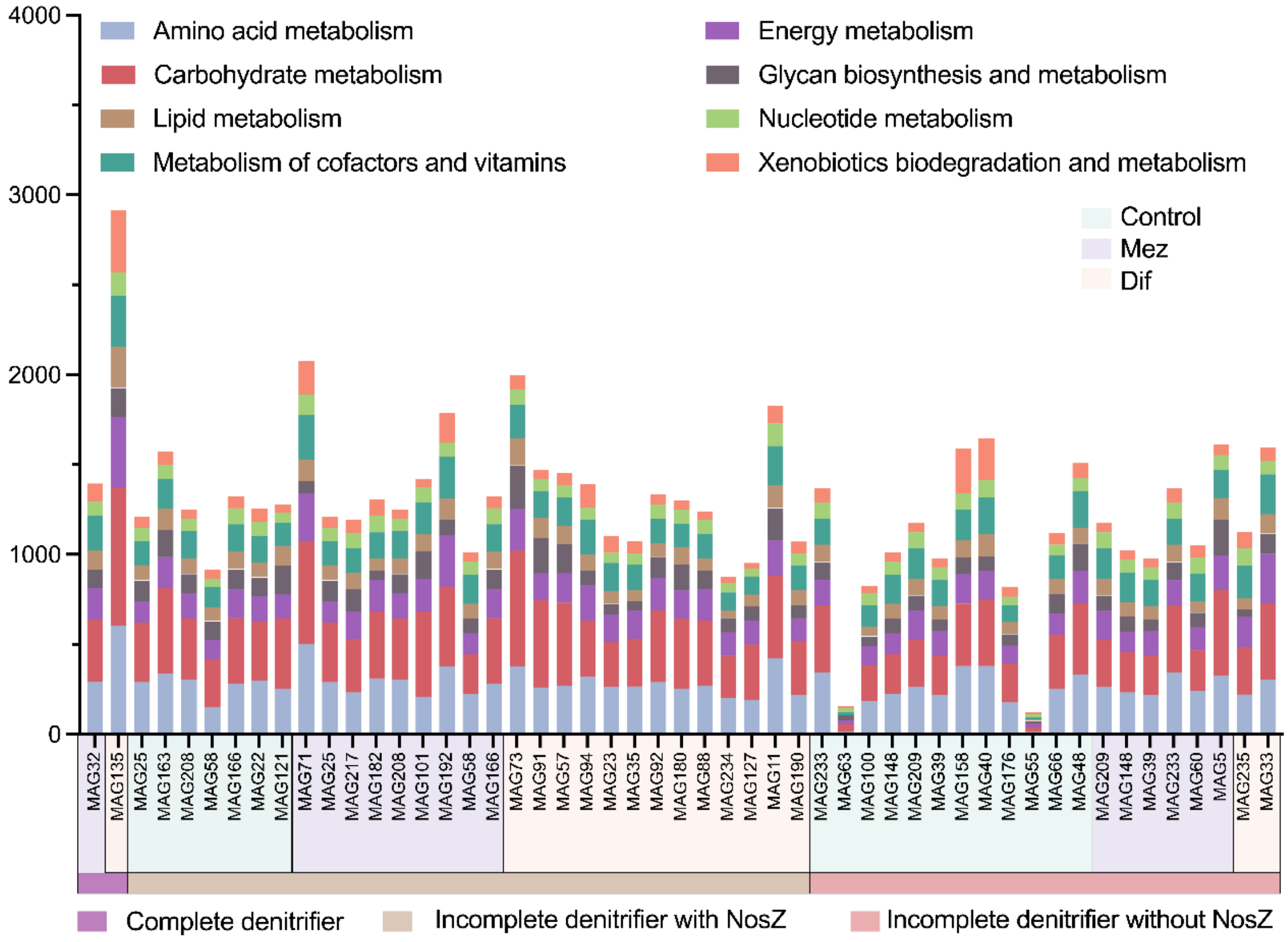

Supplementary Fig. 7: Clusters of orthologous groups (COG) pathway across soil denitrifiers. Bars are colored according to COG category and show the number of COGs identified from the KEGG pathway in the MAGs genomes. The COG pathways are classified as amino acid metabolism (light blue), energy metabolism (violet), carbohydrate metabolism (red), glycan biosynthesis and metabolism (brown), lipid metabolism (tan), nucleotide metabolism (green), cofactors and vitamins metabolism (cyan), and xenobiotics biodegradation and metabolism (orange). Data are presented as the mean of four independent biological replicate

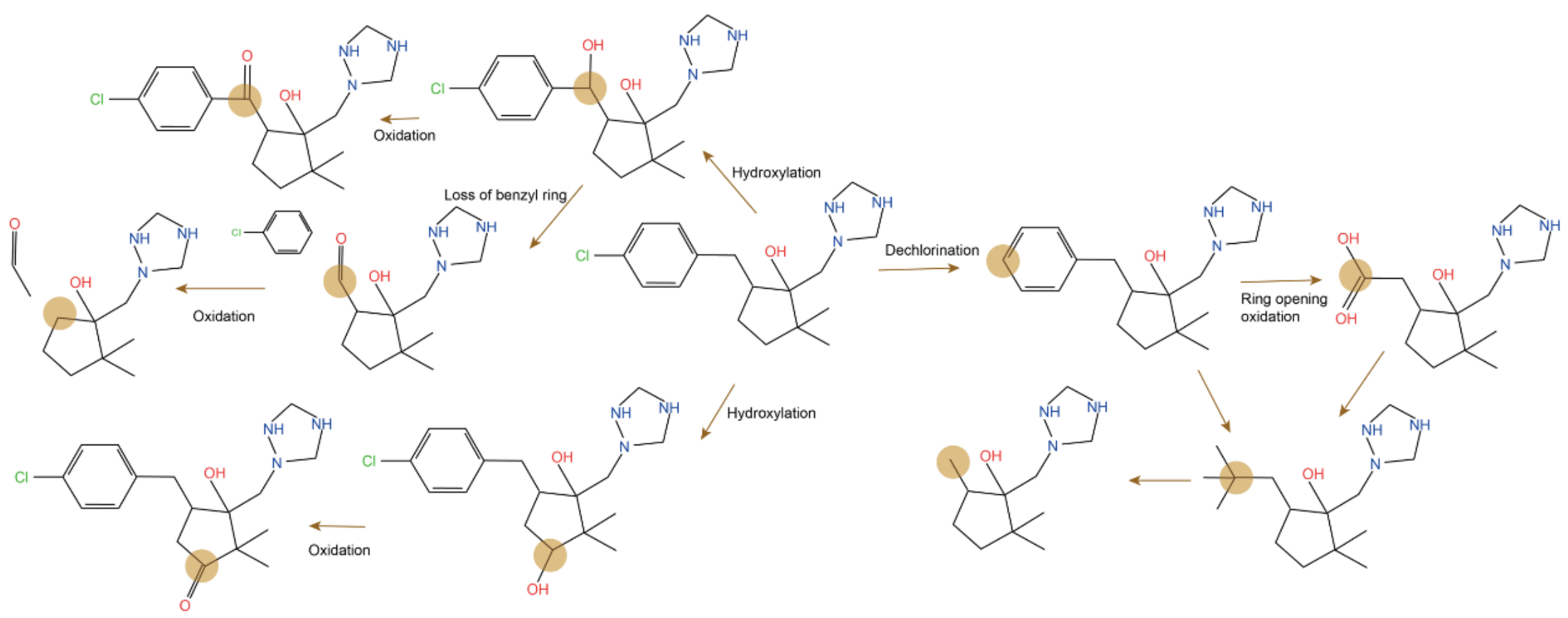

Supplementary Fig. 8: Mez potential degradation pathways.

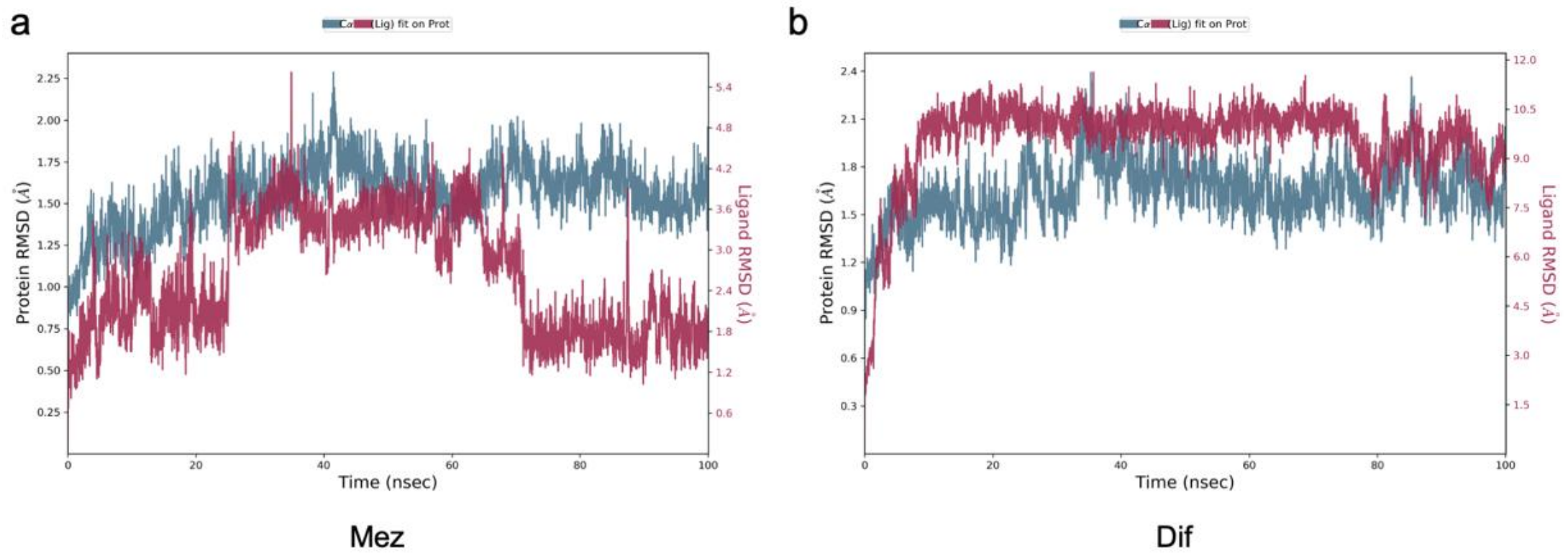

Supplementary Fig. 9: High Root Mean Square Deviation (RMSD) of P450Nor / triazole a complex during molecular dynamics simulations. (a) RMSD of P450Nor with Mez complex, **(b)** RMSD of P450Nor with Dif complex. The plot shows the temporal changes in the structural stability of the P450Nor (cobalt) and the triazole (crimson) binding over the course of the molecular dynamic simulations.

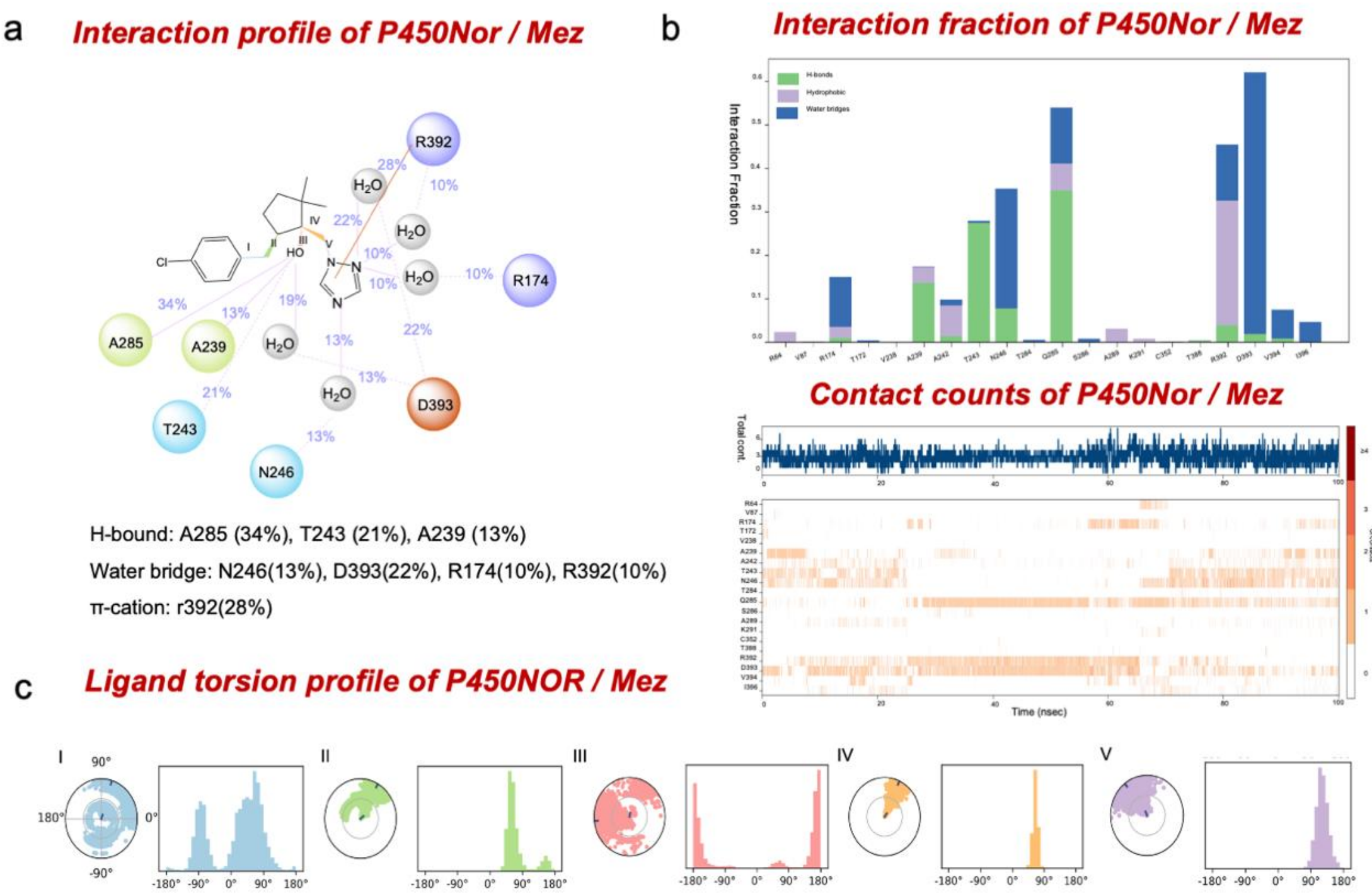

Supplementary Fig. 10: Conformational Stability analysis of P450Nor / Mez complexes during molecular dynamics simulation. (a) 2D interaction profile of P450Nor / Mez complex generated from the last frame of the last MD simulation. **(b)** Interaction fraction of P450Nor / Mez complex. **(c)** Ligand torsion profile of P450Nor / Mez complex.

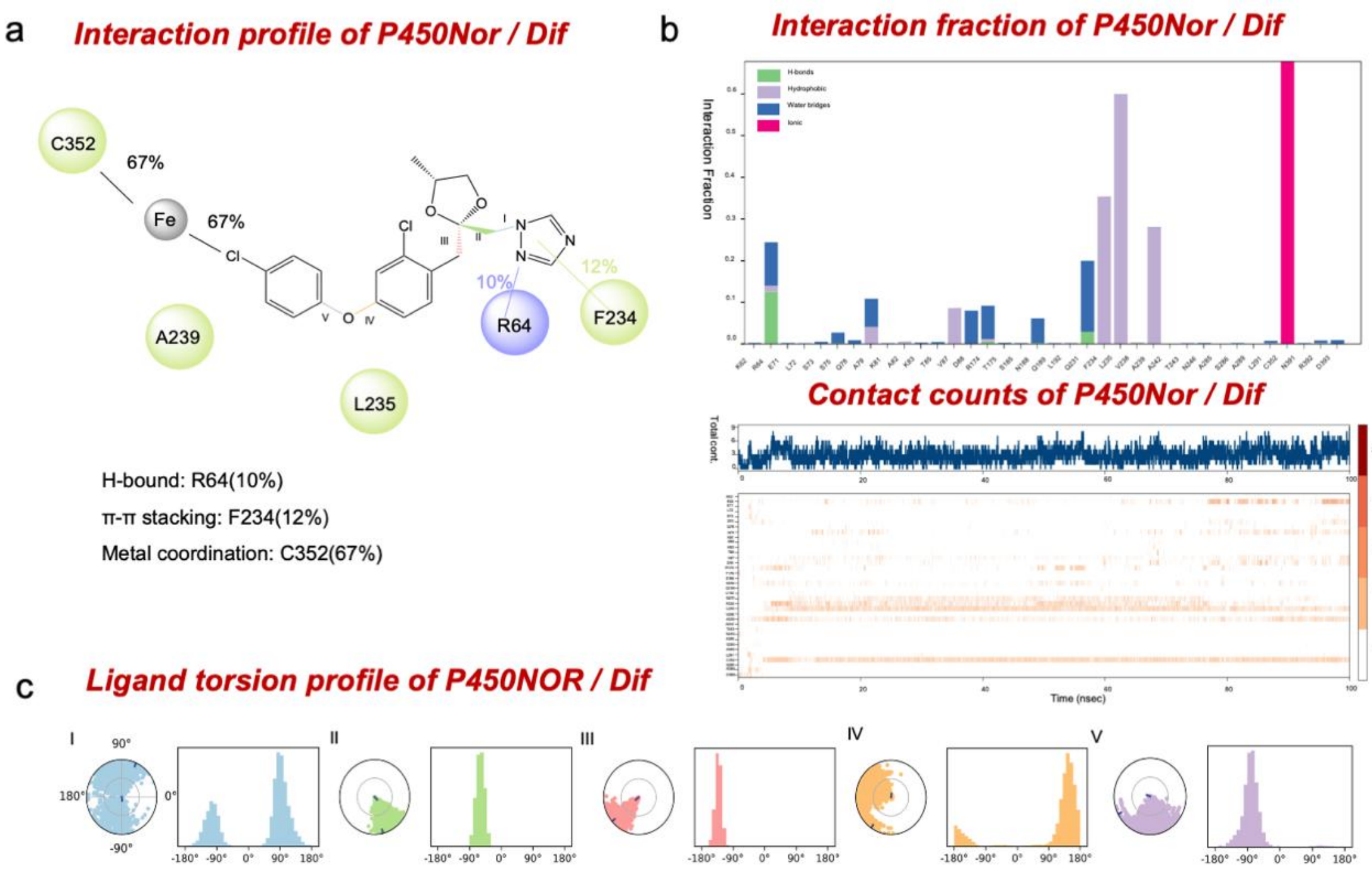

Supplementary Fig. 11: Conformational Stability analysis of P450Nor / Dif complexes during molecular dynamics simulation. **(a)** 2D interaction profile of P450Nor / Dif complex generated from the last frame of the last MD simulation. **(b)** Interaction fraction of P450Nor / Dif complex. **(c)** Ligand torsion profile of P450Nor / Dif complex.

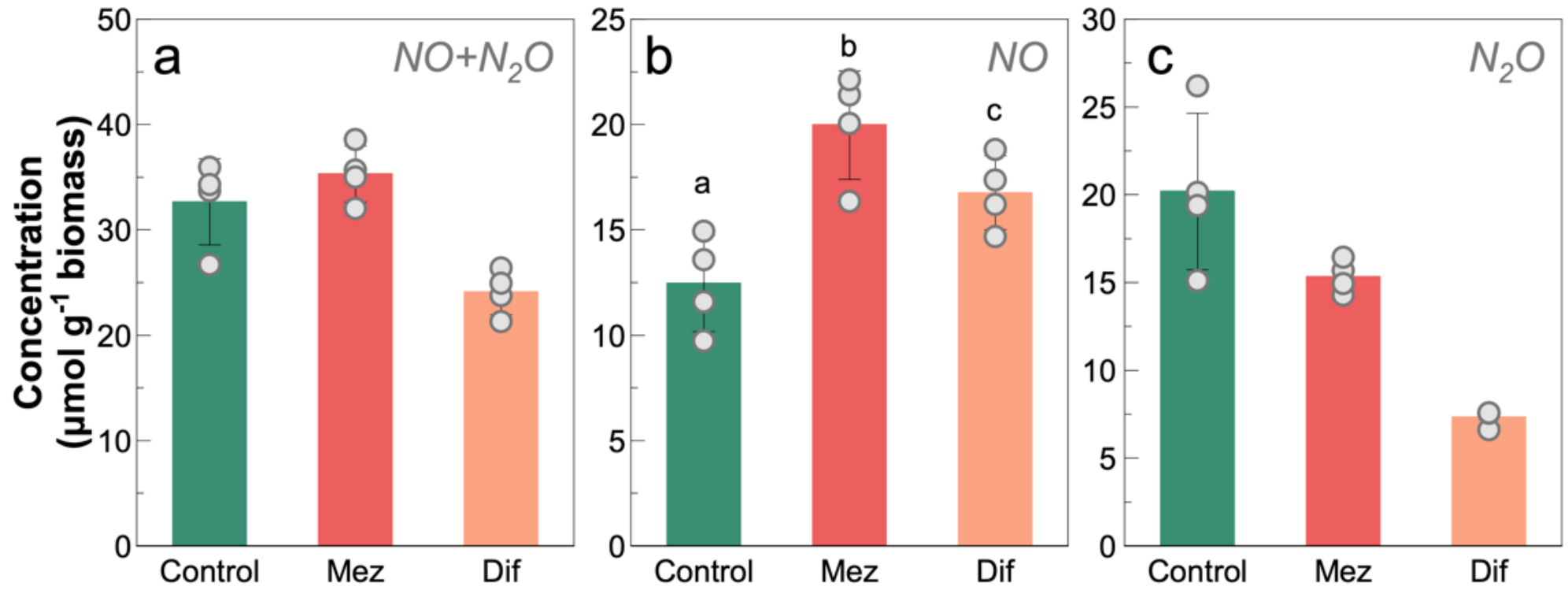

Supplementary Fig. 12: Concentrations of NO and N_2O by the denitrifying fungus *P. janthinellum* SM-12f4 during pure-cultural incubation after Mez and Dif applications. (a) Sum of NO and N_2O concentrations. **(b)** NO concentrations. **(c)** N_2O concentrations. Different letters indicate the significance (One-way ANOVA, $P<0.05$). Data are presented as mean ± SD (biological replicate, n=4).

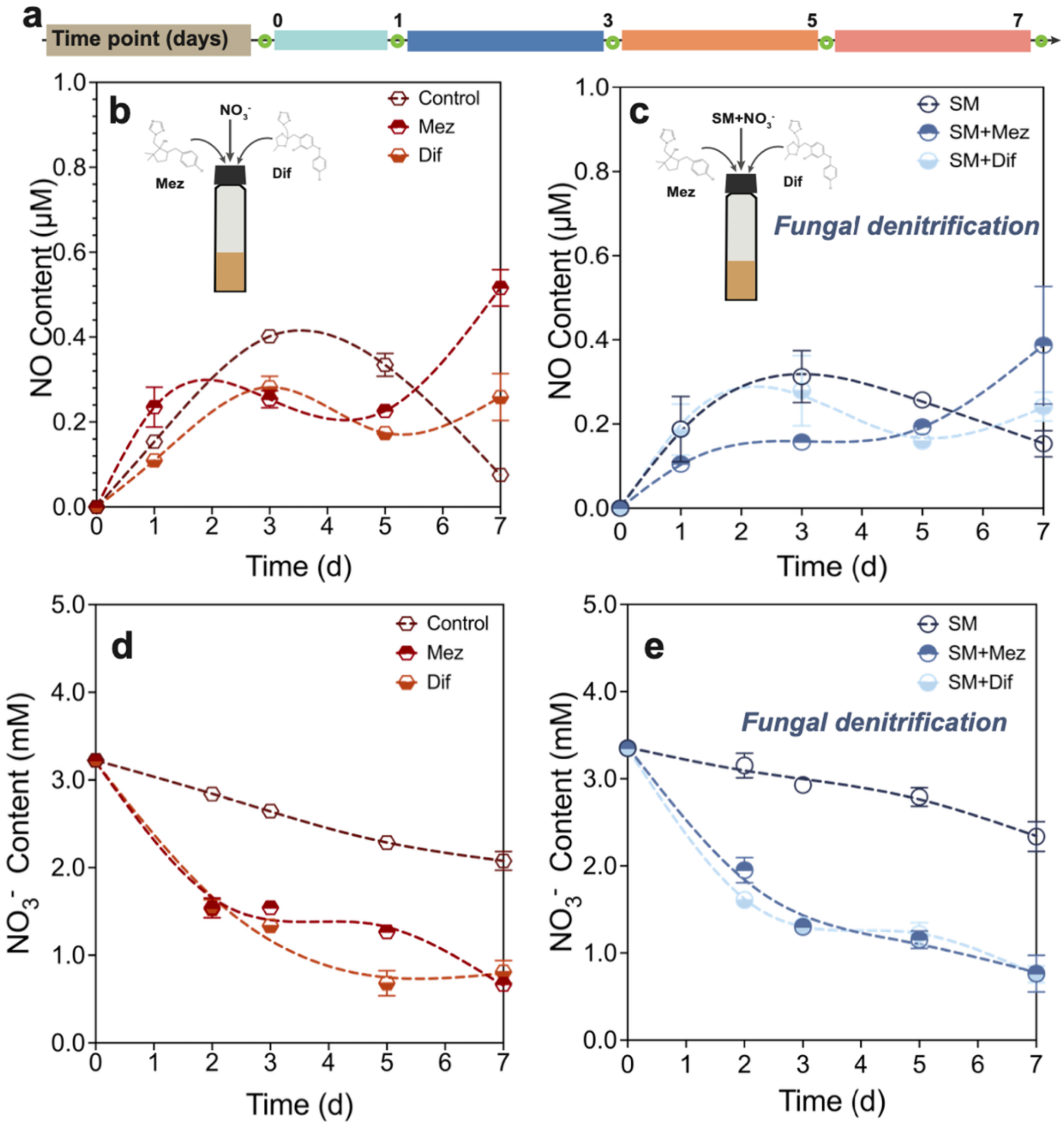

Supplementary Fig. 13: Variations of soil NO and NO_3^- concentrations during the 7-d incubation after Mez and Dif applications. (a) Sampling time-points for NO and NO_3^- measurement at day 0, 1, 3, 5 and 7. **(b)** Variations of NO concentrations in soil microcosms without adding any bacterial or fungal inhibitors. **(c)** Variations of NO concentrations in soil microcosms with adding bacterial inhibitor streptomycin (SM). **(d)** Variations of NO_3^- concentrations in soil microcosms without adding any bacterial or fungal inhibitors. **(e)** Variations of NO_3^- concentrations in soil microcosms with adding bacterial inhibitor streptomycin (SM). Data are presented as mean ± SD (biological replicate, n=4).

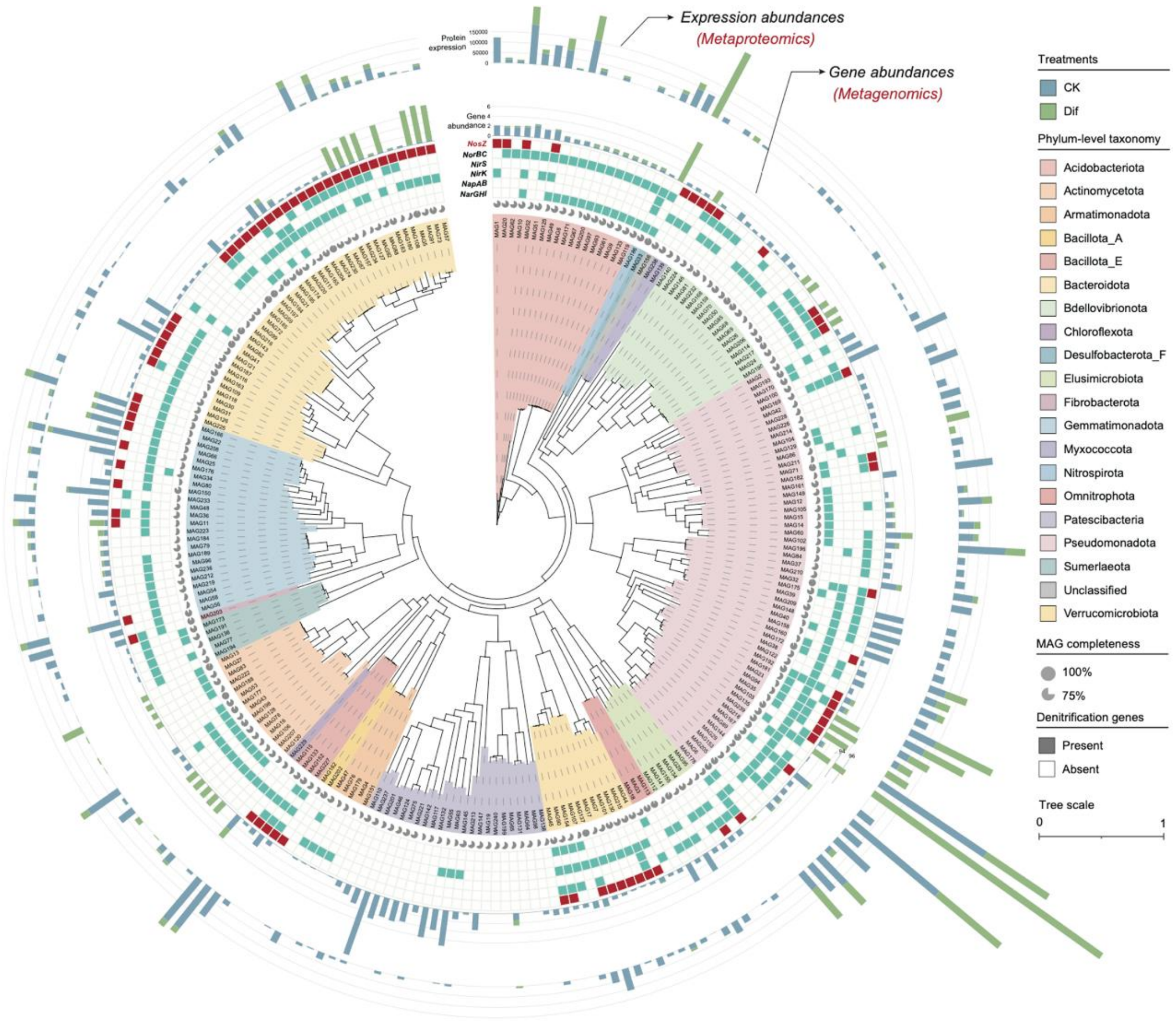

Supplementary Fig. 14: Maximum likelihood-based phylogenetic analysis of dereplicated 238 medium- and high-quality MAGs in response to Dif. Taxonomic classification of the MAGs was inferred using the Genome Taxonomy Database (GTDB) and is displayed at the phylum level by label and branch coloring. The bar chart shows the abundances (RPKM) of MAGs in control and Mez treatments at the gene (metagenomics) and expression (metaproteomics) levels. The heatmap indicates the MAGs genomes whether contain the *nosZ* gene (maroon) or other denitrification genes (cyan). The completeness and contamination of each MAG is represented by the pies at the branch tips.

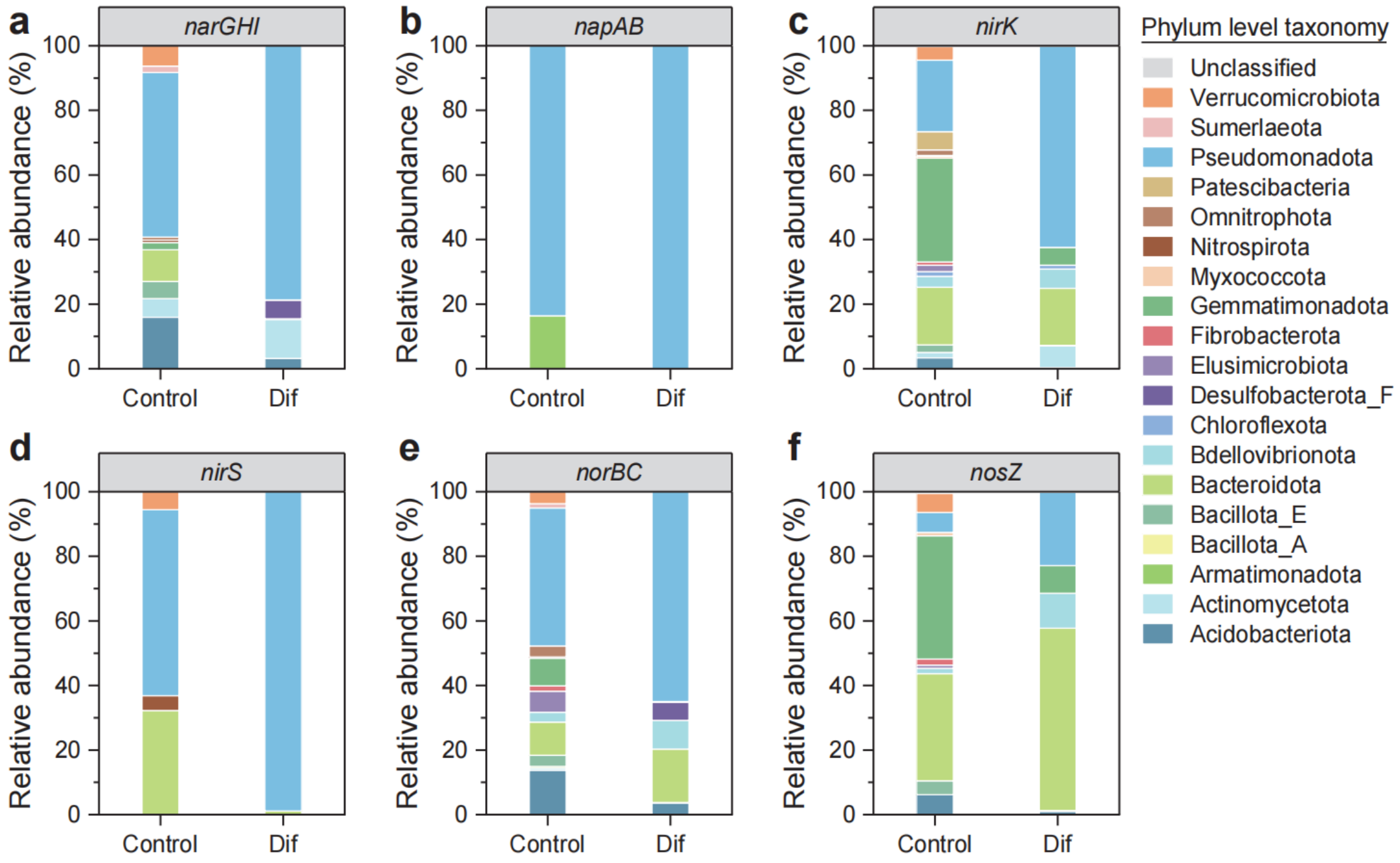

Supplementary Fig. 15: Taxonomic annotation of MAGs with denitrifying genes in response to Dif treatment. MAGs belonging to the **(a)** NO_3^- reductase (NarGHI), (**b**) NO_3^- reductase (NapAB), **(c)** and **(d)** NO_2^- reductase (NirK and NirS), **(e)** NO reductase (NorBC), **(f)** N_2O reductase (NosZ). MAGs were annotated at the phylum level. The unclassified MAGs reflect community members that are not well-represented in the reference databases. Data are presented as the mean of four independent biological replicate.

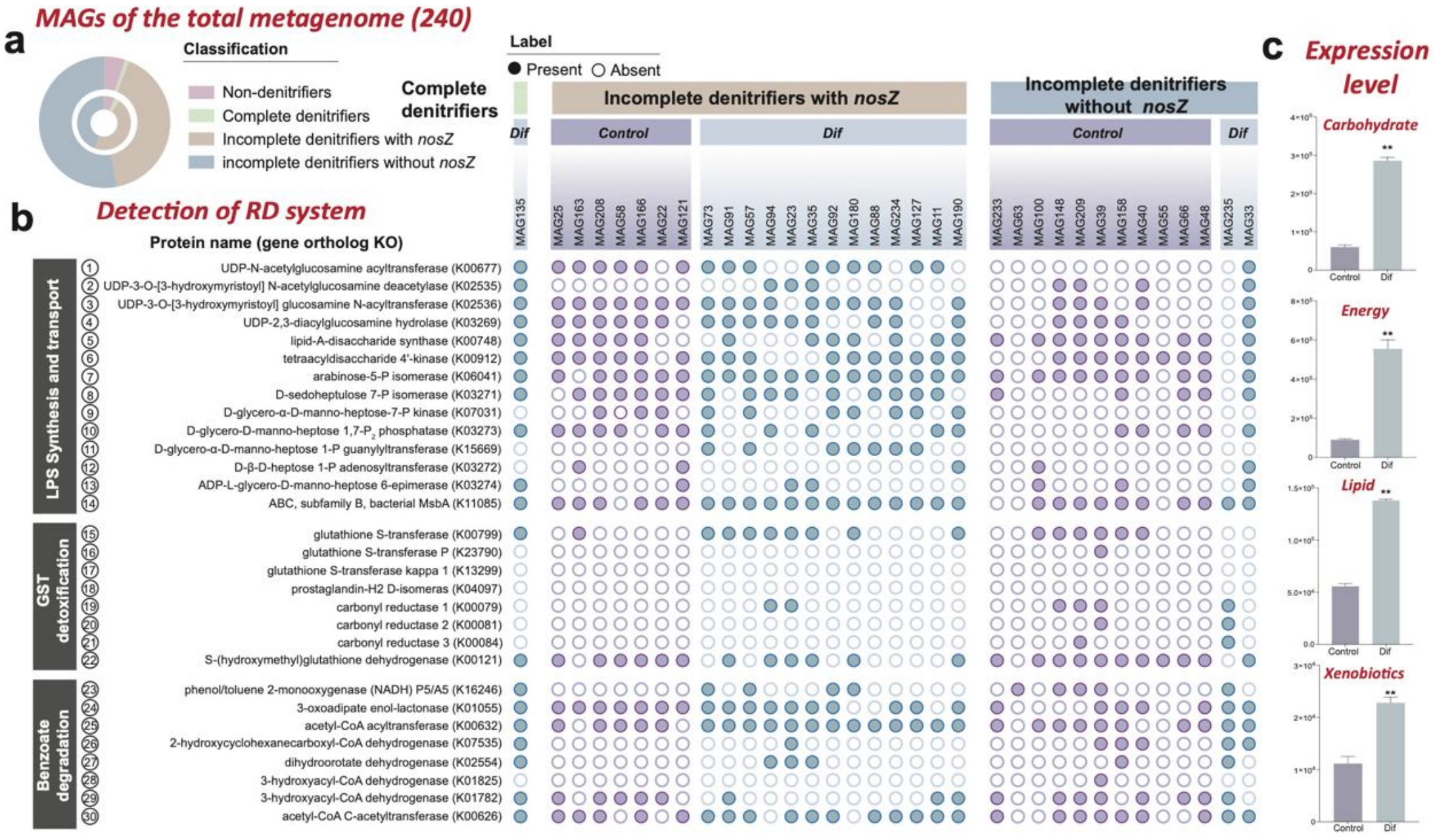

Supplementary Fig. 16: Proportions of denitrifying MAGs and postulated pathways of triazole resistance and detoxification (RD) from bacterial denitrifying MAGs. (a) Proportions of non-denitrifiers, complete denitrifiers, and incomplete denitrifiers with and without *nosZ* in the MAGs of the total metagenomes. (**b**) Overview of the completeness of bacterial triazole RD systems in complete and incomplete denitrifiers. (**c**) Protein-level expression of the Triazole RD system. Asterisk (*) indicates statistical significance (one-way ANOVA, ** P<0.001). Data are presented as mean±SD (sample numbers, n=14~91).

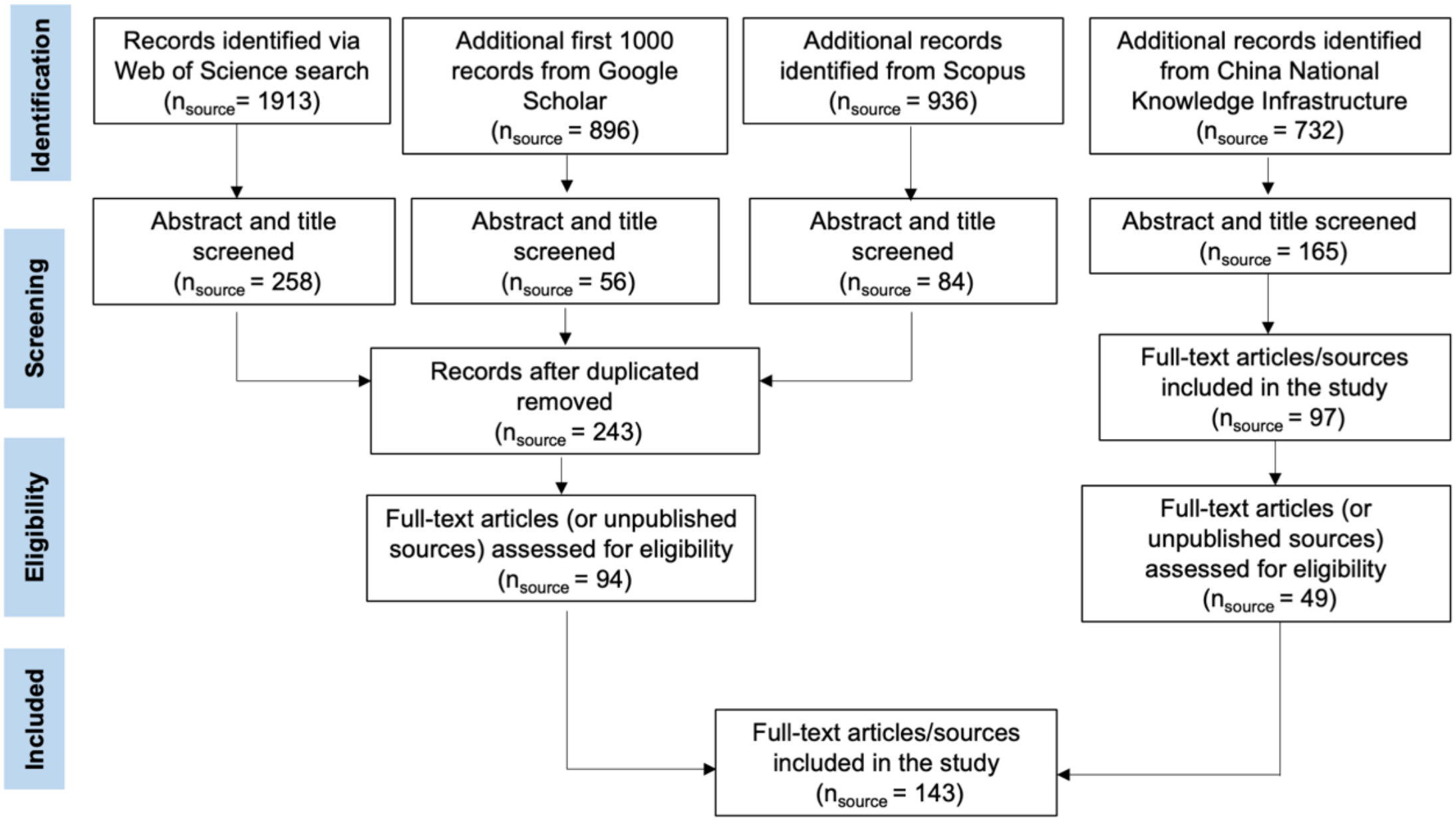

Supplementary Fig. 17: PRISMA flow chart for global data collection of environmentally relevantly concentration of Dif and its toxicity assessment on various organisms. Studies were identified through literature search of 'Web of Science', 'Google Scholar', ' Scopus' and 'China National Knowledge Infrastructure'. We searched for articles published up to March 12, 2025.

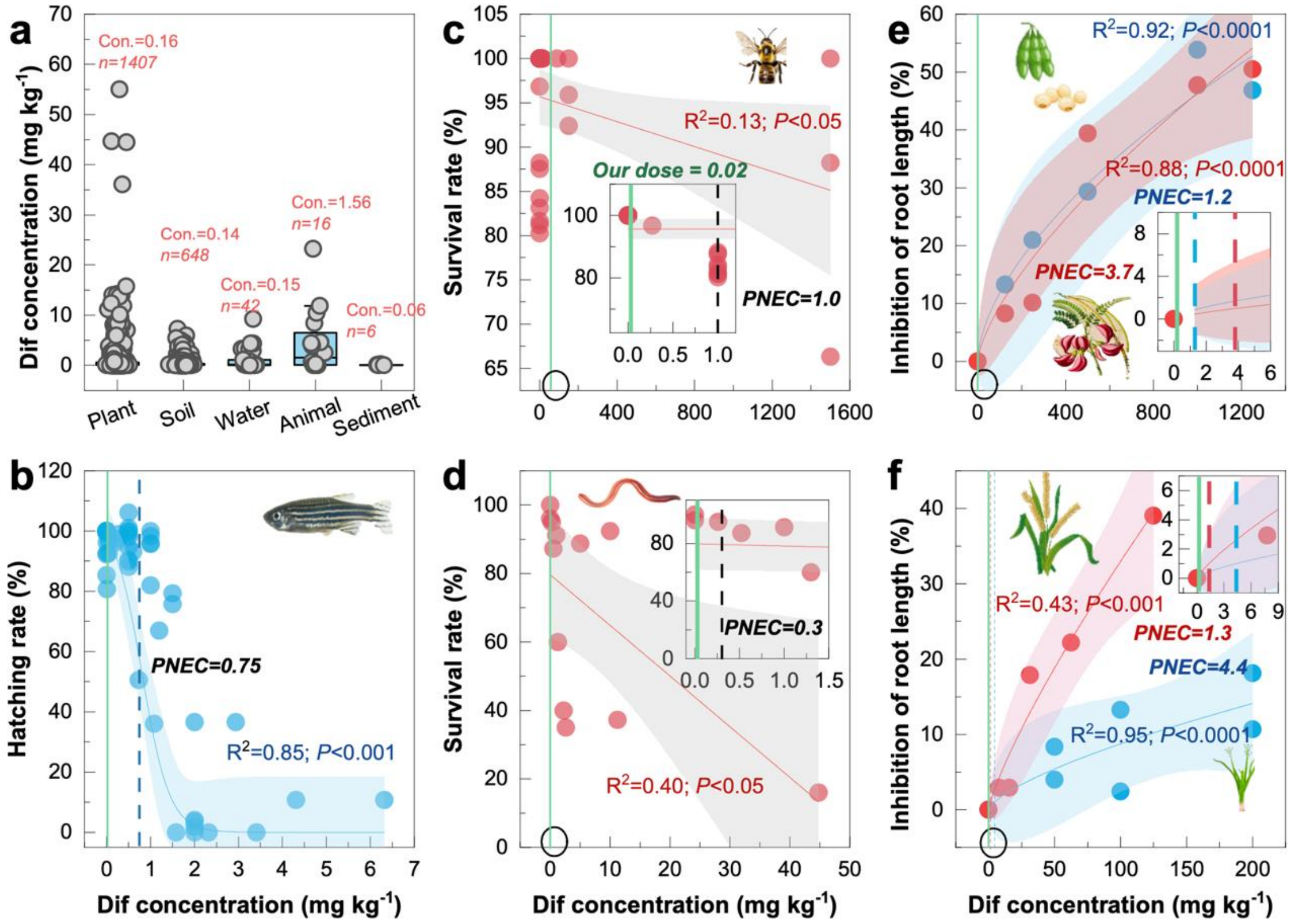

Supplementary Fig. 18: Ecologically relevant concentrations of Dif and its estimated ecological toxicity on aquatic and terrestrial species, as well as plants, based on a global meta-analysis. (a) Residual concentrations of Dif across various environments. **(b)** Hatching rate (%) of zebrafish embryos exposed to different Dif concentrations in aquatic ecosystems. **(c)-(d)** Survival rates (%) of bees and earthworms exposed to various Dif concentrations in terrestrial ecosystems. **(e)-(f)** Root length inhibition (%) in soybean, sesbania, wheat, and garlic plants exposed to different Dif concentrations. 'Con.' represents Dif concentration; 'n' is the sample size; 'PNEC' denotes the predicted no-effect concentration for organisms. The applied Dif dose in our study is 0.02 mg/kg (green line), and the PNEC values for organisms in previous studies are indicated by black, red, or blue lines. Grey areas represent the 95% confidence interval. Insets in **(c)**, **(d)**, **(e)**, and **(f)** highlight the X-axis ranges as indicated by black circles.

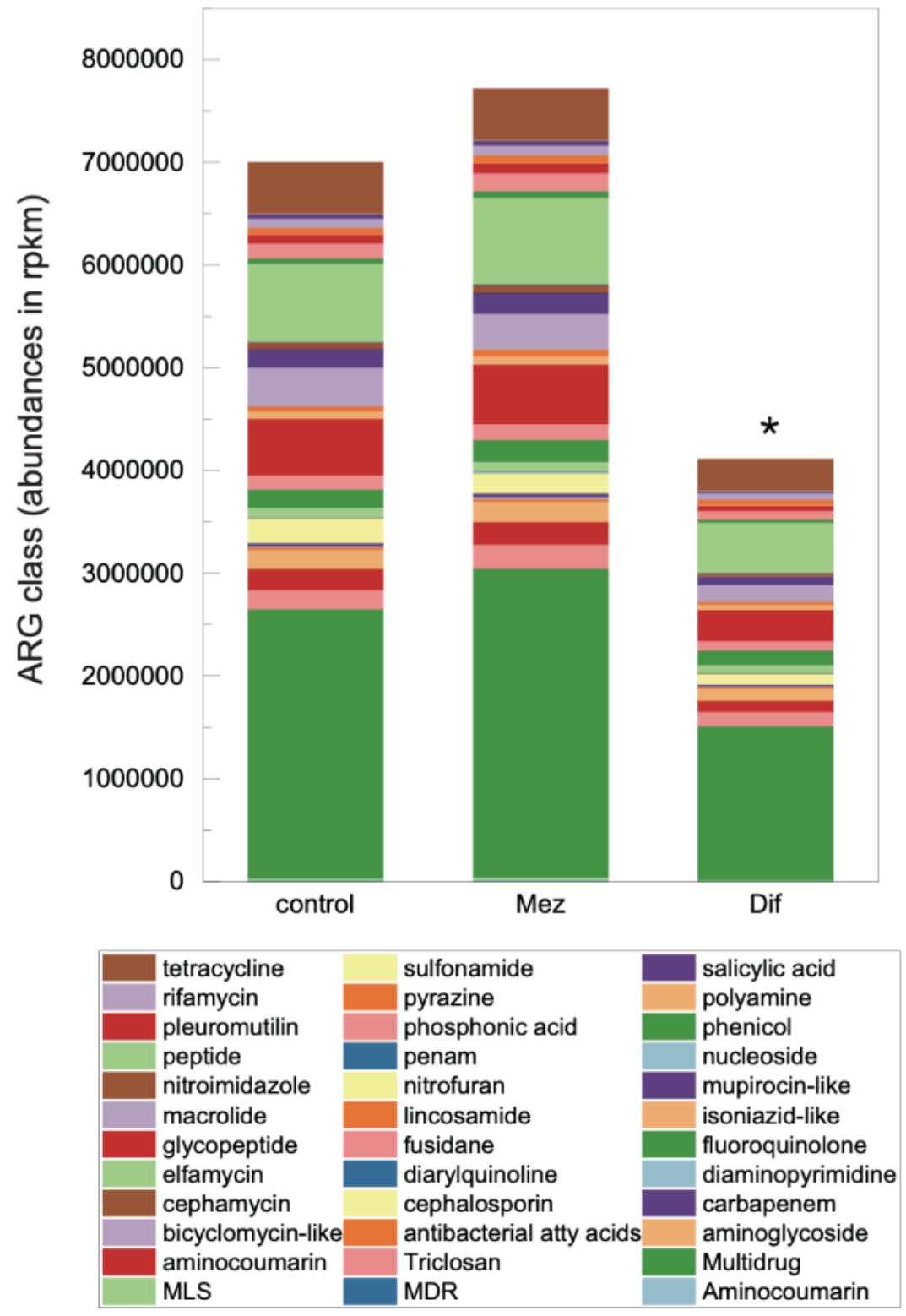

Supplementary Fig. 19: The abundance of soil antibiotic resistance genes (ARGs) in response to Mez and Dif treatments. Data are presented as the mean of four independent biological replicate. Asterisk indicates the significance using Kruskal-Wallis test under $P<0.05$.

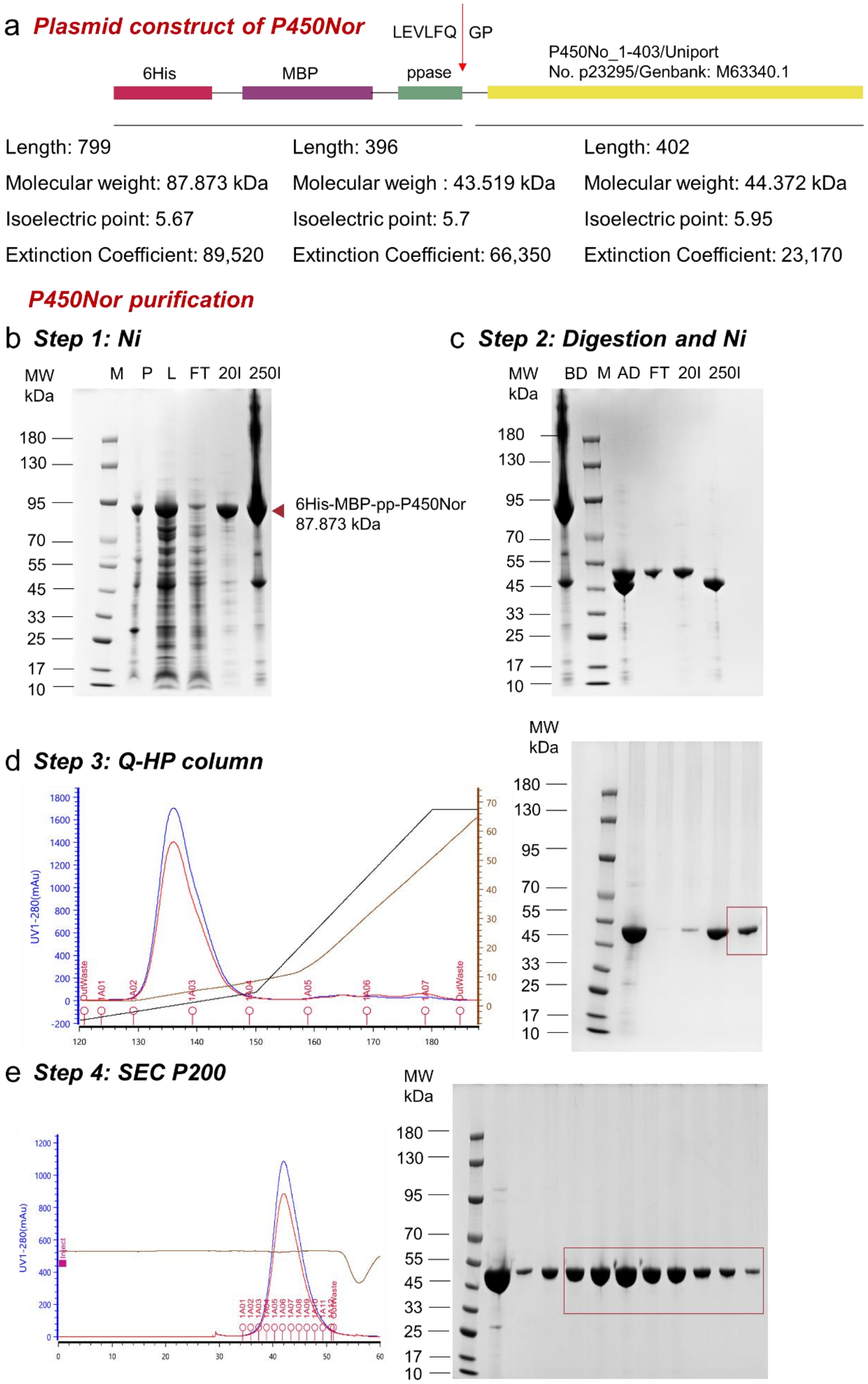

Supplementary Fig. 20: The expression and purification of P450Nor protein. (a)

P450Nor protein plasmid construct with 6His, MBP, and ppase. **(b)** Purification by nickel (Ni-NTA) column. **(c)** Digestion by PPase and purification by Ni-NTA column. **(d)** Purification by HiTrap Q HP column. (**e)** Purification by P200 10 500 molecular sieve.

Reference

1 Chen, S., Zhou, Y., Chen, Y. & Gu, J. Fastp: an ultra-fast all-in-one FASTQ preprocessor. *Bioinformatics* **34**, i884-i890 (2018).

2 Li, H. & Durbin, R. Fast and accurate short read alignment with Burrows-Wheeler transform. *Bioinformatics* **25**, 1754-1760 (2009). https://doi.org/10.1093/bioinformatics/btp324

3 Li, D., Liu, C.-M., Luo, R., Sadakane, K. & Lam, T.-W. MEGAHIT: an ultra-fast single-node solution for large and complex metagenomics assembly via succinct de Bruijn graph. *Bioinformatics* **31**, 1674-1676 (2015).

4 Fu, L., Niu, B., Zhu, Z. & Li, W. CD-HIT: accelerated for clustering the next-generation sequencing data. *Bioinfomatics* **28**, 3150-5152 (2012).

5 Langmead, B. & Salzberg, S. L. Fast gapped-read alignment with Bowtie. *Nature Methods* **9**, 357-359 (2012).

6 Qin, J., Li, R., Raes, J. & Wang, J. A human gut microbial gene catalogue established by metagenomic sequencing. *Nature* **464**, 357-359 (2010).

7 Huson, D. H. & Buchfink, B. Fast and sensitive protein alignment using DIAMOND. *Nature Methods* **12**, 59-60 (2015).

8 Johansen, J. *et al.* Genome binning of viral entities from bulk metagenomics data. *Cold Spring Harbor Laboratory* (2021).

9 Donovan, H. *et al.* CheckM: assessing the quality of microbial genomes recovered from isolates, single cells, and metagenomes. *Genome research* (2015).

10 Parks, D. H. *et al.* GTDB: An ongoing census of bacterial and archaeal diversity through a phylogenetically consistent, rank normalizedand complete genome-based taxonomy. *Nucleic Acids Research* **50**, 785-794 (2022). https://doi.org/10.1093/nar/gkab776

11 Salmon, P. R. Salmon: accurate, versatile and ultrafast quantification from RNA-seq data using lightweight-alignment. *Cold Spring Harbor Labs Journals* (2015).

12 Cox, J. *et al.* Accurate Proteome-wide Label-free Quantification by Delayed Normalization and Maximal Peptide Ratio Extraction, Termed MaxLFQ*. *Molecular & Cellular Proteomics* **13**, 2513-2526 (2014). https://doi.org/https://doi.org/10.1074/mcp.M113.031591

13 Tyanova, S. *et al.* The Perseus computational platform for comprehensive analysis of (prote)omics data. *Nature Methods* **13**, 731-740 (2016). https://doi.org/10.1038/nmeth.3901

14 Jonassen, K. R. *et al.* Nitrous oxide respiring bacteria in biogas digestates for reduced agricultural emissions. *The ISME Journal* **16**, 580-590 (2021). https://doi.org/10.1038/s41396-021-01101-x
