# Supplementary material for: Structure-guided identification of denitrification inhibitors to mitigate agricultural N2O emissions
Source: Nat Commun. 2026 Aug 20;17:9944. doi: 10.1038/s41467-026-76975-6 (PMC13586399; doi:10.1038/s41467-026-76975-6)
Supplement: Supplementary file 2 [file 41467_2026_76975_MOESM2_ESM.pdf]

nature portfolio

Peer Review File

Structure-guided identification of denitrification inhibitors to mitigate agricultural N2O emissions

Corresponding Author: Professor Xiaoxuan Su

This file contains all reviewer reports in order by version, followed by all author rebuttals in order by version.

Version 0:

Reviewer comments:

Reviewer #1

(Remarks to the Author)
The manuscript outlines an assessment of the efficacy of a denitrification inhibitor (Mez) on nitrogen transformations. It also outlines a series of genomic experiments to further determine the mechanisms underlying this inhibition. Firstly, soil incubations at 70% WFPS do no target denitrification alone, as nitrification allows operates at this water content. Why wasn't a higher soil WFPS e.g. 85% utilised. Secondly, the statements in the Results Section 3.1 are potentially misleading of the efficacy of Mez e.g.
l341 - "....Mez likely promoted...."
l343 - was this statistically significant? There is no P value as per other areas in the manuscript.
l352 - ".....Mez substantially reduced...."
l353 - "..notably inhibited N2O...."
Supplementary Figure S4 A and B - were error bars presented?

Reviewer #2

(Remarks to the Author)
C17H22ClN3O， mez, is a broad-spectrum, systemic triazole-based fungicide. The author used it as denitrification inhibitor, the research is interesting.
Some questions should be answered.
1． The effect of mez on archaea in soil should be analyzed, such as, DAMO, AOA.
2． The effect of mez on anammox in soil should also be analyzed, the nitrogen cycle related gene should be analyzed completely.
3． The degradation products of mez should be analyzed.
4． The effects of mez on the antibiotic resistance genes should be carried out.
5． The English should be polished.

Version 1:

Reviewer comments:

Reviewer #1

(Remarks to the Author)
No further comments

Reviewer #2

(Remarks to the Author)
The authors answered my questions, it can be accepted for publication.

Open Access This Peer Review File is licensed under a Creative Commons Attribution 4.0 International License, which permits use, sharing, adaptation, distribution and reproduction in any medium or format, as long as you give appropriate credit to the original author(s) and the source, provide a link to the Creative Commons license, and indicate if changes were made.
In cases where reviewers are anonymous, credit should be given to 'Anonymous Referee' and the source.
The images or other third party material in this Peer Review File are included in the article's Creative Commons license, unless indicated otherwise in a credit line to the material. If material is not included in the article's Creative Commons license and your intended use is not permitted by statutory regulation or exceeds the permitted use, you will need to obtain permission directly from the copyright holder.
To view a copy of this license, visit https://creativecommons.org/licenses/by/4.0/

Replies and Explanations to Comments

We thank the reviewers for their constructive comments. We have now expanded our data analyses as suggested by the reviewers. We have now revised the manuscript thoroughly and highlighted the changes in track-change mode. All questions raised by the reviewers have been answered point-by-point below.

Reviewer #1 Comments:

The manuscript outlines an assessment of the efficacy of a denitrification inhibitor (Mez) on nitrogen transformations. It also outlines a series of genomic experiments to further determine the mechanisms underlying this inhibition.

1. Firstly, soil incubations at 70% WFPS do no target denitrification alone, as nitrification allows operates at this water content. Why wasn't a higher soil WFPS e.g. 85% utilized.

Answer:

Thanks. We agree that nitrification could occur at 70% WFPS under oxic or partially oxic conditions. However, this moisture level already falls within a range favorable to denitrification, as restricted gas diffusion facilitates the formation of anaerobic microsites within soil aggregates. More importantly, our incubation experiment was conducted under anaerobic conditions (purged with He gas); thus, nitrification was expected to be strongly suppressed in this study.

In addition, we chose 70% WFPS to represent moist agricultural soil conditions more broadly, rather than imposing near-saturated or flooded conditions that would be more specific to paddy soils or waterlogged systems. We have now revised the statements to avoid any confusion.

"*Subsequently, these bottles were anaerobically incubated at 25℃ in the dark*

for 28 d with 70% water-filled pore space (WFPS), a moisture level favorable for denitrification in moist agricultural soils. " (Line 518).

2. Secondly, the statements in the Results Section 3.1 are potentially misleading of the efficacy of Mez, e.g.:

l341 - "....Mez likely promoted...."

l343 - was this statistically significant? There is no P value as per other areas in the manuscript.

l352 - ".....Mez substantially reduced...."

l353 - "..notably inhibited N2O...."

Answer:

Sorry for the confusion, and the text has been revised accordingly. We have also provided the corresponding *P* values in the revised manuscript.

"*The presence of Mez was associated with increased NO_3^- and NO_2^- reduction and NH_4^+ production, suggesting a potential stimulation of soil dissimilatory NO_3^- reduction to NH_4^+ process (DNRA; Supplementary Fig. 2a-d).*" (Line 135).

"*More importantly, we found that N_2O emissions were significantly reduced by Mez ($P<0.001$), with the inhibition ranging from 85 to 100% (Fig. 1a).*" (Line 137).

"*Consistent with the 28-d incubation results, Mez substantially reduced the total N_2O emissions ($P<0.001$, Supplementary Fig. 2e), and it notably inhibited N_2O emissions by fungi while slightly reducing bacterial N_2O emissions ($P<0.001$, Fig. 1c and d).*" (Line 147).

3. Supplementary Figure S4 A and B - were error bars presented?

Answer:

Thanks. The error bars in this figure were inadvertently plotted as mean±SEM

rather than mean±SD. Consequently, the error bars appeared very small and were difficult to distinguish in the figure. We have now corrected the figure by presenting the data as mean±SD (please see the figure below). To further improve transparency, we have provided the corresponding numerical values below.

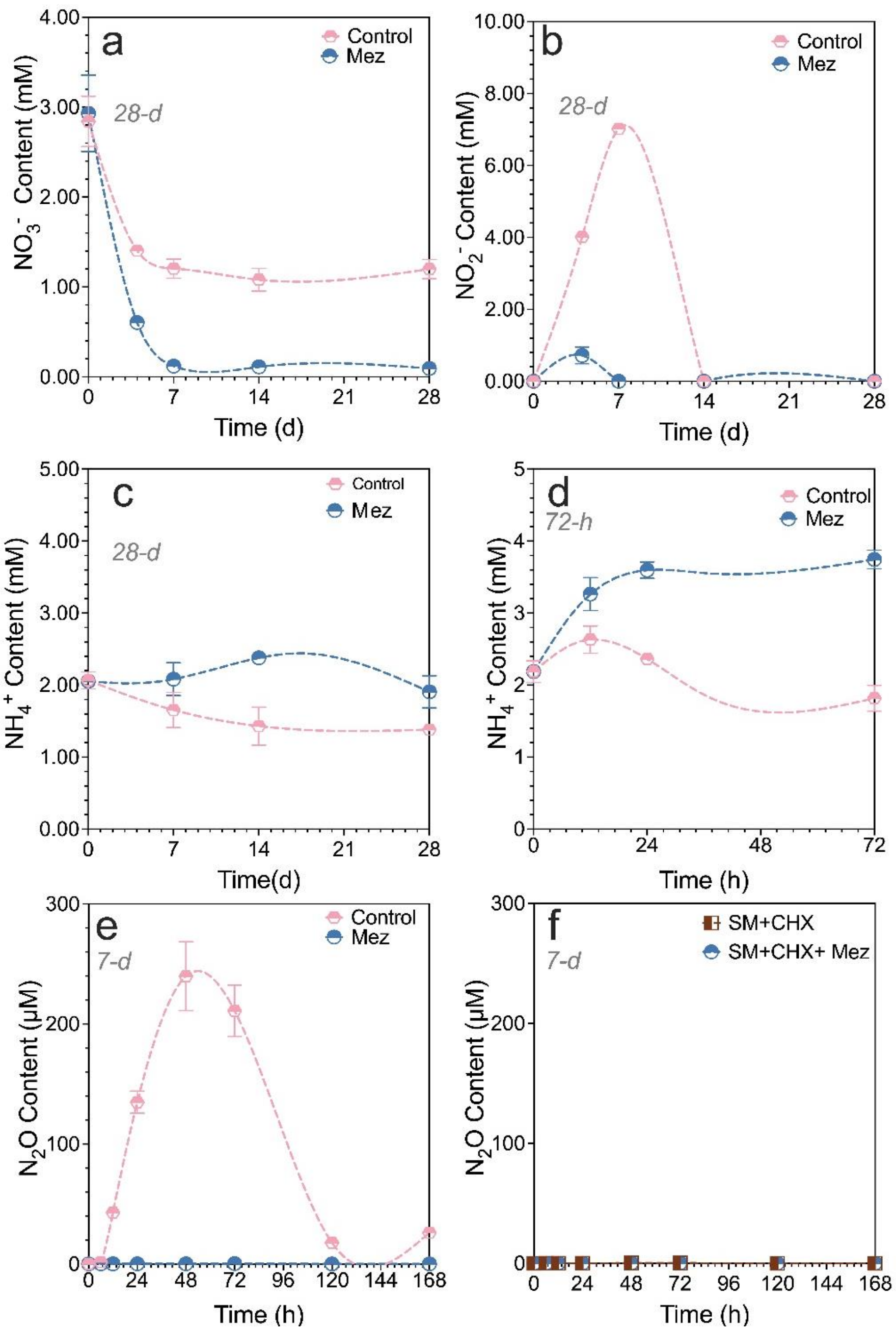

Response to comments Fig 1 (now added in the Supporting Information, **Fig. S2**)**. Variations of soil nitrogen speciation (NO_3^-, NO_2^-, NH_4^+, and N_2O) in response to Mez. (a)**, **(b)**, and **(c)** Changes in the concentrations of NO_3^-, NO_2^-, and NH_4^+ along with time during a 28-d incubation.

(d) Changes in the concentrations of NH_4^+ along with time during a 72-h incubation. **(e)** and **(f)** Changes in the concentrations of N_2O along with time during a 7-d incubation. Data are presented as mean ± SD (n=4).

Response to comments Table 1. The concentration of NO_3^- in soil during 28-d incubation

Time (h)	Control (mg/kg)		Mez (mg/kg)	
	mean	SD	mean	SD
0	2.842	0.281	2.932	0.424
4	1.405	0.042	0.606	0.056
7	1.205	0.106	0.120	0.024
14	1.081	0.125	0.112	0.035
28	1.199	0.106	0.095	0.019

Response to comments Table 2. The concentration of NO_2^- in soil during 28-d incubation

Time (h)	Control (mg/kg)		Mez (mg/kg)	
	mean	SD	mean	SD
0	0	0	0	0
4	4.028	0.011	0.719	0.228
7	7.128	0.096	0.003	0.005
14	0	0	0	0
28	0	0	0	0

Reviewer #2 Comments:

C17H22ClN3O， mez, is a broad-spectrum, systemic triazole-based fungicide. The author used it as denitrification inhibitor; the research is interesting.

4. The effect of mez on archaea in soil should be analyzed, such as, DAMO, AOA.

Answer:

Thanks. We have now supplemented the analysis of soil archaeal community,

including archaeal groups potentially associated with nitrogen cycling. The results showed that Mez had no significant effect on either the composition or the overall structure of the archaeal community under the conditions tested. We have added the results in the revised manuscript (please see the figure and table below).

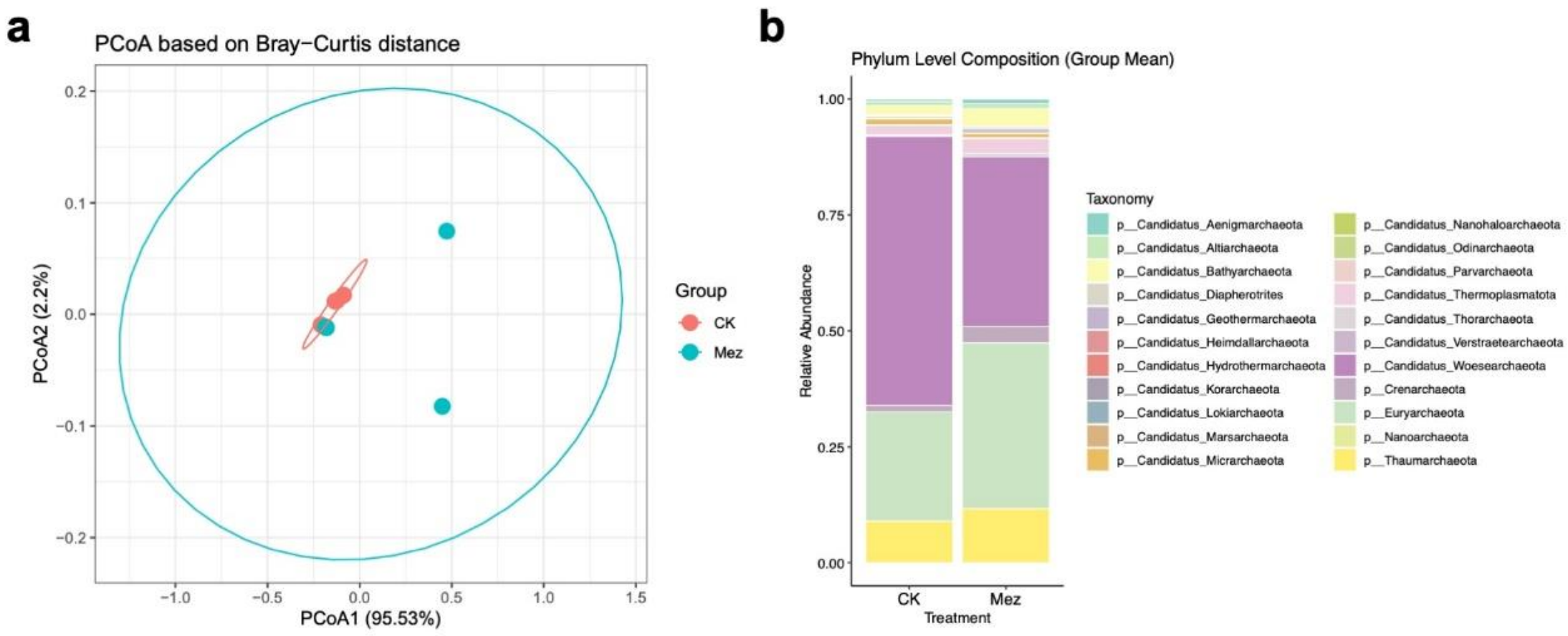

Response to comments Fig 2 (now added in the Supporting Information, **Fig. S3**)**.** Soil archaeal community in response to Mez. (a) Principal coordinate analysis (PCoA) based on Bray–Curtis distance showing the archaeal community structure in the control (CK) and metconazole (Mez) treatments. (b) Relative abundances of archaeal taxa at the phylum level in the CK and Mez treatments.

Response to comments Table 3. Statistical comparisons of archaeal phylum-level relative abundance between the control and metconazole treatments.

Taxonomy	*P*	Taxonomy	*P*
p__Candidatus_Parvarchaeota	0.494	p__Candidatus_Geothermarchaeota	0.914
p__Candidatus_Thorarchaeota	0.494	p__Candidatus_Thermoplasmatota	0.914
p__Candidatus_Aenigmarchaeota	0.494	p__Candidatus_Verstraetearchaeota	0.914
p__Candidatus_Altiarchaeota	0.494	p__Candidatus_Woesearchaeota	0.914
p__Candidatus_Heimdallarchaeota	0.494	p__Crenarchaeota	0.914
p__Candidatus_Odinarchaeota	0.711	p__Candidatus_Diapherotrites	1.000
p__Candidatus_Hydrothermarchaeota	0.763	p__Candidatus_Marsarchaeota	1.000
p__Candidatus_Lokiarchaeota	0.763	p__Candidatus_Micrarchaeota	1.000
p__Euryarchaeota	0.763	p__Candidatus_Nanohaloarchaeota	1.000

p__Candidatus_Korarchaeota	0.914	p__Nanoarchaeota	1.000
p__Candidatus_Bathyarchaeota	0.914	p__Thaumarchaeota	1.000

"*At the community level, Mez had no significant effect on archaeal community composition and structure (Supplementary Fig. 3), suggesting that archaea were unlikely to be primary microbial targets responsible for the observed reduction in N_2O emissions.*" (Line 161).

5. The effect of mez on anammox in soil should also be analyzed, the nitrogen cycle related gene should be analyzed completely.

Answer:

Thanks for this comment. We have now analyzed the genes and expressions of the soil anammox process, and also provided a more complete assessment of nitrogen-cycle-related functional genes under Mez treatment (please see the figure below). Results showed that Mez did not significantly promote the anammox-related gene expression under the tested conditions. We have now updated the results in the revised manuscript.

"*We examined the genes and their expression levels across the major nitrogen transformation pathways, including mineralization, nitrification, denitrification, anammox, DNRA, and ANRA (Fig. 2a)*" (Line 155).

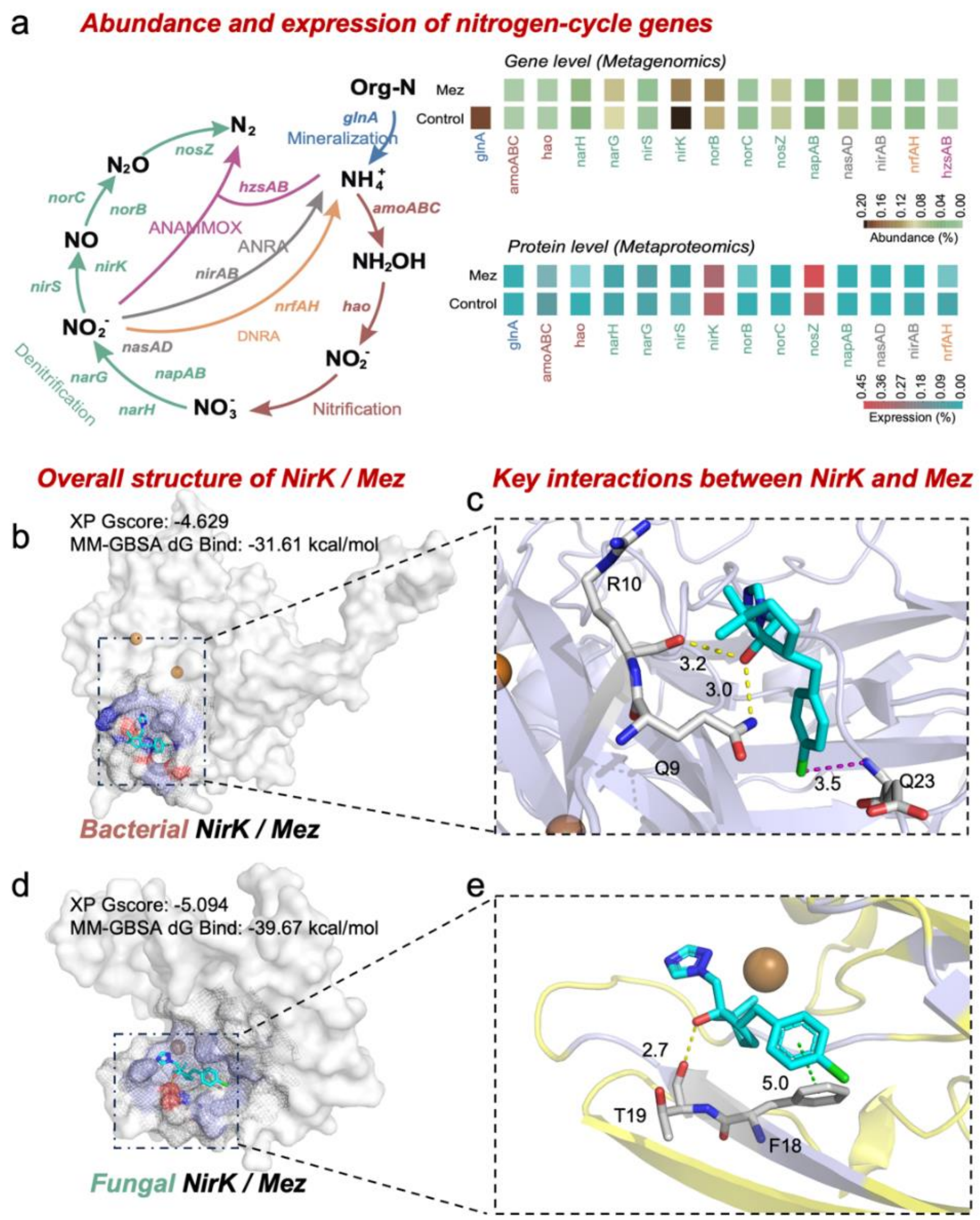

Response to comments Fig 3 (now updated in the main text, **Fig. 2**)**.** Abundances of N-cycling genes and the structure-based model of Mez binding with NirK. (a) Gene and protein abundances of each N transformation in the control and Mez treatments. (b) and (d) Overall structures of the bacterial NirK/ Mez complex (PDB code: 6ZAS) and fungal NirK/ Mez complex (PDB code: 6THE). NirK proteins are represented as surface mode. Mez is shown as sticks (cyan). (c) and (e) Chemical structures of Mez with predicted binding mode. Hydrogen bonds (yellow), Halogen bonds (violet), and π-π stacking interactions (green) between the amino group of Mez (cyan) and NirK (grey) in the binding pocket are shown.

6. The degradation products of mez should be analyzed.

Answer:

Thanks for this suggestion. Mez is a triazole fungicide, and the degradation behavior of triazoles has been widely investigated in previous studies[1-3]. Available toxicological assessments and reports on Mez and structurally related triazole compounds indicate that their degradation metabolites are generally less toxic than the parent compounds[4-7]. Therefore, we focused primarily on the parent compound (Mez) in the present study.

Nevertheless, based on available reports and studies on Mez and structurally related triazole fungicides[8-12], we have now added a hypothetical degradation pathway of Mez, which is presented in the revised manuscript (see figure below).

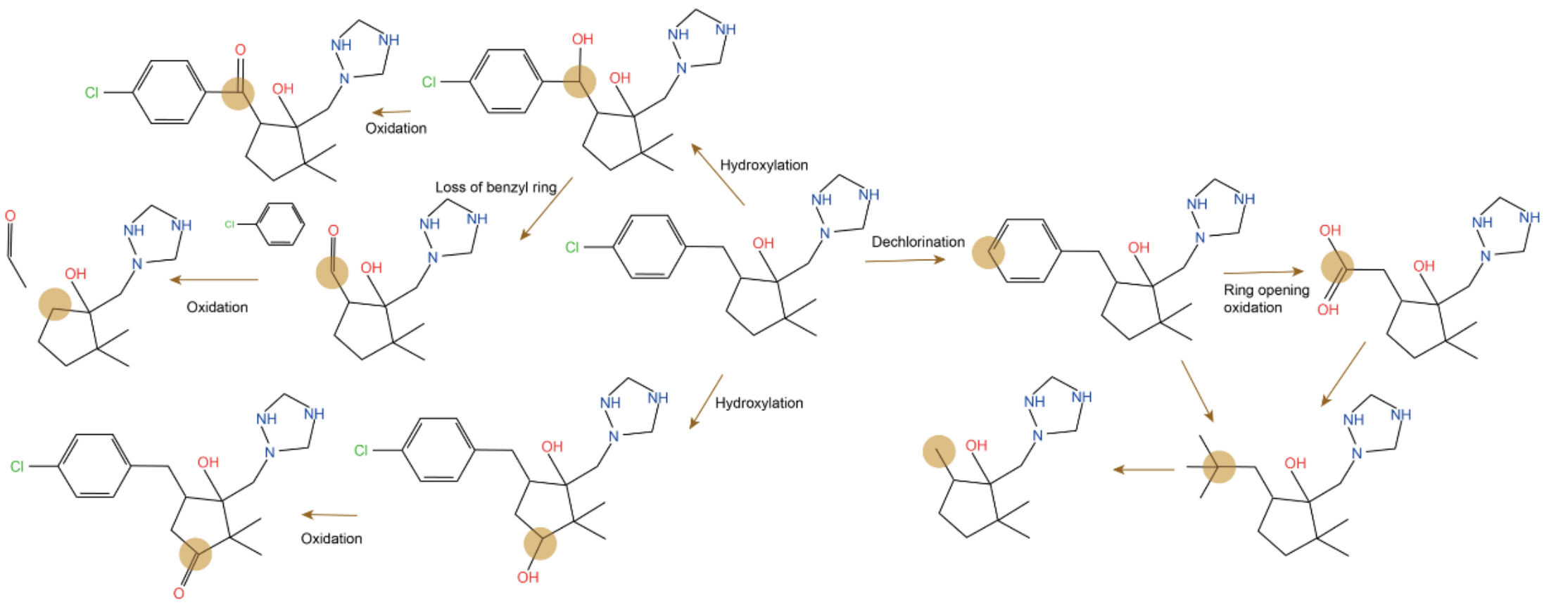

Response to comments Fig 4 (now added in the Supporting Information, **Fig. S8**). Mez potential degradation pathways.

References:

1. Hou, L. et al. Triadimefon in aquatic environments: occurrence, fate, toxicity, and ecological risk. Environmental Sciences Europe 34, 12 (2022).

2. Bian, Y. et al. Triazoles in the environment: An update on occurrence, fate, health hazards, and removal techniques. Environmental Research 271, 121092 (2025).

3. Zou, P.-c. et al. Triazoles in the environment: An update on sample pretreatment and analysis methods. Ecotoxicology and Environmental Safety 286, 117156 (2024).

4. The National Food Safety Standard "Maximum Residue Limits for Pesticides in Food" GB2763.

5. USEPA, 2007. Office of Prevention, Pesticides and Toxic Substances. Pesticide Fact Sheet:

Metconazole, WASHINGTON, D.C.

6. USEPA, 2013. Office of Prevention, Pesticides and Toxic Substances. Pesticide Fact Sheet: Metconazole, WASHINGTON, D.C.

7. (EFSA), E. F. S. A. et al. Peer review of the pesticide risk assessment of the active substance metconazole. EFSA Journal 21, 8141 (2023).

8. Ludovic, L., Stephan, B. & Dominique, W. Photocatalytic degradation of a triazole pesticide, cyproconazole, in water. Journal of Photochemistry & Photobiology A Chemistry 188, 34-42 (2007).

9. Budny, M., Wiodarczyk, J., Muziol, T., Bosiak, M. J. & Wolan, A. Stereoselective Synthesis of Two Potential Metabolites of cis -Metconazole. Tetrahedron Letters 58, 4285-4288 (2017).

10. Kang, D. et al. Identification of transformation products to characterize the ability of a natural wetland to degrade synthetic organic pollutants. Water Research 187, 116425 (2020).

11. He, R. et al. Systematic investigation of stereochemistry, stereoselective bioactivity, and antifungal mechanism of chiral triazole fungicide metconazole. Science of The Total Environment 784, 147194 (2021).

12. Xue, P. et al. Environmental behavior of the chiral fungicide epoxiconazole in earthworm-soil system: Enantioselective enrichment, degradation kinetics, chiral metabolite identification, and biotransformation mechanism. Environment International 167, 107442 (2022).

"*On this basis, we propose a hypothetical intracellular Mez degradation route in which Mez is firstly conjugated and detoxified by GST-related enzymes, generating aromatic intermediates that retain benzene-ring moieties (Supplementary Fig. 8); these intermediates may then be channeled into benzoate degradation pathways for further ring cleavage and catabolism*" (Line 235).

7. The effects of mez on the antibiotic resistance genes should be carried out.

Answer:

Thanks, and we have now quantified the abundances of antibiotic resistance genes (ARGs) across the control, Mez and Dif treatments. Under the tested

conditions and application doses, total ARG abundance did not differ significantly between the Mez treatment and the control (see figure below). In contrast, Dif significantly reduced total ARG abundance relative to the control (P<0.01). These findings suggest that neither Mez nor Dif increases ARG abundance, and that Dif may lower the overall ARG burden. We have now incorporated these results into the revised manuscript.

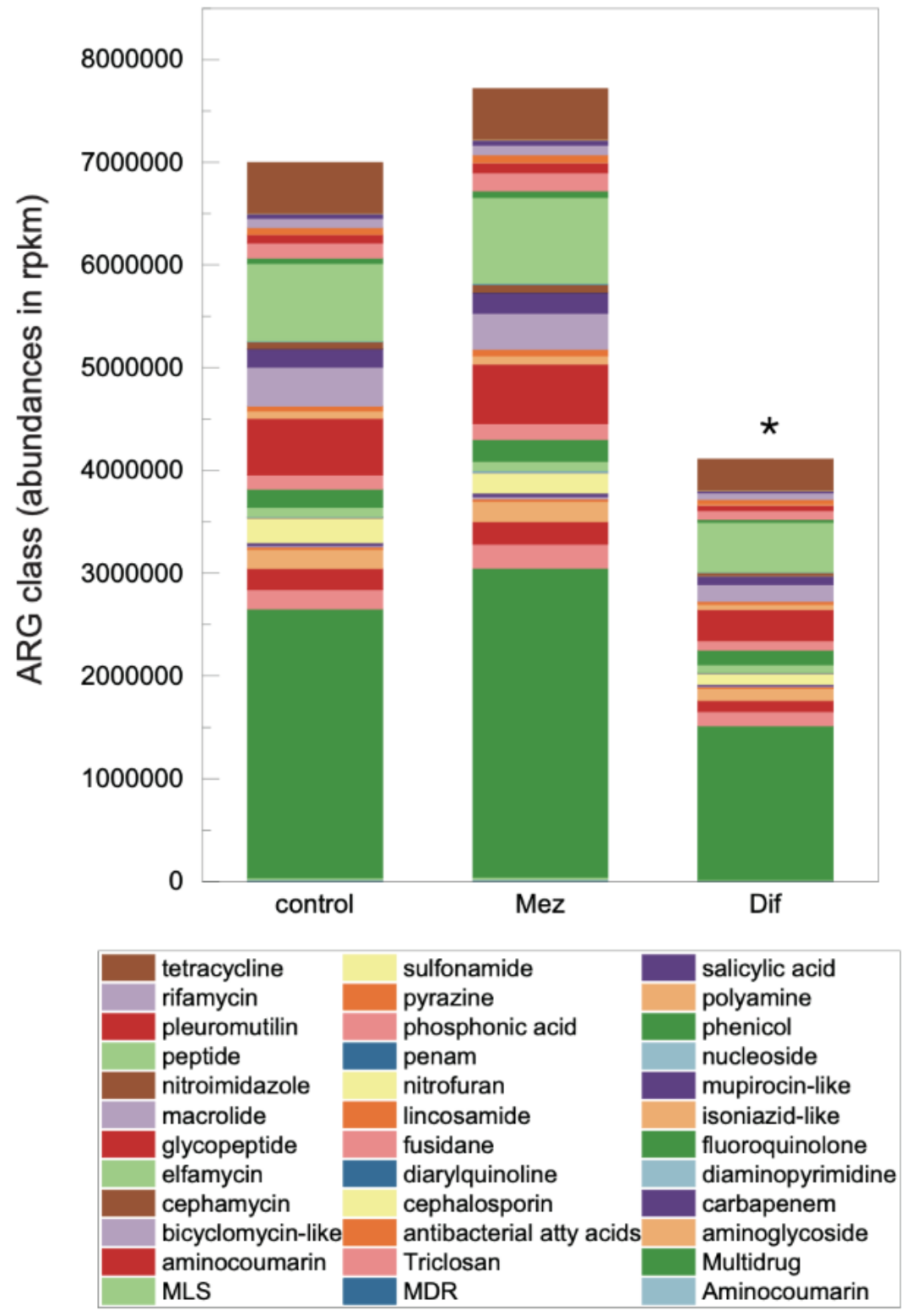

Response to comments Fig 5 (now added in the Supporting Information, **Fig. S19**). The abundance of soil antibiotic resistance genes (ARGs) in response to Mez and Dif treatments.

"*In addition, at the tested doses in this study, neither Mez nor Dif increased the abundances of soil resistance genes (Supplementary Fig. 19), indicating that their application did not promote resistome enrichment.*" (Line 363).

8. The English should be polished.

Answer:

Thanks. Language of the revised manuscript was polished by a native speaker (Dr. Holly Walder, Imperial College London). We have also carefully revised the manuscript throughout to improve clarity and readability.

Replies and Explanations to Comments

Reviewer #1 Comments:

No further comments.

Reviewer #2 Comments:

The authors answered my questions; it can be accepted for publication.

Response:

We are grateful to the two reviewers for their thoughtful and constructive comments. Their comments have significantly enhanced the work.
