# Supplementary material for: Structure-guided identification of denitrification inhibitors to mitigate agricultural N2O emissions
Source: Nat Commun. 2026 Aug 20;17:9944. doi: 10.1038/s41467-026-76975-6 (PMC13586399; doi:10.1038/s41467-026-76975-6)
Supplement: Supplementary file 3 [file 41467_2026_76975_MOESM3_ESM.pdf]

Description of Additional Supplementary Files

File Name: Supplementary Data 1
Description: MAG information, MAG-centric NirK and NosZ expression, and literature used for meta-analyses.

File Name: Supplementary Data 2
Description: The raw data used for plotting figures.
