# Supplementary material for: Structure-guided identification of denitrification inhibitors to mitigate agricultural N2O emissions
Source: Nat Commun. 2026 Aug 20;17:9944. doi: 10.1038/s41467-026-76975-6 (PMC13586399; doi:10.1038/s41467-026-76975-6)
Supplement: Supplementary file 4 [file 41467_2026_76975_MOESM6_ESM.pdf]

nature portfolio

Corresponding author(s): Xinping Chen and Xiaoxuan Su

Last updated by author(s): Jun 29, 2026

Reporting Summary

Nature Portfolio wishes to improve the reproducibility of the work that we publish. This form provides structure for consistency and transparency in reporting. For further information on Nature Portfolio policies, see our Editorial Policies and the Editorial Policy Checklist.

Statistics

For all statistical analyses, confirm that the following items are present in the figure legend, table legend, main text, or Methods section.

n/a	Confirmed	
☐	☒	The exact sample size (*n*) for each experimental group/condition, given as a discrete number and unit of measurement
☐	☒	A statement on whether measurements were taken from distinct samples or whether the same sample was measured repeatedly
☐	☒	The statistical test(s) used AND whether they are one- or two-sided<br>*Only common tests should be described solely by name; describe more complex techniques in the Methods section.*
☒	☐	A description of all covariates tested
☐	☒	A description of any assumptions or corrections, such as tests of normality and adjustment for multiple comparisons
☐	☒	A full description of the statistical parameters including central tendency (e.g. means) or other basic estimates (e.g. regression coefficient) AND variation (e.g. standard deviation) or associated estimates of uncertainty (e.g. confidence intervals)
☐	☒	For null hypothesis testing, the test statistic (e.g. *F*, *t*, *r*) with confidence intervals, effect sizes, degrees of freedom and *P* value noted<br>*Give P values as exact values whenever suitable.*
☒	☐	For Bayesian analysis, information on the choice of priors and Markov chain Monte Carlo settings
☒	☐	For hierarchical and complex designs, identification of the appropriate level for tests and full reporting of outcomes
☒	☐	Estimates of effect sizes (e.g. Cohen's *d*, Pearson's *r*), indicating how they were calculated

Our web collection on statistics for biologists contains articles on many of the points above.

Software and code

Policy information about availability of computer code

Data collection	Data in this study were collected from Nexera UC SFC system (Shimaduz, Kyoto, Japan) with LCMS/MS-8050 (Shimaduz, Kyoto, Japan), Ion Chromatograph (Ion Chromatograph (ICS-3000, Dionex, USA), GC-ECD (Aglient 7890, USA), Illumina Novaseq PE150 platform (Illumina Inc.), Schrödinger (www.schrodinger.com), Biacore-8K (GE-Healthcare, Chicago, IL, USA) etc. Other details see the Methods section.
Data analysis	Most of the analyses were carried out using SPSS version 20.0 (IBM Corp., Armonk, NY, USA). Genome assembly was conducted with MEGAHIT (DOI: 10.1093/bioinformatics/btv033); gene prediction, taxonomy, and function annotation were carried out by Prodigal (https://githu b.com/hyattpd/prodigal/wiki), CD-HIT (http://www.bioinformatics.o rg/cd-hit/, version 4.6.1), bowtie 2 (http://bowtie-bio.sourceforge.net/bowtie2/index.shtml), and Diamond (http://w ww.diamondsearch.org/index.php, version 0.8.35); binning was performed with vamb (https://github.com/RasmussenLab/vamb); the species classification annotation and phylogenetic tree construction of bins were performed using GTDB (https://gtdb.ecogenomic.org/). The phylogenetic tree was visualized using iTOL (https://itol.embl.de). The docking results were visualized using Pymol (version 2.5.4). Graphical visualizations were created using GraphPad Prism version 7.0 (GraphPad Software, San Diego, CA, USA), Origin (version 2019), R (version 3.1.2) softwares, and Adobe Illustrator (Adobe Inc., USA). Additional details are described in the Methods section.

For manuscripts utilizing custom algorithms or software that are central to the research but not yet described in published literature, software must be made available to editors and reviewers. We strongly encourage code deposition in a community repository (e.g. GitHub). See the Nature Portfolio guidelines for submitting code & software for further information.

Data

Policy information about availability of data

All manuscripts must include a data availability statement. This statement should provide the following information, where applicable:

- Accession codes, unique identifiers, or web links for publicly available datasets
- A description of any restrictions on data availability
- For clinical datasets or third party data, please ensure that the statement adheres to our policy

Metagenome sequencing data have been deposited in the GSA, China National Center for Bioinformation with the accession number CRA045584 (https://ngdc.cncb.ac.cn/gsa/browse/CRA045584). Metaproteome data have been deposited in the OMIX, China National Center for Bioinformation with the accession number OMIX018172 (https://ngdc.cncb.ac.cn/omix/release/OMIX018172). Supplementary Data 1 are deposited in figshare (https://doi.org/10.6084/m9.figshare.28636514). Supplementary Data 2 are deposited in figshare (https://doi.org/10.6084/m9.figshare.32817770).

Research involving human participants, their data, or biological material

Policy information about studies with human participants or human data. See also policy information about sex, gender (identity/presentation), and sexual orientation and race, ethnicity and racism.

Reporting on sex and gender	Not involved.
Reporting on race, ethnicity, or other socially relevant groupings	Not involved.
Population characteristics	Not involved.
Recruitment	Not involved.
Ethics oversight	Not involved.

Note that full information on the approval of the study protocol must also be provided in the manuscript.

Field-specific reporting

Please select the one below that is the best fit for your research. If you are not sure, read the appropriate sections before making your selection.

☐ Life sciences ☐ Behavioural & social sciences ☒ Ecological, evolutionary & environmental sciences

For a reference copy of the document with all sections, see nature.com/documents/nr-reporting-summary-flat.pdf

Ecological, evolutionary & environmental sciences study design

All studies must disclose on these points even when the disclosure is negative.

Study description	We investigated the potential of the triazole compound metconazole in reducing agricultural N_2O emissions through a series of laboratory-scale experiments. Our findings demonstrate that metconazole effectively suppresses N_2O emissions by inhibiting fungal denitrification. Using molecular docking, molecular dynamics simulations, and Surface Plasmon Resonance (SPR), we identified P450Nor as the regulatory target of metconazole in this pathway. Building on these insights, we screened and identified difenoconazole as a more potent inhibitor of P450Nor. Subsequent validation at the microbial protein, cellular, community, and field levels confirmed its superior efficacy in reducing soil N_2O emissions.
Research sample	In this study, we selected paddy field soil, the previously isolated denitrifying fungal strain Penicillium janthinellum SM-12f4, and the P450Nor protein expressed by the fungus Fusarium oxysporum as our research subjects.
Sampling strategy	For the 28-day experiments, concentrations of NO_3^-, NO_2^-, N_2O, and metconazole residues were monitored throughout the incubation period. At the conclusion of the experiment, soil DNA and total protein were extracted for subsequent metagenomic sequencing and metaproteomics analysis (sample size: n = 4 for each condition, with biological replicates; further details are provided in the Methods section). For the 7-day experiments, NO_3^-, NO_2^-, NO, and N_2O concentrations were measured at 6-hour intervals during incubation (sample size: n = 4 for each biological replicate; further details are provided in the Methods section). In the validation experiments, NO and N_2O concentrations were monitored during the 7-day pure culture incubation of the fungal strain Penicillium janthinellum SM-12f4 (sample size: n = 4 for each biological replicate), the soil microsome incubation (sample size: n = 4 for each biological replicate), and the 21-day paddy field experiments (sample size: n = 4 for each biological replicate). Statistical differences between treatment groups were analyzed using one-way ANOVA, followed by Tukey's HSD post-hoc test for pairwise comparisons (significance level: $P < 0.05$). Data normality was evaluated with the Shapiro-Wilk test, and homogeneity of variances was assessed using Levene's test. When the data did not meet parametric assumptions, the Kruskal-Wallis test was applied.
Data collection	Chen X.P., Deng Y., and Su X.X. conceived and designed the study. Su X.X., Chen X.P., and Zhu Y.G. supervised the study and acquired funding. Deng Y., Zeng H.X., Fang L.F., and Liu, B. performed experiments and meta-analysis. Xiao R., Zhang J.B., and Zhang H.L. acquired sequence data. Deng Y., Zhang L., and Yang L.Y conducted the molecular docking, molecular dynamic simulation, and SPR experiment. Deng Y. carried out all data analyses and wrote the manuscript, with contributions from Su X.X., Chen X.P., Hou L.J.,

Prakash L., Wang Y.M., and Zhang F.S.

Timing and spatial scale: All samples and experiments were collected and conducted from June to August of 2022 and 2024.

Data exclusions: No data exclusion.

Reproducibility: All measurements were carried out in triplicate or quadruplicate. All samples were repeatedly collected from each paddy field. All attempts to repeat our experiments were successful.

Randomization: Samples for each measurement were randomly collected and allocated in this study.

Blinding: As both the experiments and the analyses were carried out by the same group of scientists, blinding was not relevant for this study.

Did the study involve field work? [x] Yes [ ] No

Field work, collection and transport

Field conditions: The study areas are located in paddy fields in the National Purple Soil Fertility and Fertilizer Benefit Monitoring Base of Southwest University, Beibei District, Chongqing, China (29°48’36” N, 106°24’33” E). This region experiences a subtropical monsoon climate with an average annual temperature of 18.3°C and an average annual rainfall of 1115.3 mm.
Surface soil samples (0-20 cm depth) were collected in June 2022. After collection, the soil samples were stored at 4°C in ice boxes and transported to the laboratory. Water chemical characteristics were analyzed within 24 hours. N_2O samples were stored in sealed airbags and their concentrations were measured within 24 hours. The organic matter (SOM), total nitrogen (TN), and total phosphorus (TP) of soil samples were 17.47 ± 2.90, 1.17 ± 0.02, and 0.72 ± 0.02 g/kg, respectively. The pH of soil samples was 6.83 ± 0.10.

Location: The National Purple Soil Fertility and Fertilizer Benefit Monitoring Base of Southwest University, Beibei District, Chongqing, China (29°48’36” N, 106°24’33” E).

Access & import/export: The collections of samples in this study did not involve sensitive or prohibited areas.

Disturbance: No disturbance was caused by this study.

Reporting for specific materials, systems and methods

We require information from authors about some types of materials, experimental systems and methods used in many studies. Here, indicate whether each material, system or method listed is relevant to your study. If you are not sure if a list item applies to your research, read the appropriate section before selecting a response.

Materials & experimental systems

n/a	Involved in the study	
[x]	[ ]	Antibodies
[x]	[ ]	Eukaryotic cell lines
[x]	[ ]	Palaeontology and archaeology
[x]	[ ]	Animals and other organisms
[x]	[ ]	Clinical data
[x]	[ ]	Dual use research of concern
[x]	[ ]	Plants

Methods

n/a	Involved in the study	
[x]	[ ]	ChIP-seq
[x]	[ ]	Flow cytometry
[x]	[ ]	MRI-based neuroimaging

Plants

Seed stocks: Not involved.

Novel plant genotypes: Not involved.

Authentication: Not involved.
